# e-Nature Positive Emotions Photography Database (e-NatPOEM): affectively rated nature images promoting positive emotions

**DOI:** 10.1038/s41598-021-91013-9

**Published:** 2021-06-03

**Authors:** Daniela Dal Fabbro, Giulia Catissi, Gustavo Borba, Luciano Lima, Erika Hingst-Zaher, João Rosa, Elivane Victor, Letícia Bernardes, Tinely Souza, Eliseth Leão

**Affiliations:** 1 Hospital Israelita Albert Einstein, Research Institute, São Paulo, Brazil; 2 Department of Electronics-DAELN, Graduate School On Biomedical Engineering-PPGEB, Federal University of Technology-Paraná-UTFPR, Curitiba, Brazil; 3 Instituto Butantan, São Paulo, Brazil; 4 National Geographic Brasil, São Paulo, Brazil

**Keywords:** Health care, Quality of life

## Abstract

Affectively rated image databases have their main application in studies that require inducing distinct stimuli on subjects. Widespread databases are designed to cover a broad range of stimuli, from negative to positive (valence), and relaxed to excited (arousal). The availability of narrow domain databases, designed to cover and thoroughly analyze a few categories of images that induce a particular stimulus, is limited. We present a narrow domain affective database with positive images, named e-Nature Positive Emotions Photography Database (e-NatPOEM), consisting of 433 high-quality images produced by professional and amateur photographers. A total of 739 participants evaluated them using a web-based tool to input valence-arousal values and a single word describing the evoked feeling. Ratings per image ranged from 36 to 108, median: 57; first/third quartiles: 56/59. 84% of the images presented valence > middle of the scale and arousal < middle of the scale. Words describing the images were classified into semantical groups, being predominant: Peace/tranquility (39% of all words), Beauty (23%), and Positive states (15%). e-NatPOEM is free and publicly available, it is a valid resource for affective research, and presents the potential for clinical use to assist positive emotions promotion.

## Introduction

The perception that contact with nature provides harmony and balance to humans is ancient. However, after the industrial revolution, human societies became increasingly urban, with more than half of the world’s population living in urban areas^1^. People started moving progressively away from contemplative moments out in nature and hence from their potential physical, mental, and emotional benefits.

The urban environment exposes people to many stressors^2^ leading to physical and mental illness, including cardiovascular disease, obesity^3^, and burnout^4,5^. This impact on health has led to the need to understand and prevent the detrimental effects of living in urban areas^6^ and explore whether a closer contact with nature may benefit the health of individuals living in such environments^7^.

In recent decades there has been growing scientific interest in understanding the effects of being around natural elements, including in hospitals and other health care settings, where contact with nature may have restorative effects, promote better health conditions and improved quality of life^7^.

A review of 57 published studies revealed that the benefits of interacting with nature have been poorly investigated by the health sciences, whereas social and environmental sciences have been much more heavily represented. It is also noteworthy that the studies were heavily biased towards North America and Europe (79%), and no studies were located in South America or Africa^8^, even though the latter two regions have the countries with the greatest biodiversity on the planet.

It is believed that humans have a predisposition to appreciate the contact with other living organisms, a hypothesis that has been named biophilia^9^. More recently, authors expanded this concept to suggest that humans have an innate bond with nature that goes beyond animals and which includes plants and landscapes^7^. The relationship between human and nature has been explored for therapeutic purposes since the first European hospitals were stablished^10^.

Depending on the circumstances in which human-nature interaction occurs, it is usually classified as follows^8^. (1) Indirect: viewing a photography, painting, or even real nature from a window, for example. Physical presence within nature is not required. (2) Incidental: there is physical presence, however, the contact occurs unintentionally, e.g., when encountering a plant in the office. (3) Intentional: there is physical presence and the contact is deliberated, e.g., going out for a walk in the woods.

Interaction with nature is, for many people, an effective stress-relieving strategy^11^. Several studies point out that this interaction may promote *health* and *well-being* in different aspects, including: psychological—positive effects on, e.g., self-esteem, mood, anxiety; cognitive—positive effects on, e.g., mental fatigue, attention, productivity; and physiological—positive effects on, e.g., blood pressure, cortisol levels, stress^8,12^.

The term *health* is defined by the World Health Organization as a state of complete physical, mental, and social well-being, rather than the absence of disease^13^.

As for *well-being* within a policy context, it is considered a physical, social and mental state rather than the absence of pain, discomfort and/or disability^1^.

*Emotions* result from synchronized and interrelated changes in response to stimuli that the individual evaluates as having some relevant meaning. It is this meaning that will cause the emotion to emerge, which will be more or less intense according to the relevance value attributed to the event. *Positive emotions* are a reference to a set of emotions that are related to pleasant, that understand the situation as beneficial and remain in a short time^14^. Both high arousal positive emotions and low arousal positive emotions have been proven to be related with well-being^8,15,16^.

Despite evidence of a positive effect of natural environments on health, it is not always possible to be in contact with nature to enjoy its potential benefits. Illness processes represent a particular situation in which individuals are unable, sometimes against their will and for prolonged periods of time, to interact with natural environments.

Having a window in the inpatient unit overlooking a natural landscape has been shown to have a potential therapeutic benefit in the recovery of surgical patients by reducing the amount of painkillers and shortening hospital stays^17^.

But hospital buildings in an urban context do not always provide a “window with a view”. Health care centers, especially those with no green spaces where patients could enjoy their benefits, have sought to promote other forms of contact with the natural world that provide some sense of well-being during hospitalization, including the use of photographs and videos from an indirect interaction perspective.

The use of audiovisual resources in the health care environment has been under investigation for some time and commercially available devices that create “virtual windows” have also been developed^18^. However, access to these resources in health care settings is still limited, either due to a lack of knowledge or economic and financial constraints.

Randomized clinical trials using nature-based audiovisual interventions are scant but have shown reduced abdominal discomfort during sigmoidoscopy^19^ and reduced pain and anxiety during dressing changes^20^. However, these studies had small sample sizes and methodological flaws related to randomization and blinding and used poorly established criteria for image selection^21^.

If on the one hand the benefits of viewing images of nature in promoting positive emotions has been investigated and had its potential in treatment and in the production of emotions demonstrated, on the other hand there is a difficulty, in clinical practice, in selecting images which can aid in different clinical settings because there is no single database of pictures that has been validated and designed specifically for this purpose.

There is an apparent lack of defined criteria in the literature for selection of the images used. These normally include thematic and landscape pictures of beaches, mountains, and forests depicting the natural environments of the countries where they were taken, which raises some questions about the constituent elements of these images. Would the contribution of each natural element in the same landscape be similar? For example, would viewing images of bird be any different to viewing images of flower? Which of these images, among other visual motifs of nature, would have the greatest potential to promote any benefit? What kind of aesthetic experience does each natural element in the image trigger in the viewer? Thus, some indicator is required to guide the selection of images for studies investigating their use in clinical practice.

It should be noted that the largest validated databases of pictures for studying emotion may only be used by researchers conducting experiments and its images can neither be shown in any media outlet or publications. The International Affective Picture System (IAPS) is a database depicting various aspects of real life (sports, fashion, landscape, violence, etc.), designed to elicit emotional states and that can be easily presented in the experimental context of the laboratory, enabling precise control over the timing and duration of exposure^22,23^. The Nencki Affective Picture System (NAPS) database is designed to use in the same context and, as well as IAPS, may only be used by researchers conducting experiments and its images can neither be shown in any media outlet or publications. NAPS comprises five categories (People, Faces, Animals, Objects, Landscapes)^24^.

Other databases of images, more permissive regarding the terms of use, that have been used in emotional research are the Geneva Affective Picture Database (GAPED)^25^, EmoPics^26^, Open Affective Standardized Image Set (OASIS)^27^. These databases vary in the number and quality of the images, and can be classified as broad domain databases because that they were designed to cover a large range of affective visual stimuli, from negative to positive in the valence dimension, and relaxed to excited in the arousal dimension.

Regarding narrow domain databases, i.e., databases specialized in a certain category of images or in a specific range of emotional stimuli, the following apply: Open Library of Affective Foods (OLAF)^28,29^ presents pleasant and unpleasant images of foods, for research with healthy individuals or those with eating disorders and obesity; Military Affective Picture System (MAPS)^30^ presents images from military scenarios, for use mainly with individuals with combat-related psychopathology, and DIsgust-RelaTed-Images (DIRTI) Database^31^, which presents only disgust-inducing images from categories such as animals, injuries/infections and death, for research related to different aspects of disgust.

This study aimed to develop and validate a database of affectively rated nature images that promote positive emotions and have this database available for future research and use in health care settings, mainly with hospitalized patients, which commonly feel apprehensive and can benefit from positive stimuli^32^. In this article we present the e-Nature Positive Emotions Photography Database (e-NatPOEM) comprising 403 high-resolution (1600 × 1200 pixels) photographs focused on positive valence, and grouped into nine general categories: (1) Landscape, (2) Water, (3) Forest/woodland, (4) Pale Bird, (5) Colorful Bird, (6) Sky, (7) Flower, (8) Insects, and (9) Sea. The images were also grouped plus five attribute groups based on the participants' experience: (1) Beauty (aesthetic experience), (2) Peace/tranquility, (3) Positive states, (4) Miscellaneous, and (5) Negative states.

e-NatPOEM is publicly available, can be shown in any media outlet or publications, and has important features that distinguish it from other affectively-rated databases: 400+ high-quality nature images of several categories, classified into distinct attribute groups.

## Methods

### Study design

The web-based methodological study was conducted by the Teaching and Research Institute at the Albert Einstein Jewish Hospital (HIAE), São Paulo, Brazil.

### Ethical aspects

According to Resolution 466/2012 of the Brazilian National Health Council (CNS), which approves guidelines and regulatory standards for research involving human beings in Brazil, the project was submitted and approved by the Research Ethics Committee of Albert Einstein Jewish Hospital under registration number no. 64096816.9.0000.0071-03/02/2017.

The Informed Consent Form was applied only the online study platform. All research participants read and accepted the Informed Consent Form, and the document of the same content signed by the principal investigator was sent by email for archiving by the study participant. The access to research was allowed only for those who agreed to the informed consent form.

### Nature images

Over 700 high-resolution (1600 × 1200) photographs were sent to two independent photographers who selected the best pictures according to photography rules relating mainly to^33–36^:

- technique—the basic requirements of photography: exposure, focus, and frame adjustment;
- information—whether the photograph fulfills its role of conveying the message of what was portrayed in the image;
- creativity—the visual and aesthetic impact generated from the combination of technique and information in the creation of the image.

These criteria were adopted considering that the aesthetic experience comes from the quality of the photographic material, which influences the perceptual experience of the viewers.

We sought to study natural elements separately, therefore we separated the images of nature into categories as we wanted to explore how the affective response to different elements of nature differ from each other. The selection of the nine categories was based on the experience and familiarity of the researchers with nature photography, being also inspired by categories adopted by other existing image datasets^22–27^. In the animal category, we chose to work only with birds that were either pale or colorful because it allowed us to observe the emotions associated with color, since colors influence emotions^37^.

This analysis resulted in the original e-NatPOEM database comprising 403 images licensed by the researchers and/or nature photographers who coauthored this study. The photographs were grouped into nine categories: Pale Bird (50 images), Colorful Bird (50), Sky (50), Flower (50), Sea (50), Insects (50), Water (50), Landscape (27), and Forest/woodland (26).

Images in the control group were obtained from the internet (a complete description of each image is presented in Supplementary Material 1) and consist of 28 images such as snakes, spiders, degraded nature that could be of low valence and high alert, as already described for some images at IAPS^23^.

### Online platform

A web tool was developed in partnership with a tech company under the supervision of the authors. The tool, similar to the assessment scales of other picture databases^24,25^, was developed to capture the valence and arousal ratings assigned to a set of images by the study participants.

Even though the Self-Assessment Manikin (SAM) by Bradley and Lang^38^ is a pictorial assessment technique widely used in emotion studies, participants have had difficulty in understanding the meaning of its pictographic representations, hence raising a pragmatic concern about the current intuitiveness of SAM. The paper-and-pencil design principles, upon which SAM was based, are distant from the advanced interfaces, digital media, social networks, and mobile applications that have currently shaped new paradigms of interaction^39^. The Affective Slider (AS) scale used in the current study was designed to overcome these limitations in the self-assessment of emotion. The AS is composed of two slider controls that measure basic emotions in terms of valence and arousal on a continuous scale. The AS shows a strong correlation with SAM both for valence and arousal ratings with two additional advantages: the AS does not require written instructions and it can be easily reproduced in latest-generation digital devices, including smartphones and tablets^39^.

Each image was randomly selected from the picture database using a random number generation function with uniform distribution. Considering that a large number of ratings could be disproportionally submitted for a single image, we aimed to ensure that a similar number of ratings were submitted per image through a routine that evaluated the number of ratings already submitted for each image to favor images with fewer ratings.

Support tests for the scale were performed using the following technologies: IE 10+; Chrome 32+; Safari 6+; Firefox 3.5+; iOS: 8.0+; and Android: 4.2+.

The online platform consisted of a screen with a brief presentation of the experiment in colloquial language, describing the steps for participating in the study and rating the images, emphasizing the importance of participating and reading the consent form, the concentration required for rating each image and a quiet setting without distractions. By scrolling the page to the bottom, participants would click on the “start research” button and answer sociodemographic questions for the characterization of the sample. Participants were required to read and accept the consent form to proceed to the following section.

In the following section, the images were randomly presented to each and across participants, with the picture with the fewest ratings displayed first. The high-resolution pictures were displayed in full-screen mode for 9 s. During this short period the participant contemplated the image for rating experienced valence and arousal. The image was then scaled down and the affective dimensions were displayed randomly on the left or right sides of the screen to prevent automaticity in the rating process, which had no time limit.

To minimize any effects of the previous picture, a 5 s black mask covering the entire screen space was displayed between each trial to neutralize the affective states triggered by the previous image and to not affect the following trial.

The web browser was set in automatic full-screen mode. In smartphones and tablets, the images were displayed centered on the screen.

**Figure 1:**
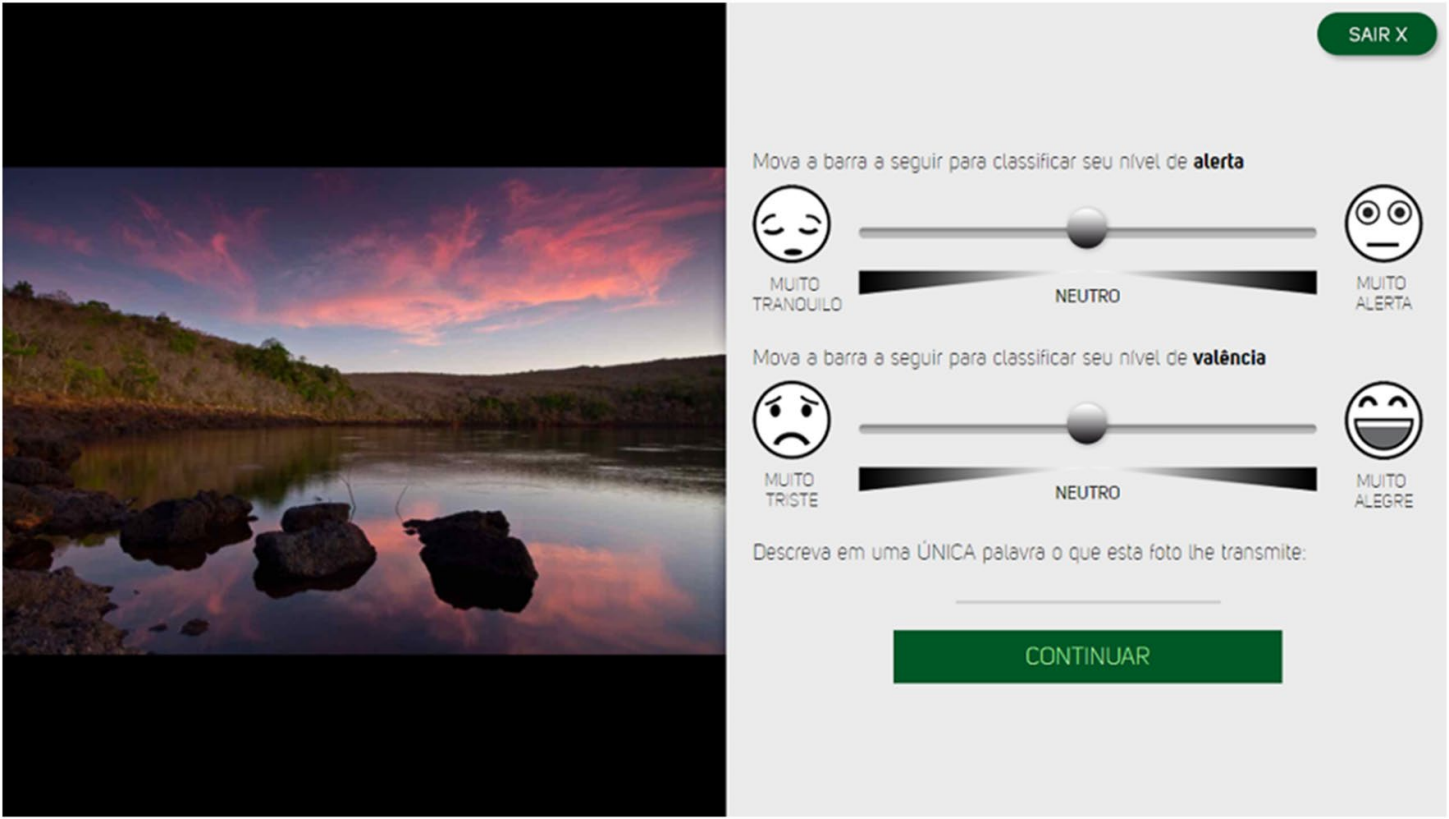
Screenshot showing the digital self-assessment tool designed for rating pictorial stimuli. Participants assigned valence (ranging from very unhappy to very happy) and arousal (ranging from very relaxed to very excited) ratings using the continuous sliders. Portuguese version only, assessments were provided by Brazilian participants.

Images were rated using a 9-point (1–9) bipolar semantic continuous sliding scale for better visualization, ease of use in digital devices, and more intuitive understanding^39^. The AS is composed of two separate monochromatic slider controls with the emoticons placed at the two extremities. Figure 1 shows a screenshot of the digital self-assessment scale for rating the images. The first slider measuring arousal from very relaxed to very excited, and the second slider measuring pleasure from very unhappy to very happy, with the slider thumbs always placed at the center of the tracks (it is important to mention that there are other bipolar pleasure (valence) scales, distinct from unhappy/happy^40^). Participants could only proceed to the following picture after rating the two affective dimensions. It is worth mentioning that the order of the sliders was randomly presented, arousal above and valence below, or vice versa, to discourage potential biases in ratings (potential automaticity in the rating process)^39^.

To proceed to the following trial, participants were also required to describe in one word, into a field to freely write, what the picture conveyed while viewing it, and the pictures were later grouped according to their qualitative attributes.

An “exit” button was displayed throughout the experiment providing participants with an option to terminate participation; by clicking on the button, participants would read “by choosing to terminate participation, you will no longer be able to continue”. Participants could rate as many images as they wished before clicking on the “exit” button or closing the study page. It was not possible to click back to check previous images.

Initially, the images were grouped into sets of 50 pictures and participants were asked at the end of each set if they would like to continue, with a message displaying the number of the picture in the set (e.g., 1 of 50). However, this information was removed from the experimental protocol because it could compel participants to complete all 50-picture trials and influence their decision to terminate participation at the end of each picture set. Thus, the pictures were presented continuously without the image numbers until participants wished to terminate the experiment.

Upon completion of the trials, participants were directed to a thank you page on behalf of the institution acknowledging their participation.

### Administration panel

An admin panel was built into the software that enabled us to check the accesses and number of ratings submitted per image and per participant.

The images were opened by category showing the total scores, mean rating values, and the list of image attributes. We also had access to the answers of each participant separately to analyze the ratings on an individual basis.

We could also select how the data were downloaded, either with the sociodemographic data and affective ratings exported separately or combined into Excel spreadsheets and graphs with each rating corresponding to a point in the graph and one axis for each affective dimension (valence and arousal). The system could also generate a graph for each picture showing in which quadrant the ratings were clustered.

### Participants

The sample population from Brazil consisted of health care students and professionals, in addition to adult (> 18 years) men and women from the general population who voluntarily accepted to participate in the study. The sample size was calculated on the basis of the minimum number of ratings that each image should receive in the validation process, which was determined from the mean ratings of valence and arousal for each image with 95% confidence intervals. The estimation of mean valence and arousal ratings enabled us to rank the images of interest for the database, i.e., those with low arousal and high valence for positive emotions promotion.

Sample size calculation was based on the study by^24^, who rated valence and arousal on a 9-point scale and reported a mean valence for the entire volunteer population of 5.39 with a standard deviation of 1.63 and mean arousal of 5.10 with a standard deviation of 1.06. Assuming a margin of error of 5% with a 95% confidence interval and a standard deviation of 1.63, the minimum sample size required was 41 ratings per picture.

Validation studies are usually conducted in healthy volunteers for later application in target patient populations^24^. Participants were recruited among staffers and students of the nursing and medical schools and students from other undergraduate courses at our institution. For the general population, the call for participation was made through a standalone social media page of the project, which resulted in 124,196 people reached, 610 shares, 7798 reactions, and 173 comments. The recruitment strategies resulted in a final sample of 739 participants and 27,389 ratings submitted.

### Data analysis

In the data pre-processing phase, we identified 948 accesses to the system. However, by tracking their e-mail address, we observed that some participants accessed the system more than once. The number of accesses ranged from 1 to 8, as follows: 707 unique e-mail addresses with one access each, 73 e-mail addresses with two accesses, 10 e-mail addresses with three accesses, four e-mail addresses with four accesses, three e-mail addresses with five accesses, two unique e-mail addresses with six and seven accesses each, and one e-mail address with eight accesses to the system.

Thus, we identified 802 unique e-mail addresses (participants) and 948 accesses to the system, but not all accesses were included in the affective ratings database. For example, accesses that yielded no ratings were not included in the ratings database.

The initial affective ratings database comprised 28,839 ratings in total. Because the same participant might have accessed the system more than once, we created a new ID for each participant using their e-mail address and checked these addresses against the ratings database to determine the occurrence of repeated ratings by the same participant. In most cases, participants rated each image only once, but 297 participants rated the same image twice, 14 participants rated one image three times, and one image was rated 22 times by the same participant. Thus, to avoid any inconsistency in the data, we excluded all repeated ratings. In total, 658 [(297 × 2) + (14 × 3) + (1 × 22)] repeated ratings were excluded, resulting in a database of 28,181 ratings (28,839 − 658).

We also identified 46 repeated images in the picture database and, because of that, 396 cases in which the same image was rated twice by the same participant. These ratings were also excluded, and the final database comprised 27,389 ratings [28,181 − (396 × 2)].

Categorical variables are expressed as absolute frequencies or percentages and quantitative variables are expressed as means and standard deviations^41^. The mean values and 95% confidence intervals of valence and arousal ratings were calculated using generalized estimation equation (GEE) models with gamma distribution and compound symmetry correlation structure^42^. These models consider valence (or arousal) as an outcome whereas gender, image categories, age and any other aspects are considered explanatory variables. Thus, we can verify through Wald’s tests whether each variable has evidence of an association with the outcome. We chose these models in order to account for data asymmetry and correlation between different ratings of the same individual. In addition, the Gamma distribution was the best fit for the data according to goodness of fit criteria.

Group comparisons were performed using the same models with results expressed as percent difference to the reference group mean or mean ratios, 95% confidence intervals, and p-value. The goodness of fit of the models was assessed using the residual deviance and the Akaike information criterion (AIC)^43^. All analyses were performed using the geepack package^44^ in R software^45^. Due to the large number of ratings in the final analysis (27,389), a p < 0.001 was considered significant.

In addition to the valence and arousal ratings, we asked for the participants to describe in one word, into a field to freely write, what the picture conveyed while viewing it. The semantic analysis and categorization by affinity of the words attributed to the images by the participants was conducted by three researchers, using the triangulation method^46^, aiming to carry out a systematic comparison of the researchers’ analyses to avoid potential subjective distortions. This procedure was supervised by one of the researchers that is specialized in Portuguese Language.

The procedure was as follows: from the list with the 4,702 words attributed to the images by the participants, it was observed that the sum of the occurrences of the three most cited words—“peace” (1166), “beauteous” (1003) and “tranquility” (523), corresponded to 57% of the total. Since these words clearly stood out from the remaining, they were used as a criterion for the creation of categories. The three researchers agreed that “peace” and “tranquility” belong to a same semantic category, named here Peace/tranquility, and Beauteous led to the creation of a second category, named Beauty. Due to the diversity of the remaining words, three broad categories were created to situate them: Positive states, Negative states and Miscellaneous (for words that cannot be clearly associated to positive or negative states). Excluding “peace”, “beauteous” and “tranquility”, which already had their categories, all the other words were classified as belonging to one of the five mentioned categories, by the three researchers, independently. If the three researchers were not unanimous in their classifications, the word was discussed with the help of a language dictionary, for debate and consensus about the classification.

## Results

### About the database

The final database comprised 27,389 ratings from 739 participants (unique e-mail addresses) after excluding repeated accesses and ratings by the same participant. The number of ratings per participant ranged from 1 to 249 with a median of 31 ratings per participant. Four hundred thirty-three images—403 nature-related that compose e-NatPOEM + 28 for control purposes during the assessment procedures—were rated and the number of ratings per image ranged from 36 to 108 with a median of 57 ratings per image (first quartile: 56, third quartile: 59). Participant characteristics are summarized in Table 1.

**Table 1:** Participant characteristics (n = 739).

	N (%)
Number of volunteers	739
**Gender**	
Female	581 (78.6)
Male	158 (21.4)
**Age (years)**	
Mean (standard deviation)	39.5 (14.2)
**Contact with nature**	
Never	16 (2.2)
Rarely	68 (9.2)
Infrequently	201 (27.2)
Frequently	283 (38.3)
Very frequently	171 (23.1)
**Education level**	
Illiterate	4 (0.5)
Primary school	41 (5.5)
Secondary school	115 (15.6)
University undergraduate	252 (34.1)
University postgraduate	212 (28.7)
Master’s degree	78 (10.6)
Doctorate degree	37 (5.0)
**Monthly income**^**a**^	
≤ US$ 176.00	50 (6.8)
US$ 176.01–880.00	240 (32.5)
US$ 880.01–1760.00	165 (22.3)
> US$ 1760.00	94 (12.7)
NA	190 (25.7)

^a^Approximate conversion from Brazilian Real to American Dollar on May 20, 2020.

### Validation—valence and arousal

**Figure 2:**
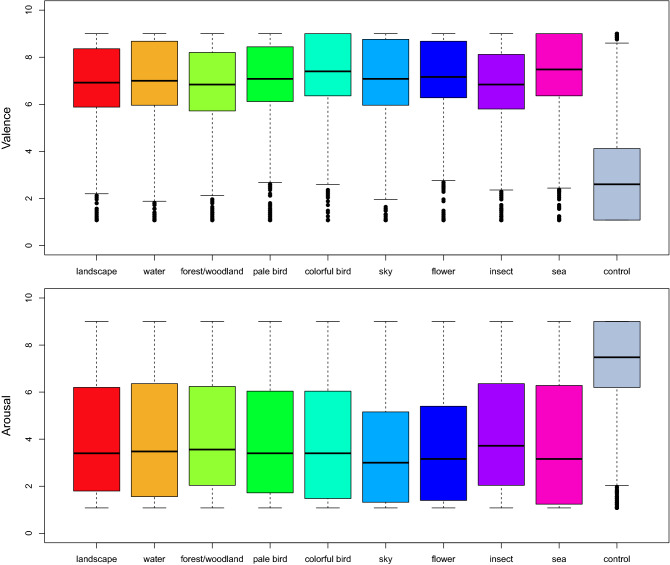
e-NatPOEM valence and arousal ratings by image category (n = 27,389).

Among the 433 pictures rated, there were 50 images from each of the following categories: Pale Bird, Colorful Bird, Sky, Flower, Sea, Insects, and Water, in addition to 27 images of Landscape, 26 of Forest/woodland, and 28 control images. Valence and arousal ratings of database pictures were consistently different from ratings of control images: valence ratings were higher than control image ratings for all image categories, whereas arousal ratings were lower than control ratings for all categories (Fig. 2). The valence and arousal ratings of the pictures are summarized by image category in Supplementary Material 2.

**Figure 3:**
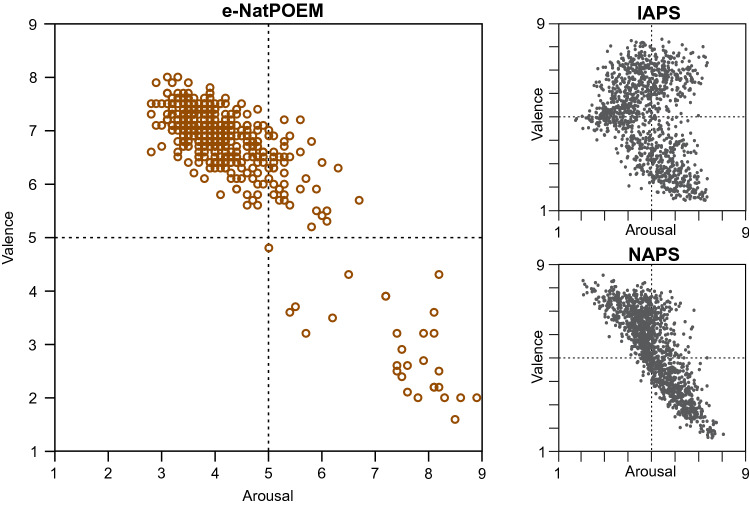
Affective space of the e-NatPOEM (including control images) with the estimated mean ratings of valence and arousal for each image. The affective spaces of the IAPS and NAPS databases are also presented, for comparison.

**Figure 4:**
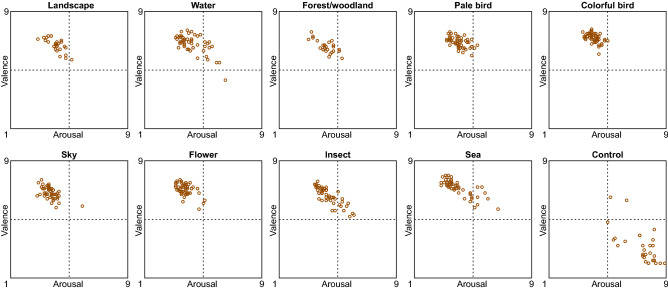
Affective spaces with the estimated mean ratings of valence and arousal for the e-NatPOEM images, by category.

The mean ratings of valence and arousal calculated for each picture of the e-NatPOEM are shown in the affective space in Fig. 3. Valence ratings ranged from 1.6 to 8.0 and arousal ratings ranged from 2.8 to 8.9. The affective spaces of the International Affective Picture System (IAPS)^23^ and Nencki Affective Picture System (NAPS)^24^ are also shown in Fig. 3, for comparison. Figure 4 presents the affective spaces of the e-NatPOEM by image category.

### Classification of the images by valence and arousal

Like seen in other studies, valence and arousal ratings ranged from 1 (minimum) to 9 (maximum)^23,24^. Thus, we categorized the images with the evaluations attributed by the participants according to their combined mean ratings into the four possible quadrants of the model for which the distribution is shown in Table 2.

**Table 2:** Distribution of nature pictures per quadrant by image category.

Category	Quadrant			Total
	1	2	4	
Landscape	26 (96.3%)	1 (3.7%)	–	27
Water	38 (76.0%)	11 (22.0%)	1 (2.0%)	50
Forest/woodland	23 (88.5%)	3 (11.5%)	–	26
Pale Bird	48 (96.0%)	2 (4.0%)	–	50
Colorful Bird	49 (98.0%)	1 (2.0%)	–	50
Sky	49 (98.0%)	1 (2.0%)	–	50
Flower	48 (96.0%)	2 (4.0%)	–	50
Insects	39 (78.0%)	11 (22.0%)	–	50
Sea	42 (84.0%)	8 (16.0%)	–	50
Control	–	2 (6.7%)	28 (93.3%)	28
Overall total	362 (83.6%)	42 (9.7%)	29 (6.7%)	433

According to Table 2, of the 403 pictures (control excluded) that effectively compose e-NatPOEM, 362 (83.6%) were classified by participants in quadrant 1 (low valence and arousal). Pictures with low valence and low arousal would be classified in quadrant 3, but none of these pictures were evaluated as such by participants in our study. A total of 42 (9.7%) pictures are in quadrant 2, being Water, Insects and Sea the dominant categories in this quadrant. The valence and arousal ratings of the pictures are summarized by quadrant in Supplementary Material 3.

### Between-category comparisons

We compared the valence and arousal ratings of each image category with the Landscape category used as a reference, because Landscape pictures are not descriptive of a specific theme and are prevalent in emotional research. The results are shown in Table 3.

**Table 3:** Between-category comparisons of valence and arousal ratings.

Category	Valence		Arousal	
	Percent difference to landscape category	p-value	Percent difference to landscape category	p-value
Water	1.4% (0.0–2.8%)	0.0534	1.3% (− 1.5 to 4.1%)	0.3746
Forest/woodland	− 1.5% (− 2.9 to − 0.1%)	0.0422	1.8% (− 1.0 to 4.6%)	0.2101
Pale Bird	3.1% (1.5–4.7%)	0.0001	− 2.5% (− 6.0 to 1.2%)	0.1799
Colorful Bird	7.9% (6.1–9.6%)	< 0.0001	− 3.0% (− 6.5 to 0.6%)	0.1000
Sky	2.3% (0.9–3.7%)	0.0015	− 11.5% (− 14.4 to − 8.4%)	< 0.0001
Flower	5.4% (4.0–6.8%)	< 0.0001	− 9.3% (− 12.4 to − 6.1%)	< 0.0001
Insects	− 1.1% (− 2.8 to 0.6%)	0.1975	5.1% (1.5–8.8%)	0.0051
Sea	7.0% (5.4–8.6%)	< 0.0001	− 4.5% (− 7.3 to − 1.5%)	0.0031
Control	− 53.6% (− 55.5 to − 51.6%)	< 0.0001	76.5% (69.1–84.4%)	< 0.0001

The mean affective ratings of the Landscape category were 6.7 (6.6–6.8) for valence and 4.2 (4.0–4.3) for arousal.

*P-values by generalized estimated equations.

Valence ratings were significantly higher (p < 0.001) for image categories Pale Bird (+ 3.1%), Colorful Bird (+ 7.9%), Flower (+ 5.4%), and Sea (+ 7.0%). Mean valence ratings were 53.6% lower in control images than in pictures of the Landscape category. In addition, arousal ratings were significantly lower for image categories Sky (− 11.5%) and Flower (− 9.3%) compared to the Landscape category. The mean arousal ratings of control images were 76.5% higher than ratings of the Landscape category.

We also compared the affective ratings by gender separately for each image category and found no significant differences in ratings of valence and arousal between the genders for any image category (Table 4).

**Table 4:** Comparison of valence and arousal ratings between men and women.

Category	Valence		Arousal	
	Percent difference to women	p-value	Percent difference to women	p-value*
Landscape	0.7% (− 3.4 to 5.0%)	0.7416	4.4% (− 4.2 to 13.7%)	0.3294
Water	− 1.2% (− 4.7 to 2.5%)	0.5317	10.2% (1.8–19.3%)	0.0159
Forest/woodland	− 3.3% (− 7.5 to 1.0%)	0.1329	6.0% (− 2.4 to 15.2%)	0.1675
Pale Bird	− 1.5% (− 5.1 to 2.3%)	0.4362	11.0% (2.1–20.8%)	0.0149
Colorful Bird	− 4.9% (− 8.6 to − 1.0%)	0.0151	14.2% (4.5–24.8%)	0.0033
Sky	− 2.8% (− 6.7 to 1.2%)	0.1682	7.2% (− 2.2 to 17.6%)	0.1353
Flower	− 5.9% (− 9.4 to − 2.3%)	0.0016	5.5% (− 3.4 to 15.4%)	0.235
Insects	− 5.9% (− 9.8 to − 1.8%)	0.0056	5.9% (− 1.9 to 14.4%)	0.1442
Sea	− 2.7% (− 5.9 to 0.6%)	0.1117	8.5% (− 0.6 to 18.4%)	0.0677
Control	− 0.6% (− 8.7 to 8.3%)	0.8933	− 0.8% (− 4.4 to 2.9%)	0.6593

*p-values by generalized estimated equations calculated separately for each image category.

### Relationship of valence and arousal with participants’ characteristics

The category of control images is expected to present low valence values, while the remaining dataset categories are expected to present high valence. We examined the scores provided by the participants and this expectative was confirmed, showing that the collected scores are coherent. Thus, we excluded all ratings of control pictures and examined the relationship of valence and arousal with participant characteristics, including age, level of education, income, and degree of contact with nature. All models were adjusted for image category and after excluding control image ratings, the ratings database comprised 24,654 ratings. The analyses showed no significant association between any of the sociodemographic variables investigated and ratings of valence and arousal (Table 5).

**Table 5:** Association of valence and arousal ratings with participant characteristics (n = 24,654).

	Valence		Arousal	
	Mean ratios (95% CI)	p-value	Mean ratios (95% CI)	p-value
**Gender**				
Female				
Male	0.97 (0.94–1.0)	0.0253	1.07 (1.01–1.14)	0.0289
Age (10 years)	1.01 (1.0–1.02)	0.0553	1.0 (0.98–1.02)	0.8069
**Monthly income**^**a**^				
≤ US$ 176.00	1.01 (0.95–1.08)	0.6955	0.96 (0.85–1.08)	0.4968
US$ 176.01–880.00	1.03 (0.96–1.1)	0.4155	0.99 (0.87–1.12)	0.8358
US$ 880.01–1760.00	.98 (0.92–1.05)	0.6487	0.96 (0.84–1.1)	0.5632
> US$ 1760.00	1.02 (0.95–1.09)	0.597	0.93 (0.83–1.06)	0.2858
**Contact with nature**				
Never				
Rarely	1.01 (0.94–1.08)	0.8408	1.01 (0.81–1.24)	0.9545
Infrequently	1.0 (0.94–1.07)	0.8963	1.0 (0.82–1.21)	0.9753
Frequently	1.0 (0.94–1.07)	0.9002	0.98 (0.8–1.19)	0.8338
Very frequently	0.98 (0.91–1.04)	0.4906	1.04 (0.86–1.27)	0.6804
**Education level**				
Illiterate or primary school				
Secondary school	0.96 (0.91–1.02)	0.1538	0.88 (0.77–1.01)	0.0634
University undergraduate	0.96 (0.92–1.01)	0.1273	0.9 (0.8–1.02)	0.0907
University postgraduate	0.99 (0.94–1.04)	0.7881	0.92 (0.81–1.04)	0.1851
Master’s degree	0.96 (0.91–1.01)	0.1451	0.94 (0.83–1.08)	0.3895
Doctorate degree	0.98 (0.92–1.05)	0.5574	0.99 (0.84–1.17)	0.8852

^a^Approximate conversion from Brazilian Real to American Dollar on May 20, 2020.

### Validation—image attributes

To categorize the images, in addition to the quantitative assessment of pleasure (unhappy/happy) and arousal (relaxed/excited) ratings, we conducted a qualitative assessment of the images with participants describing, in a single, freely written word, what the picture conveyed while viewing it. This qualitative description was used to group the pictures into the attribute groups shown in Table 6.

**Table 6:** Classification of nature pictures by attribute groups.

Image category by attribute	Picture number	Category
Beauty	4, 23, 31, 57, 58, 59, 60, 63, 67, 71, 73, 74, 95, 113, 114, 121, 126, 133, 144, 148, 150, 151, 152, 157, 158, 160, 161, 163, 166, 167, 168, 170, 171, 172, 173, 176, 177, 178, 179, 180, 182, 184, 185, 186, 187, 188, 189, 190, 191, 192, 193, 194, 197, 199, 202, 209, 226, 234, 236, 247, 248, 251, 253, 254, 255, 257, 258, 260, 261, 262, 263, 265, 266, 267, 268, 269, 270, 271, 274, 276, 277, 278, 279, 280, 281, 282, 283, 284, 285, 287, 291, 292, 294, 296, 297, 299, 300, 301, 302, 303, 307, 308, 309, 310, 311, 312, 314, 316, 317, 318, 319, 320, 322, 323, 324, 325, 326, 328, 329, 330, 333, 334, 335, 338, 344, 347, 349, 353, 358, 365, 369, 371, 374, and 406	Total = 134 images Flower (36), Colorful Bird (33), Insects (30), forest/woodland (10), Pale Bird (8), Sky (7), Sea (6), water (3), Landscape (1), control (0)
Peace/tranquility	1, 5, 9, 12, 13, 14, 16, 17, 19, 21, 22, 24, 27, 30, 32, 34, 36, 37, 38, 39, 40, 41, 43, 46, 47, 48, 49, 50, 52, 53, 54, 55, 56, 57, 64, 66, 68, 69, 70, 72, 74, 75, 76, 77, 80, 81, 83, 85, 86, 87, 88, 89, 90, 91, 92, 94, 96, 97, 98, 99, 100, 108, 109, 110, 116, 117, 118, 123, 124, 125, 132, 137, 146, 193, 201, 202, 203, 204, 205, 206, 207, 208, 209, 211, 212, 213, 214, 215, 217, 218, 219, 220, 221, 223, 224, 225, 227, 228, 229, 230, 231, 232, 235, 237, 238, 239, 240, 241, 243, 244, 245, 246, 247, 250, 272, 273, 274, 282, 288, 290, 291, 319, 321, 352, 354, 355, 358, 360, 361, 362, 364, 367, 372, 375, 377, 378, 379, 380, 381, 382, 383, 384, 385, 387, 388, 389, 390, 391, 392, 393, 394, 395, 396, 397, 398, 399, 400, 406, 443, 447, and 448	Total = 161 images Sky (40), Sea (34), forest/woodland (33), water (28), Pale Bird (12), Flower (7), Landscape (4), Insects (2), Colorful Bird (1), control (0)
Positive states	2, 3, 15, 20, 28, 42, 51, 101, 103, 104, 105, 106, 107, 111, 112, 113, 115, 119, 122, 127, 128, 129, 131, 132, 134, 135, 138, 140, 141, 143, 144, 145, 149, 150, 153, 154, 155, 156, 158, 159, 162, 164, 165, 167, 169, 170, 174, 175, 181, 183, 191, 192, 193, 195, 196, 200, 201, 242, 252, 256, 259, 262, 264, 275, 286, 289, 296, 315, 327, 332, 338, 343, 344, 348, 350, 351, 386, and 480	Total = 78 images Pale Bird (27), Colorful Bird (22), Flower (9), Insects (8), water (6), Sea (2), forest/woodland (1), Sky (2), control (1), Landscape (0)
Miscellaneous	7, 8, 10, 18, 21, 26, 29, 33, 35, 40, 44, 45, 62, 65, 74, 78, 79, 82, 84, 93, 96, 100, 102, 120, 130, 131, 132, 139, 142, 147, 185, 192, 198, 209, 210, 213, 216, 222, 233, 239, 249, 288, 289, 292, 293, 295, 303, 304, 305, 306, 312, 313, 331, 335, 337, 339, 342, 345, 363, 368, 370, 373, 376, 448, 455, 471, and 479	Total = 67 images Water (12), Insects (12), forest/woodland (10), Pale Bird (8), Sky (8), Flower (5), Sea (5), Colorful Bird (3), control (3), Landscape (1)
Negative states	6, 11, 25, 136, 298, 336, 340, 341, 346, 356, 357, 359, 366, 451, 452, 453, 454, 456, 457, 458, 459, 460, 461, 462, 463, 464, 465, 466, 467, 468, 469, 470, 472, 473, 474, 475, 476, 477, and 478	Total = 39 images Control (26), Sea (4), Insects (4), water (3), Pale Bird (1), Flower (1)

Picture categories: Water, 1–50; Forest/Woodland, 51–100; Pale Bird, 101–150; Colorful Bird, 151–200; Sky, 201–250; Flower, 251–300; Insects, 301–350; Sea, 351–400; Landscape, 406–448; and Control, 451–480.

The pictures were grouped into five attribute groups, namely (1) Beauty, (2) Peace/tranquility, (3) Positive states, (4) Miscellaneous, and (5) Negative states according to the emotions that each picture evoked, as shown in Table 6. There are pictures with numbers greater than 433 (403 + 28 control images) because e-NatPOEM originated from a larger set of image files named with sequential numbers; these were curated by independent photographers (more details in the section "Methods").

Beauty is not an emotion per se but is associated with the aesthetic experience^47^. It is considered a fundamental aspect of the human being and resides in the characteristics of the object and in the interaction between the object and the person's cognitive and affective processes^48^. Thus, it was included in the attributes set.

The Peace/tranquility attribute group was the largest one, comprising 161 pictures mostly of Sky (40), Sea (34), Forest/Woodland (33), and Water (28) with the colors blue and green being the predominant color.

The Beauty attribute group comprised 134 pictures, mostly of Flower (36), Colorful Bird (33), and Insects (30), which are characterized by more dynamic photographs with more vibrant colors. In the Positive states attribute group comprising 78 pictures, the more prevalent image categories were Pale Bird (27) and Colorful Bird (22). The Miscellaneous attribute group comprised 67 pictures, mostly of Water (12), Insects (12), and Forest/Woodland (10), and it also included pictures of other image categories as its name implies. Lastly, in the Negative states attribute group, comprising 39 pictures, the most prevalent categories were Control (26), Sea (4), and Insects (4).

The 433 pictures of the original database were classified into these five attribute groups. Some pictures (36) tied for two attributes, i.e., they were classified into two attribute groups. These pictures were mostly of Flower (8), Colorful Bird (5), and Sky (5) and have the potential to produce double feelings. In addition, some pictures (5), most of which of Colorful Bird (2), tied for three attributes, i.e., they were included in three attribute groups. The following pictures were included in two attribute groups: 21, 40, 57, 96, 100, 113, 131, 144, 150, 158, 167, 170, 185, 191, 201, 202, 213, 239, 247, 262, 274, 282, 288, 289, 291, 292, 296, 303, 312, 319, 335, 338, 344, 358, 406, and 448. The pictures that tied for three attribute groups were 74, 132, 192, 193, and 209.

Table 7 shows the predominant words by attribute group mentioned by participants, totaling 4702 mentions. The Peace/tranquility group had the largest number of mentions (1844, 39.18% of all mentions), of which the word “peace” accounted for the largest number of mentions (1166).

**Table 7:** Distribution of mentions according to the predominant words assigned by participants.

Image category by attribute	Predominant word	N	%
Beauty	Beauteous	1003	21.33
	Beautiful	53	1.12
	Perfection	18	0.38
	Wonderful	4	0.08
Peace/tranquility	Peace	1166	24.79
	Tranquility	523	11.12
	Calm	126	2.67
	Relaxation	13	0.27
	Quietness	12	0.25
	Serenity	4	0.08
Positive states	Freedom	324	6.89
	Life	111	2.36
	Love	78	1.65
	Joy	66	1.4
	Companionship	31	0.65
	Balance	20	0.42
	Lightness	17	0.36
	Hope	13	0.27
	Happiness	9	0.19
	Unity	9	0.19
	Partnership	8	0.17
	Affection	5	0.1
Miscellaneous	Curiosity	52	1.1
	Immensity	45	0.95
	Force	35	0.74
	Family	28	0.59
	Warmth	26	0.55
	Agitation	24	0.51
	End	23	0.48
	Delicacy	22	0.46
	Adventure	17	0.36
	Work	13	0.27
	Weird	12	0.25
	Challenge	10	0.21
	Nothing	10	0.21
	Others^a^	121	2.57
Negative states	Sadness	349	7.42
	Fear	193	4.1
	Loneliness	26	0.55
	Dirt	25	0.53
	Chaos	21	0.44
	Death	17	0.36
	Disgust	11	0.23
	Abandonment	9	0.19
Total	–	4702	100.0

^a^Others: arousal (7), light (7), autumn (7), speed (7), attention (6), path (6), nature (6), observation (6), rest (5), freshness (5), cold (5), expanse (5), wind (5), aridity (4), fluidity (4), mystery (4), precision (4), drought (4), survival (4), sleep (4), transformation (4), admiration (3), care (3), power (3), and storm (3).

The Beauty attribute group had the second largest number of mentions (1078, 22.91% of all mentions) and the words “beauteous” and “beautiful” contributed the most mentions (1003) to this attribute group.

The Positive states attribute group had the third largest number of mentions (691, 14.65% of all mentions) and the word “freedom” was mentioned 324 times.

The Negative states attribute group had 651 mentions (13.82% of all mentions) and the word “sadness” contributed with 349 mentions.

Lastly, the Miscellaneous attribute group received 438 mentions (9.25% of all mentions) and the word “curiosity” was mentioned 52 times. Because several words were mentioned in this group, we created the item “others” for words mentioned fewer than ten times.

Eleven pictures were described by participants as having negative attributes and were grouped into the Negative states group. The pictures were mostly of Sea (4), water (3), and Insects (2) and had high valence and low arousal, but in the qualitative description (the word describing what the picture conveyed) were described as potentially producing negative feelings. The pictures for which discrepancies in valence and arousal ratings were observed and image attributes assigned by participants are shown in Supplementary Material 4.

Pictures of image categories Sea (4) and water (3) included in the Negative states group were images of rough waters or seas with predominantly darker colors, which evoke something dark or mysterious. In addition, the pictures of the image category Insects (2) included in this group evoked both the feeling of Insects “fear” and of “loneliness” in the picture of a butterfly alone (image 340).

## Discussion

In this study, we presented the e-Nature Positive Emotions Photography Database (e-NatPOEM) comprising 403 high-resolution photographs grouped into nine general categories: Landscape, Water, Forest/woodland, Pale Bird, Colorful Bird, Sky, Flower, Insects, and Sea. The images were also grouped into five groups based on a single word that described what the picture conveyed when participants looked at them: Beauty (aesthetic experience), Peace/tranquility, Positive states, Miscellaneous, and Negative states.

The affective space of e-NatPOEM pictures differs from the boomerang shape of the IAPS^23^ and the more linear distribution of scores in the affective space of the NAPS^24^, with the pictures clustered in the first quadrant of high valence and low arousal, which is associated with states of greater relaxation and pleasure, what promotes positive emotions in according to the definition of positive emotions adopted in the present study.

The NAPS database comprises 1376 photographs rated by 204 mostly European participants and grouped into five categories: Faces, People, Animals, Objects, and Landscape.

In the IAPS^49^ and NAPS^24^ databases, correlations between valence and arousal were stronger in women than in men for pictures of people, faces, and objects in NAPS and unpleasant pictures in IAPS. For nature images, we did not find any gender difference either, which reveals a more universal character and expands the many uses of these images.

We also found no significant differences in valence and arousal ratings for the other participant characteristics, including age, education, and income.

In our study, we used two continuous sliders that enabled participants to attribute ratings for the affective dimensions of valence and arousal on a 9-point scale termed Affective Slider (AS)^39^ and also investigated how participants described in a single word what feeling the pictures evoked.

Of the four main existing picture databases (GAPED, EmoPics, NAPS, IAPS), the NAPS database is similar to the e-NatPOEM in the use of sliding scales and high-quality, realistic pictures^24^. However, it should be noted that the pictures may differ both quantitatively and qualitatively because our selection criteria include some photographic parameters that were not used by the other picture databases such as the composition of the image for its significant influence on the aesthetic experience.

Researchers argue that there is a certain connection between human aesthetic experience and the sensation caused by visual stimuli regardless of source, culture, and experience^50^. The aesthetics in photography deals with the creation and enhancement of beauty in images.

Even though human responses to Landscape have been widely investigated^51–53^, in our study we showed that a specific element in a photograph can stand out from the Landscape and trigger different aesthetic reactions and levels of relaxation. Pictures of Sea, Colorful Bird, Flower, and Pale Bird had high valence, whereas pictures of Flower and Sky were the most relaxing.

In the NAPS, the Landscape category comprising 185 photographs combines the Flower, Sea, Water, and Plants categories^24^. In our study, Landscape was defined as scenes depicting a wide range of natural elements and we also investigated some of these elements separately as standalone image categories.

In our study, mean valence ratings for image categories Sea (7.2), Colorful Bird (7.2), Flower (7.0), and Pale Bird (6.9) were significantly higher than for Landscape pictures (6.7). The NAPS contains only six pictures of Sea and 12 pictures of Flower with mean valence ratings of 7.5 and 7.3, respectively^24^. In our study, pictures of bird were found to promote positive states as much as pictures of Sea, but the NAPS database contains only seven pictures of bird, five of which with low valence and showing either dead or sick bird, in addition to some pelicans, ducks, and pigeons, whose mean valence was 6.8^24^, closer to what we observed for Pale Bird in our study.

Pictures of Sky (3.7) and Flower (3.8) were the most relaxing (low arousal) among all image categories analyzed in our study. In the Landscape category of the NAPS database, mean arousal ratings of the 12 pictures of Flower and two pictures of Sky were 3.4 and 2.8, respectively^24^.

By also analyzing the image attributes as described by participants in a single word, we observed that in addition to valence and arousal, the pictures also reflect emotions, feelings, or symbolic representations that enabled us to qualitatively classify the e-NatPOEM pictures. Thus, the pictures were categorized into four groups: (1) Beauty, (2) Peace/tranquility, (3) Positive emotions (joy, happiness), and (4) a group comprising miscellaneous pictures. The dimensions of valence and arousal indicate how unhappy/happy and relaxing/excited an experience may be, but the words have given a symbolic meaning to the experience.

The words used by the participants in this study represent a range of emotions that characterize the aesthetic experience. The literature classifies these emotions as epistemic aesthetic (e.g., interest), prototypical (e.g., beautiful), fun (joy), relax or alert effects, or even unpleasant emotions that generate a corresponding negative aesthetic perception^54^, such as those observed in our control images (for example, ugliness and degradation of nature). Researchers have shown that involvement with the beauty of nature moderates subjective well-being and increases the connection with nature^55^. The directed attention to elements of nature increases the trait of appreciation of beauty^56^, which can increasingly result in positive emotions. Therefore, making e-NatPOEM database available in hospital settings, for example, could be very useful and deserves further consideration.

The feeling of beauty increases linearly with pleasure, and strong pleasure is always beautiful and produced reliably by beautiful stimuli^57^, hence the importance of an adequate selection of visual stimuli when aiming to provide positive aesthetic experiences. The feeling of beauty and the amplitude of pleasure are independent of stimulus duration, which can be very short, but vary according to stimulus type and judgment of beauty^58^. These findings reinforce the notion that exposure to the beauty of nature and its elements, albeit indirectly, promote positive emotions and provide instant subjective restoration, which was the main goal in developing and validating the e-NatPOEM database.

### Study limitations

Previous individual experiences were not considered as they would not fit into the study design, but because they can influence the analysis of any visual stimulus, should be taken into account even when rating a database of pictures that promote positive emotions.

The cubs, puppies, and young animal categories were not investigated, but it could be included in the database in the future, because cubs of wild animals have been shown to produce positive feelings^59^.

The e-NatPOEM is a relatively small database comprising 403 photographs of nature with different semantic contents, but it is the only database dedicated to positive, beautiful, relaxing and freely accessible pictures and having its therapeutic potential confirmed for clinical use. Expanding the database with new categories and images from geographic locations other than imagens from neotropical regions, and the evaluation of the e-NatPOEM with groups of participants from different countries deserve future research.

The e-NatPOEM is being tested for clinical use in patients receiving chemotherapy.

## Supplementary Information

Supplementary Information 1.

Supplementary Information 2.

Supplementary Information 3.

Supplementary Information 4.

## Data Availability

The datasets generated during and/or analyzed during the current study are available upon request from the corresponding author.
