# Supplementary material for: e-Nature Positive Emotions Photography Database (e-NatPOEM): affectively rated nature images promoting positive emotions
Source: Sci Rep. 2021 Jun 3;11:11696. doi: 10.1038/s41598-021-91013-9 (PMC8175760; doi:10.1038/s41598-021-91013-9)
Supplement: Supplementary file 1 — Supplementary Information 1. [file 41598_2021_91013_MOESM1_ESM.pdf]

Supplementary Material 1. General identification of pictures of control group.

e-Nature Positive Emotions Photography Database (e-NatPOEM) - affectively rated nature images promoting positive emotions

Daniela Dal Fabbro, Giulia Catissi, Gustavo Borba, Luciano Lima, Erika Hingst-Zaher, João Rosa, Elivane Victor, Leticia Bernardes, Tinely Souza, Eliseth Leão.

Copyright photos

Photo ID: 455<br>Valence mean (95%CI): 4.8 (4.4; 5.2)<br>Arousal mean (95%CI): 5.0 (4.6; 5.5)<br>Description: a dead, dried flower with fallen petals
Photo ID: 456<br>Valence mean (95%CI): 3.2 (2.8; 3.6)<br>Arousal mean (95%CI): 5.7 (5.2; 6.3)<br>Description: a cluster of dead and dried flowers with fallen petals
Photo ID: 459<br>Valence mean (95%CI): 2.6 (2.2; 3.0)<br>Arousal mean (95%CI): 7.6 (6.9; 8.4)<br>Description: dead white chickens
Photo ID: 460<br>Valence mean (95%CI): 4.3 (3.9; 4.8)<br>Arousal mean (95%CI): 8.2 (7.7; 8.8)<br>Description: a hairy spider in the sand
Photo ID: 461<br>Valence mean (95%CI): 3.9 (3.5; 4.3)<br>Arousal mean (95%CI): 7.2 (6.6; 7.9)<br>Description: a sky with dark storm clouds
Photo ID: 464<br>Valence mean (95%CI): 2.0 (1.7; 2.3)<br>Arousal mean (95%CI): 8.6 (7.9; 9.0)<br>Description: a tsunami invading and destroying the city
Photo ID: 465<br>Valence mean (95%CI): 3.6 (3.2; 4.0)<br>Arousal mean (95%CI): 5.4 (4.9; 5.9)<br>Description: a dried rose flower on a white background
Photo ID: 466<br>Valence mean (95%CI): 2.2 (1.9; 2.5)<br>Arousal mean (95%CI): 8.1 (7.4; 8.8)<br>Description: a lot of garbage dumped in a green area
Photo ID: 467<br>Valence mean (95%CI): 2.9 (2.4; 3.3)<br>Arousal mean (95%CI): 7.5 (6.9; 8.1)<br>Description: a polluted open-air sewer with nature all around
Photo ID: 468<br>Valence mean (95%CI): 2.0 (1.7; 2.4)<br>Arousal mean (95%CI): 8.9 (8.3; 9.0)<br>Description: several trees cut down on a big city street with a bus passing by
Photo ID: 469<br>Valence mean (95%CI): 2.6 (2.3; 3.0)<br>Arousal mean (95%CI): 7.4 (6.8; 8.0)

Description: the whole of the remains of a cut tree
Photo ID: 470<br>Valence mean (95%CI): 3.2 (2.8; 3.6)<br>Arousal mean (95%CI): 7.4 (6.8; 8.0)<br>Description: a white chicken with its beak cut off
Photo ID: 471<br>Valence mean (95%CI): 3.9 (3.5; 4.4)<br>Arousal mean (95%CI): 7.2 (6.6; 7.9)<br>Description: aerial image of a big city without nature
Photo ID: 472<br>Valence mean (95%CI): 2.4 (2.0; 2.8)<br>Arousal mean (95%CI): 7.5 (6.6; 7.9)<br>Description: extremely dry soil, with cracks and dead animals
Photo ID: 473<br>Valence mean (95%CI): 2.5 (2.1; 2.9)<br>Arousal mean (95%CI): 8.2 (7.5; 8.9)<br>Description: car traffic jam of a big city
Photo ID: 474<br>Valence mean (95%CI): 3.2 (2.9; 3.6)<br>Arousal mean (95%CI): 8.1 (7.5; 8.8)<br>Description: two snakes intertwined, the yellow snake biting the black snake
Photo ID: 475<br>Valence mean (95%CI): 3.7 (3.3; 4.1)<br>Arousal mean (95%CI): 5.5 (5.0; 6.1)<br>Description: dry and dead dark pink flower
Photo ID: 477<br>Valence mean (95%CI): 3.2 (2.8; 3.7)<br>Arousal mean (95%CI): 7.9 (7.2; 8.6)<br>Description: polluted water with bubbles
Photo ID: 478<br>Valence mean (95%CI): 3.6 (3.2; 4.1)<br>Arousal mean (95%CI): 8.1 (7.5; 8.7)<br>Description: snake coming out of a hole with its mouth open for attack
Photo ID: 451<br>Valence mean (95%CI): 2.2 (1.9; 2.6)<br>Arousal mean (95%CI): 8.2 (7.6; 8.9)<br>Description: Tenement in a densely populated and degraded.
Photo ID: 452<br>Valence mean (95%CI): 2.0 (1.7; 2.3)<br>Arousal mean (95%CI): 8.3 (7.3; 8.5)<br>Description: photo of environmental pollution with an empty soda bottle discarded on the floor next to a dead butterfly
Photo ID: 453<br>Valence mean (95%CI): 2.7 2.3; 3.2)<br>Arousal mean (95%CI): 7.9 (7.3; 8.5)<br>Description: lots of garbage floating in the blue ocean
Photo ID: 454<br>Valence mean (95%CI): 2.5 (2.2; 2.9)<br>Arousal mean (95%CI): 7.4 (6.8; 8.1)<br>Description: two trees closed and fallen
Photo ID: 457<br>Valence mean (95%CI): 2.1 (1.8; 2.5)<br>Arousal mean (95%CI): 7.6 (7.0; 8.3)<br>Description: owl hit by road
Photo ID: 458<br>Valence mean (95%CI): 3.5 (3.1; 3.9)

Arousal mean (95%CI): 6.2 (5.7; 6.9)<br>Description: polluted river with vegetation around
Photo ID: 462<br>Valence mean (95%CI): 2.2 (1.9; 2.5)<br>Arousal mean (95%CI): 8.1 (7.5; 8.7)<br>Description: deforested vegetation
Photo ID: 463<br>Valence mean (95%CI): 2.0 (1.7; 2.4)<br>Arousal mean (95%CI): 7.8 (7.1; 8.5)<br>Description: cut tree trunk
Photo ID: 476<br>Valence mean (95%CI): 1.6 (1.4; 1.8)<br>Arousal mean (95%CI): 8.5 (7.9; 9.0)<br>Description: dead birds
