# Supplementary material for: e-Nature Positive Emotions Photography Database (e-NatPOEM): affectively rated nature images promoting positive emotions
Source: Sci Rep. 2021 Jun 3;11:11696. doi: 10.1038/s41598-021-91013-9 (PMC8175760; doi:10.1038/s41598-021-91013-9)
Supplement: Supplementary file 3 — Supplementary Information 3. [file 41598_2021_91013_MOESM3_ESM.pdf]

Supplementary Material 3. General identification of pictures with valence and arousal by quadrant.

e-Nature Positive Emotions Photography Database (e-NatPOEM) - affectively rated nature images promoting positive emotions

Daniela Dal Fabbro, Giulia Catissi, Gustavo Borba, Luciano Lima, Erika Hingst-Zaher, João Rosa, Elivane Victor, Leticia Oliveira, Tinely Souza, Eliseth Leão.

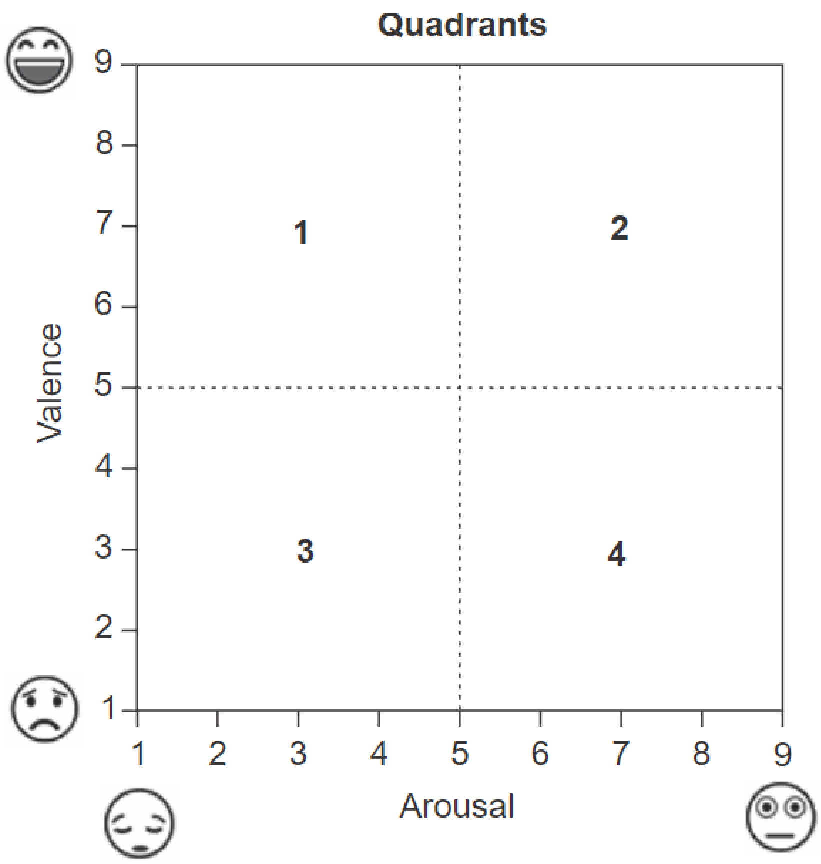

QUADRANT 1

Photo ID:395
Valence mean (95% CI): 7.9 (7.6; 8.2)
Arousal mean (95% CI): 2.9 (2.5; 3.4)

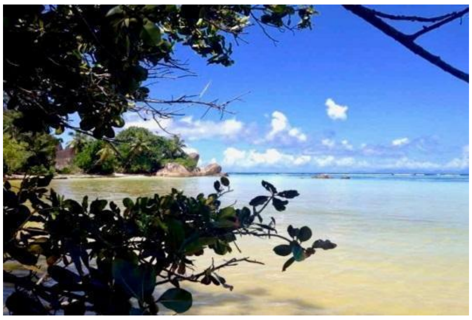

Photo ID:390
Valence mean (95% CI): 8.0 (7.7; 8.4)
Arousal mean (95% CI): 3.1 (2.6; 3.7)

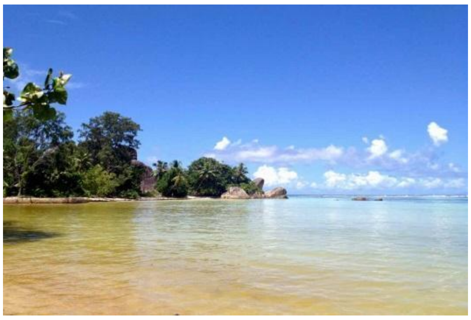

Photo ID:385
Valence mean (95% CI): 7.5 (7.2; 7.8)
Arousal mean (95% CI): 2.8 (2.4; 3.3)

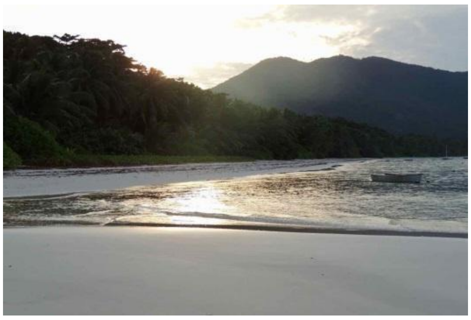

Photo ID:386

Valence mean (95% CI): 8.0 (7.6; 8.3)
Arousal mean (95% CI): 3.3 (2.8; 3.8)

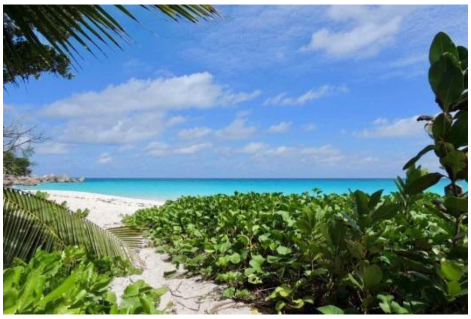

Photo ID:388
Valence mean (95% CI): 7.9 (7.5; 8.2)
Arousal mean (95% CI): 3.2 (2.8; 3.7)

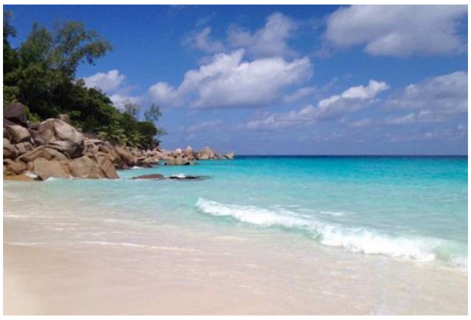

Photo ID:223
Valence mean (95% CI): 7.5 (7.2; 7.8)
Arousal mean (95% CI): 2.9 (2.5; 3.4)

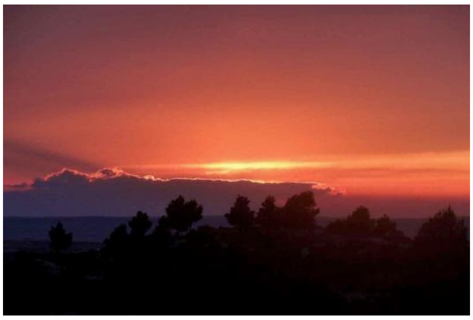

Photo ID:241
Valence mean (95% CI): 7.7 (7.4; 8.0)

Arousal mean (95% CI): 3.1 (2.7; 3.6)

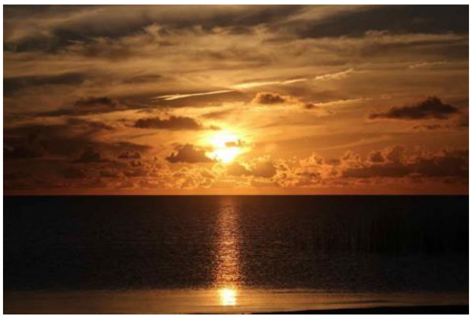

Photo ID:399
Valence mean (95% CI): 7.7 (7.3; 8.0)
Arousal mean (95% CI): 3.1 (2.6; 3.6)

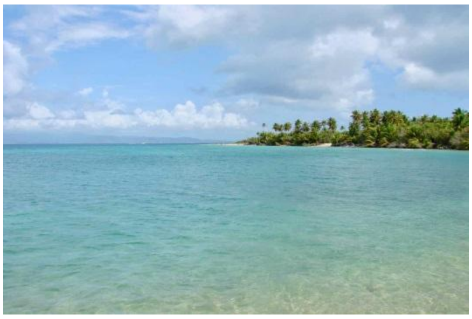

Photo ID:360
Valence mean (95% CI): 7.3 (6.9; 7.7)
Arousal mean (95% CI): 2.8 (2.5; 3.2)

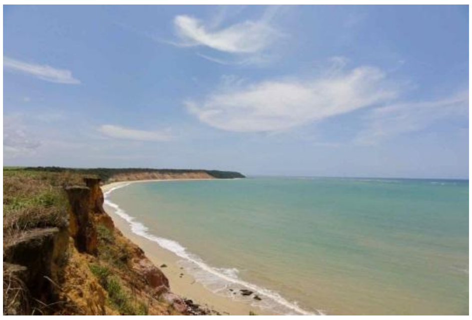

Photo ID:381
Valence mean (95% CI): 7.5 (7.2; 7.9)
Arousal mean (95% CI): 3.0 (2.7; 3.4)

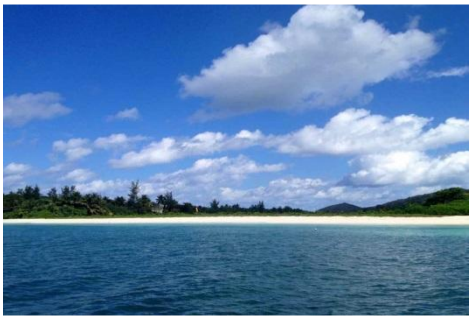

Photo ID:287
Valence mean (95% CI): 7.6 (7.3; 7.9)

Arousal mean (95% CI): 3.2 (2.8; 3.7)

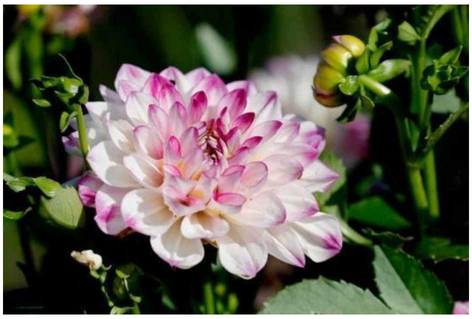

Photo ID:100
Valence mean (95% CI): 7.5 (7.2; 7.7)
Arousal mean (95% CI): 3.1 (2.8; 3.5)

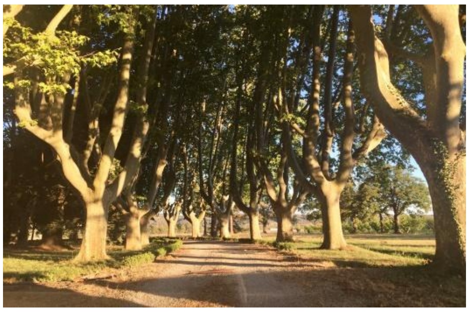

Photo ID:290
Valence mean (95% CI): 7.5 (7.2; 7.7)
Arousal mean (95% CI): 3.1 (2.7; 3.5)

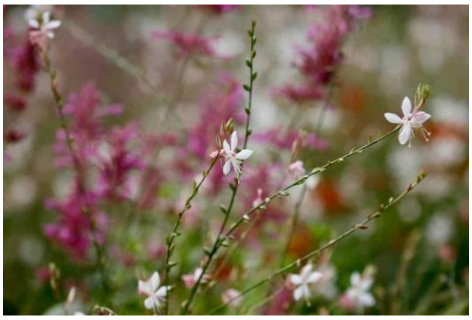

Photo ID:372
Valence mean (95% CI): 7.9 (7.6; 8.3)
Arousal mean (95% CI): 3.5 (3.0; 4.2)

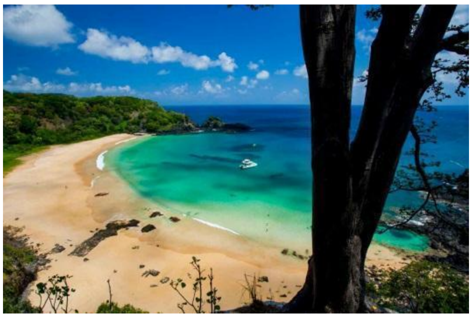

Photo ID:393
Valence mean (95% CI): 7.7 (7.0; 7.8)

Arousal mean (95% CI): 3.3 (2.8; 3.6)

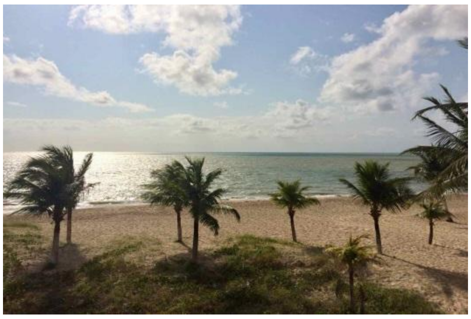

PHOTO ID: 160
Valence mean (95% CI): 7.6 (7.3; 7.8)
Arousal mean (95% CI): 3.3 (2.9; 3.7)

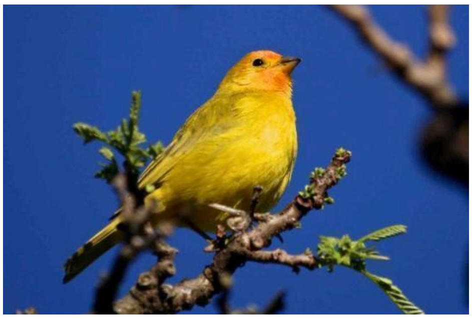

PHOTO ID: 276
Valence mean (95% CI): 7.5 (7.2; 7.9)
Arousal mean (95% CI): 3.2 (2.8; 3.5)

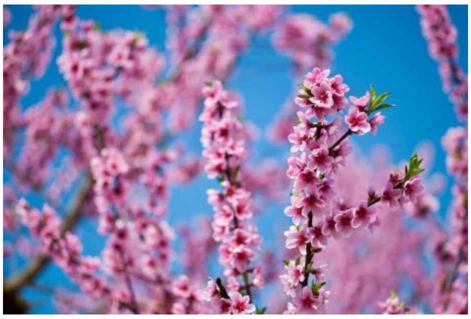

PHOTO ID: 400
Valence mean (95% CI): 7.5 (7.1; 7.9)
Arousal mean (95% CI): 3.2 (2.7; 3.7)

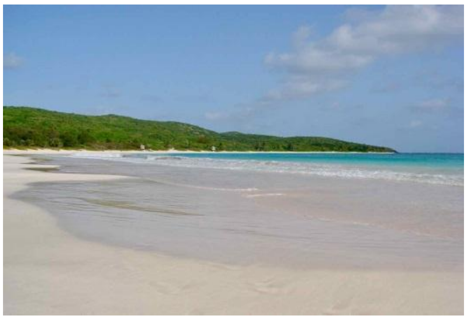

Photo ID:266
Valence mean (95% CI): 7.7 (7.4; 8.0)

Arousal mean (95% CI): 3.4 (3.0; 4.0)

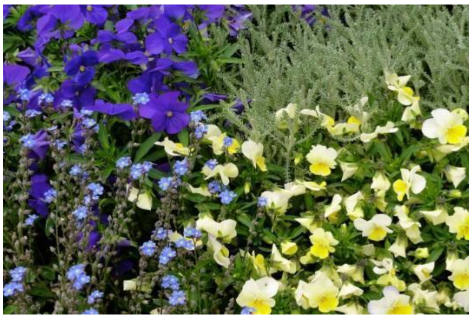

PHOTO ID: 53
Valence mean (95% CI): 7.1 (6.8; 7.4)
Arousal mean (95% CI): 2.9 (2.6; 3.3)

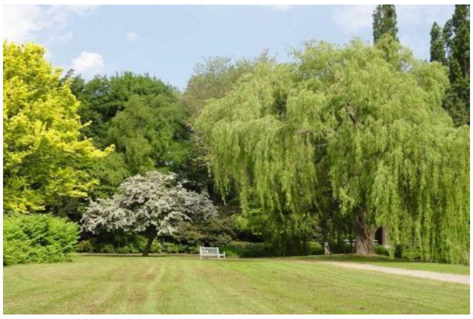

PHOTO ID: 111
Valence mean (95% CI): 7.6 (7.2; 7.9)
Arousal mean (95% CI): 3.4 (2.9; 4.0)

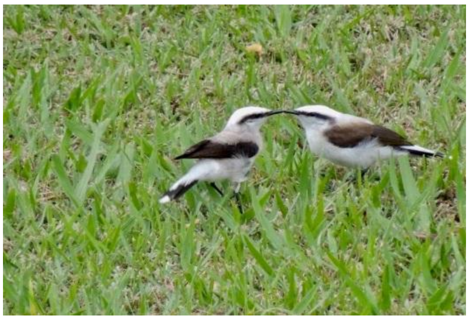

PHOTO ID: 272
Valence mean (95% CI): 7.3 (6.9; 7.8)
Arousal mean (95% CI): 3.1 (2.6; 3.5)

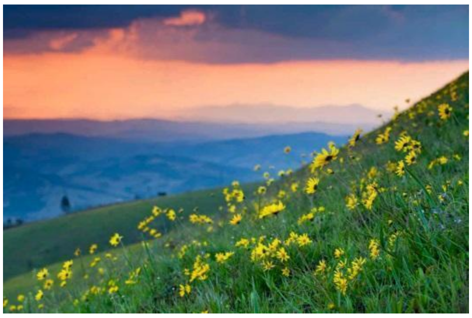

PHOTO ID: 122
Valence mean (95% CI): 7.5 (7.2; 7.8)

Arousal mean (95% CI): 3.3 (2.9; 3.8)

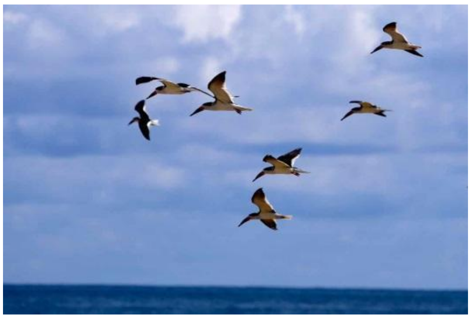

PHOTO ID: 205
Valence mean (95% CI): 7.4 (7.0; 7.7)
Arousal mean (95% CI): 3.2 (2.8; 3.6)

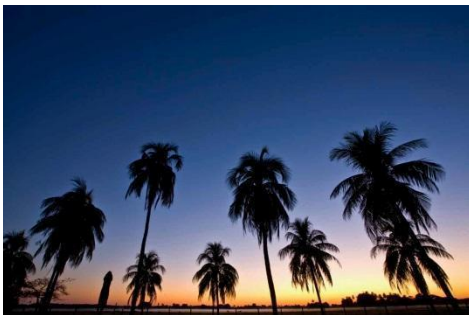

PHOTO ID: 378
Valence mean (95% CI): 7.7 (6.8; 7.4)
Arousal mean (95% CI): 3.5 (3.7; 4.6)

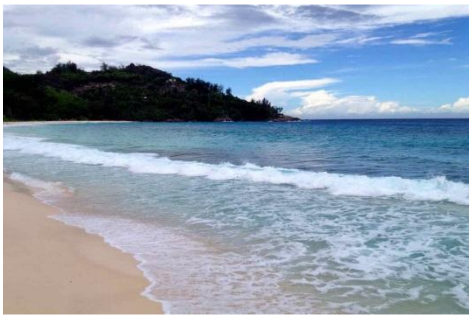

PHOTO ID: 383
Valence mean (95% CI): 7.4 (6.6; 7.4)
Arousal mean (95% CI): 3.2 (2.9; 3.7)

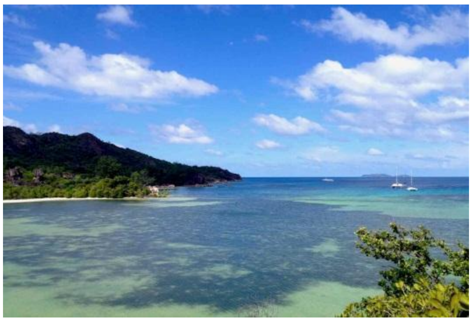

PHOTO ID: 9
Valence mean (95% CI): 7.6 (7.3; 7.9)

Arousal mean (95% CI): 3.5 (3.0; 4.0)

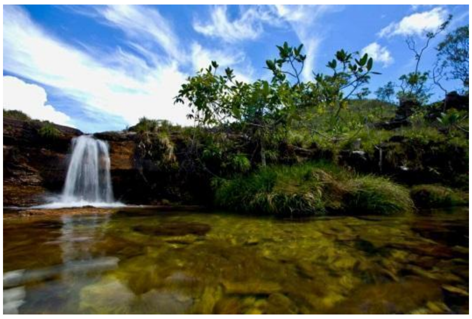

PHOTO ID: 30
Valence mean (95% CI): 7.3 (6.9; 7.8)
Arousal mean (95% CI): 3.2 (2.7; 3.7)

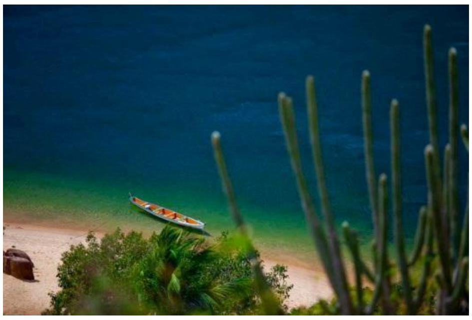

PHOTO ID: 88
Valence mean (95% CI): 7.1 (6.8; 7.4)
Arousal mean (95% CI): 3.0 (2.7; 3.3)

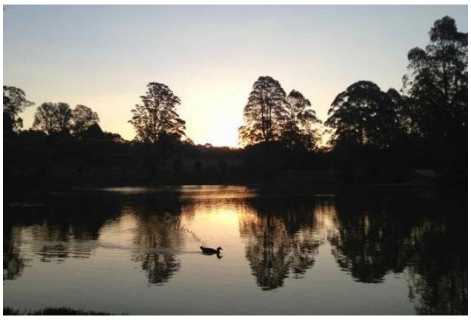

PHOTO ID: 274
Valence mean (95% CI): 7.6 (7.3; 7.9)
Arousal mean (95% CI): 3.5 (3.0; 4.0)

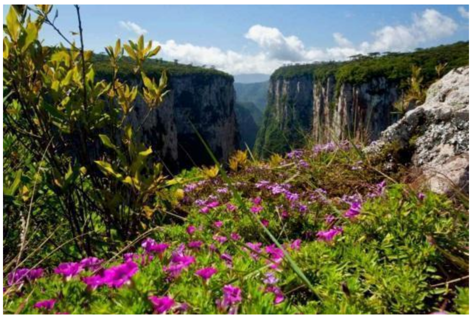

PHOTO ID: 361
Valence mean (95% CI): 7.3 (6.9; 7.8)

Arousal mean (95% CI): 3.2 (2.8; 3.7)

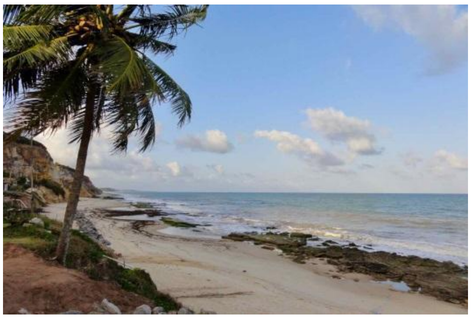

PHOTO ID: 367
Valence mean (95% CI): 7.3 (7.0; 7.6)
Arousal mean (95% CI): 3.2 (2.8; 3.7)

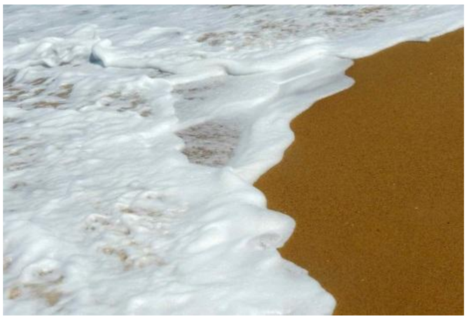

PHOTO ID: 369
Valence mean (95% CI): 7.6 (7.3; 7.9)
Arousal mean (95% CI): 3.5 (3.1; 4.0)

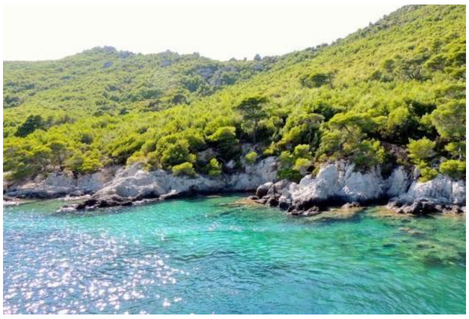

PHOTO ID: 43
Valence mean (95% CI): 7.2 (6.8; 7.6)
Arousal mean (95% CI): 3.1 (2.7; 3.7)

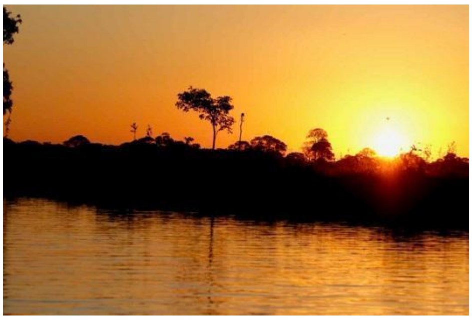

PHOTO ID: 234
Valence mean (95% CI): 7.2 (6.8; 7.6)

Arousal mean (95% CI): 3.1 (2.7; 3.6)

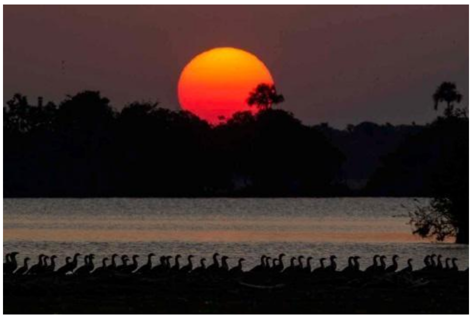

PHOTO ID: 282
Valence mean (95% CI): 7.2 (6.8; 7.6)
Arousal mean (95% CI): 3.1 (2.7; 3.5)

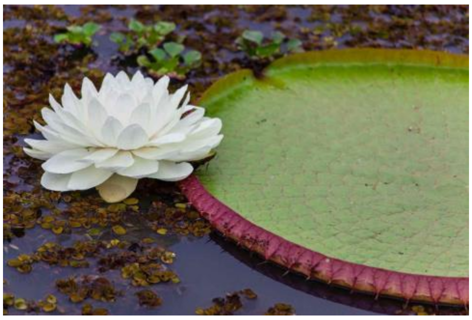

PHOTO ID: 382
Valence mean (95% CI): 7.5 (7.1; 7.8)
Arousal mean (95% CI): 3.4 (2.9; 3.9)

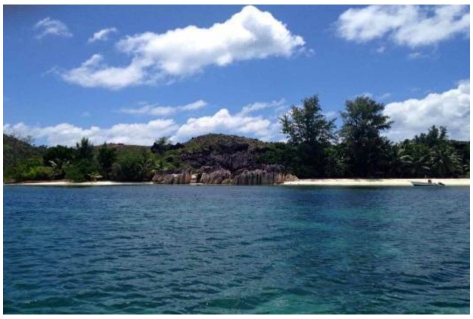

PHOTO ID: 318
Valence mean (95% CI): 7.6 (7.3; 7.9)
Arousal mean (95% CI): 3.6 (3.1; 4.1)

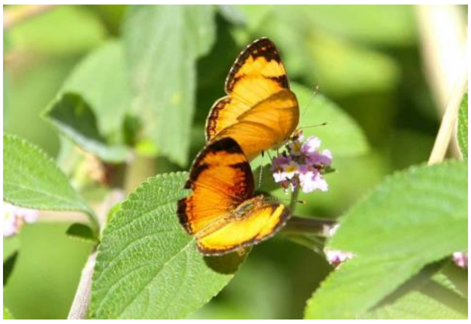

PHOTO ID: 54
Valence mean (95% CI): 7.3 (7.0; 7.6)

Arousal mean (95% CI): 3.3 (3.0; 3.7)

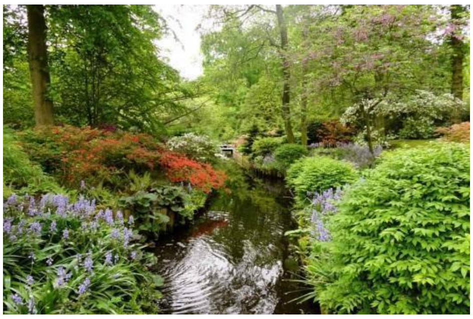

PHOTO ID: 116
Valence mean (95% CI): 7.5 (7.2; 7.8)
Arousal mean (95% CI): 3.5 (3.1; 4.0)

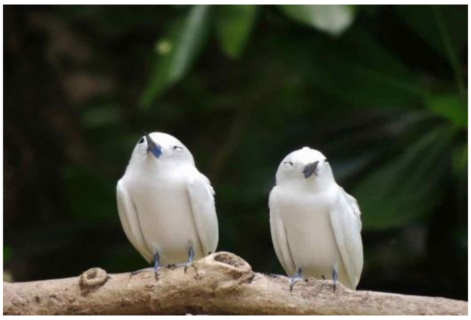

PHOTO ID: 280
Valence mean (95% CI): 7.5 (7.2; 7.8)
Arousal mean (95% CI): 3.5 (3.1; 4.1)

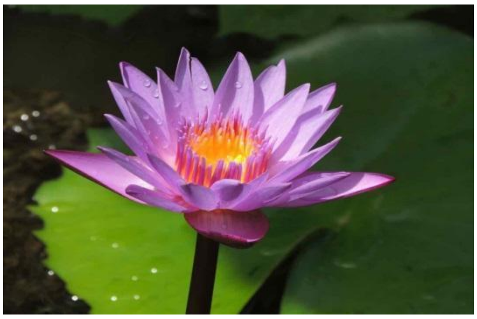

PHOTO ID: 379
Valence mean (95% CI): 7.5 (7.2; 7.8)
Arousal mean (95% CI): 3.5 (3.0; 4.0)

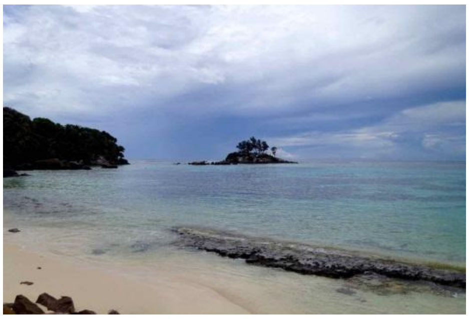

PHOTO ID: 347
Valence mean (95% CI): 7.4 (7.1; 7.7)

Arousal mean (95% CI): 3.4 (3.0; 3.9)

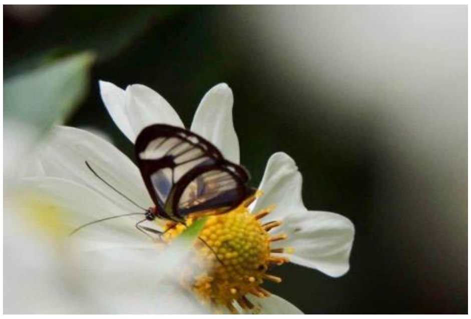

PHOTO ID: 371
Valence mean (95% CI): 7.4 (7.1; 7.7)
Arousal mean (95% CI): 3.4 (2.9; 4.0)

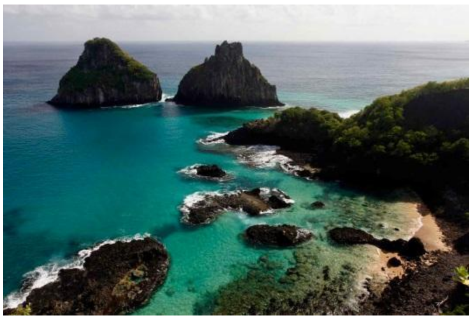

PHOTO ID: 387
Valence mean (95% CI): 7.4 (7.1; 7.8)
Arousal mean (95% CI): 3.4 (3.0; 3.9)

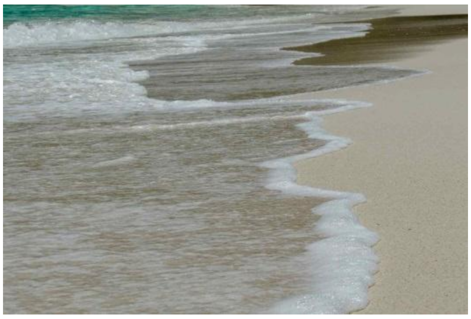

PHOTO ID: 49
Valence mean (95% CI): 7.1 (6.7; 7.5)
Arousal mean (95% CI): 3.2 (2.7; 3.7)

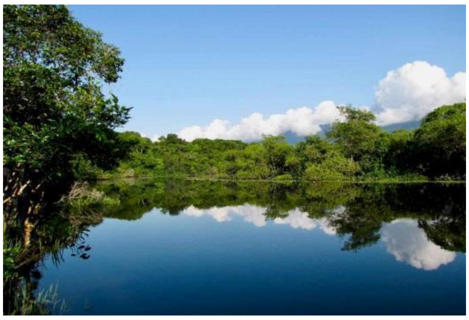

PHOTO ID: 112
Valence mean (95% CI): 7.1 (6.7; 7.5)

Arousal mean (95% CI): 3.2 (2.8; 3.6)

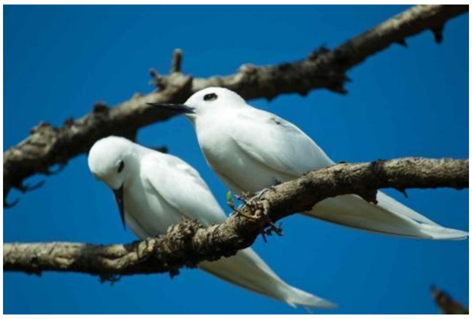

PHOTO ID: 235
Valence mean (95% CI): 7.1 (6.8; 7.5)
Arousal mean (95% CI): 3.2 (2.9; 3.6)

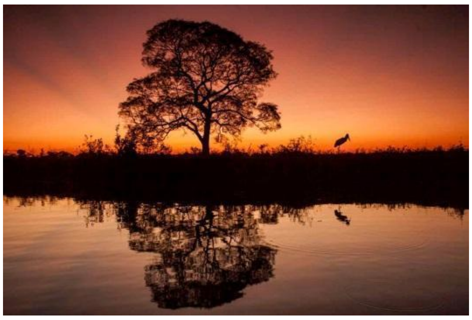

PHOTO ID: 237
Valence mean (95% CI): 7.1 (6.7; 7.5)
Arousal mean (95% CI): 3.2 (2.8; 3.7)

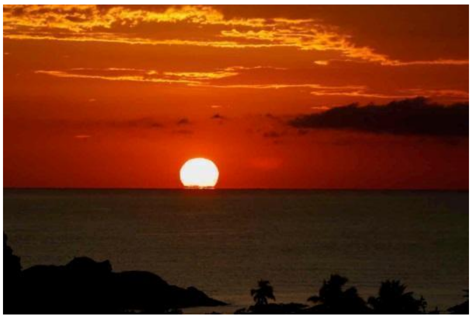

PHOTO ID: 32
Valence mean (95% CI): 7.3 (7.0; 7.6)
Arousal mean (95% CI): 3.4 (3.0; 3.8)

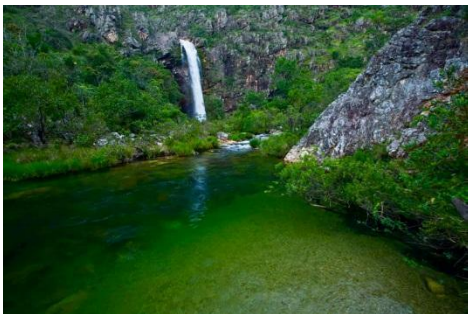

PHOTO ID: 150
Valence mean (95% CI): 7.3 (7.0; 7.5)

Arousal mean (95% CI): 3.4 (3.0; 3.9)

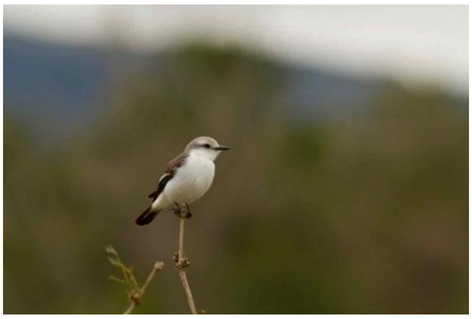

PHOTO ID: 165
Valence mean (95% CI): 7.3 (7.0; 7.6)
Arousal mean (95% CI): 3.4 (3.1; 3.9)

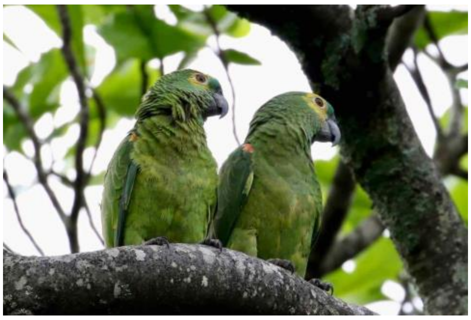

PHOTO ID: 168
Valence mean (95% CI): 7.8 (7.5; 8.0)
Arousal mean (95% CI): 3.9 (3.4; 4.4)

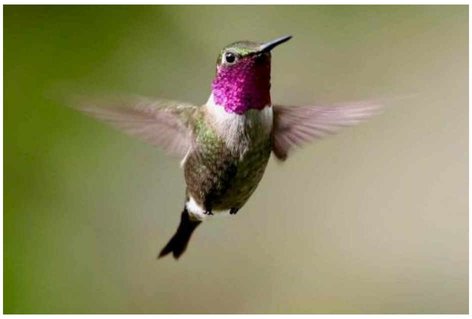

PHOTO ID: 186
Valence mean (95% CI): 7.5 (7.1; 7.8)
Arousal mean (95% CI): 3.6 (3.2; 4.2)

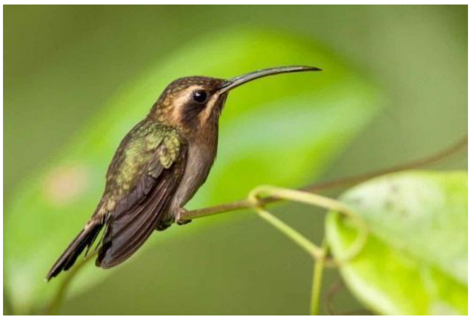

PHOTO ID: 56
Valence mean (95% CI): 7.2 (7.0; 7.4)

Arousal mean (95% CI): 3.3 (3.0; 3.7)

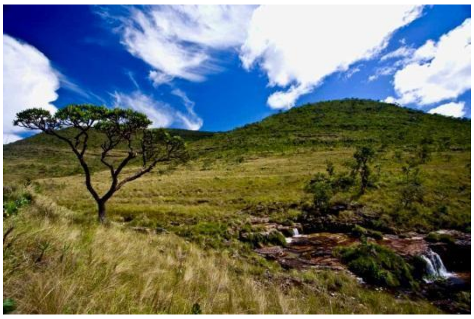

PHOTO ID: 82
Valence mean (95% CI): 7.2 (6.9; 7.5)
Arousal mean (95% CI): 3.3 (2.9; 3.8)

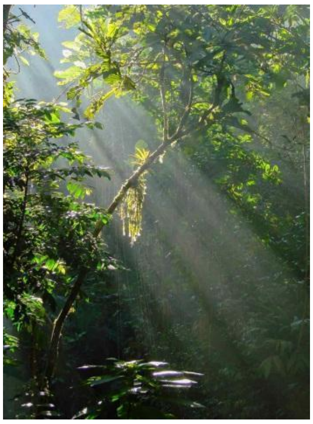

PHOTO ID: 179
Valence mean (95% CI): 7.4 (7.1; 7.8)
Arousal mean (95% CI): 3.5 (3.1; 4.0)

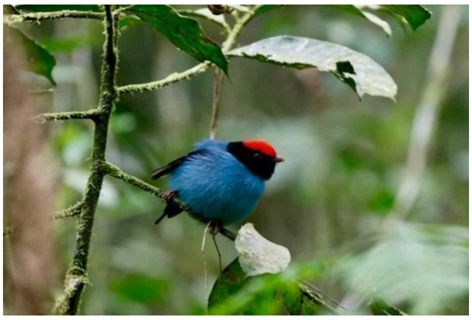

PHOTO ID: 254
Valence mean (95% CI): 7.2 (6.9; 7.5)

Arousal mean (95% CI): 3.3 (2.9; 3.7)

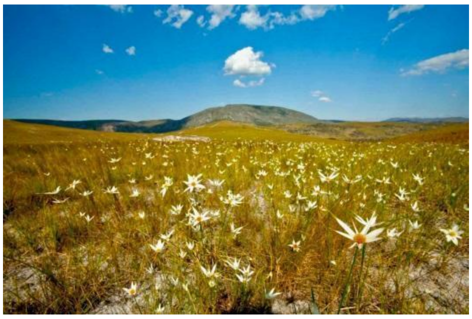

PHOTO ID: 329
Valence mean (95% CI): 7.4 (7.0; 7.9)
Arousal mean (95% CI): 3.5 (3.0; 4.0)

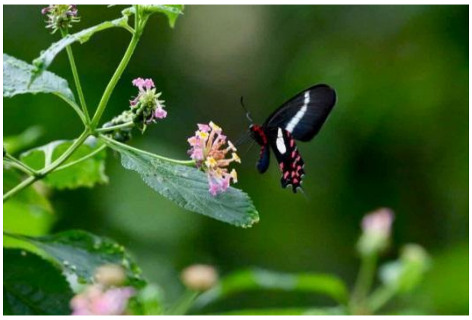

PHOTO ID: 394
Valence mean (95% CI): 7.2 (6.8; 7.6)
Arousal mean (95% CI): 3.3 (2.8; 3.8)

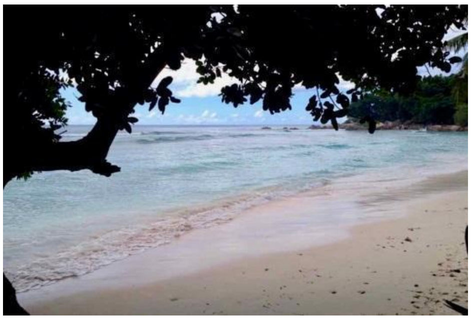

PHOTO ID: 398
Valence mean (95% CI): 7.4 (7.1; 7.8)
Arousal mean (95% CI): 3.5 (3.0; 4.1)

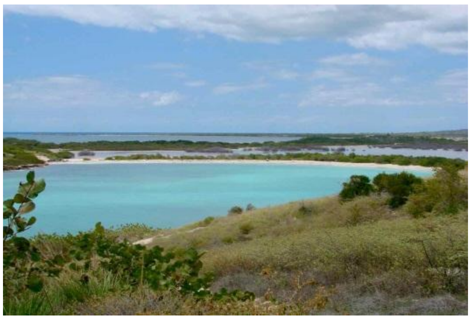

PHOTO ID: 1
Valence mean (95% CI): 7.3 (6.9; 7.6)

Arousal mean (95% CI): 3.5 (3.0; 4.1)

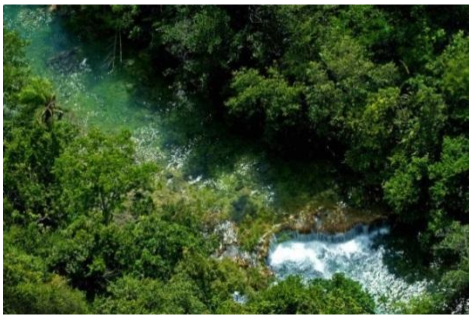

PHOTO ID: 39
Valence mean (95% CI): 7.3 (6.9; 7.7)
Arousal mean (95% CI): 3.5 (3.1; 4.0)

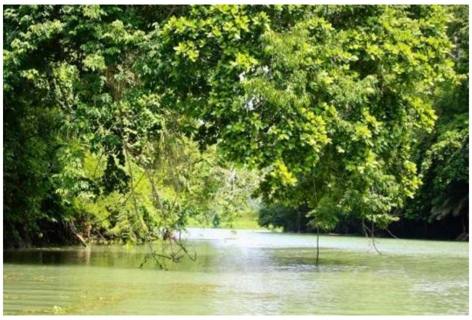

PHOTO ID: 113
Valence mean (95% CI): 7.0 (6.6; 7.5)
Arousal mean (95% CI): 3.2 (2.8; 3.6)

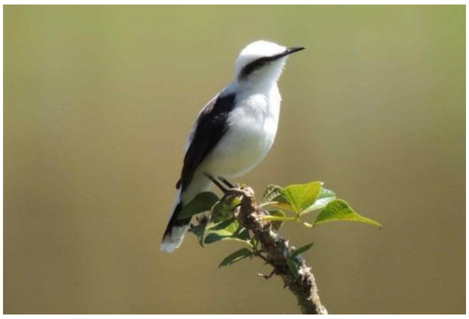

PHOTO ID: 153
Valence mean (95% CI): 7.6 (7.2; 8.1)
Arousal mean (95% CI): 3.8 (3.3; 4.4)

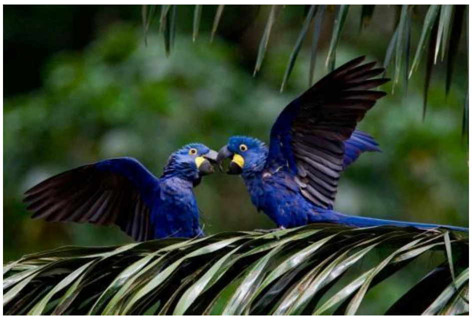

PHOTO ID: 164
Valence mean (95% CI): 7.5 (7.1; 8.0)

Arousal mean (95% CI): 3.7 (3.2; 4.2)

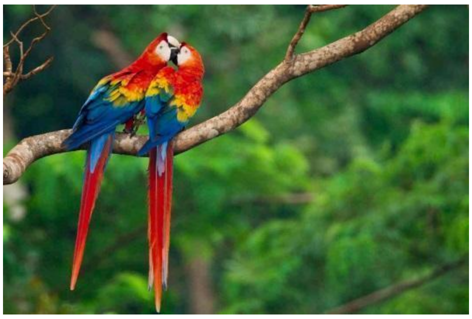

PHOTO ID: 238
Valence mean (95% CI): 6.6 (6.3; 7.0)
Arousal mean (95% CI): 2.8 (2.4; 3.3)

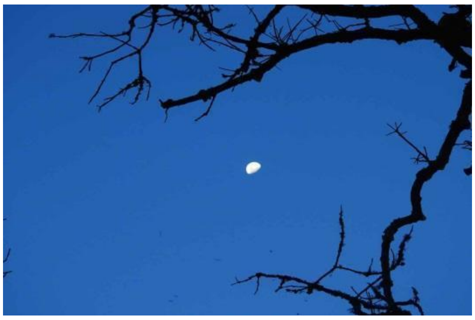

PHOTO ID: 242
Valence mean (95% CI): 7.3 (7.0; 7.6)
Arousal mean (95% CI): 3.5 (3.1; 3.9)

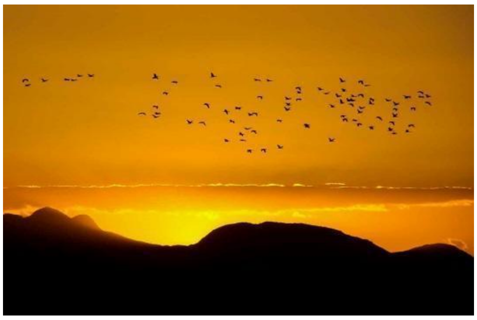

PHOTO ID: 243
Valence mean (95% CI): 7.1 (6.8; 7.5)

Arousal mean (95% CI): 3.3 (2.9; 3.7)

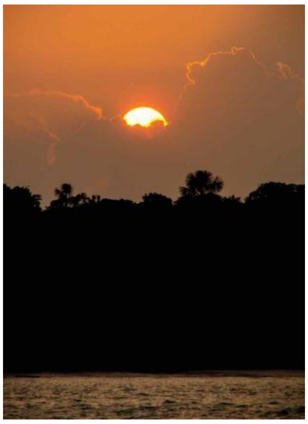

PHOTO ID: 285
Valence mean (95% CI): 7.0 (6.7; 7.4)
Arousal mean (95% CI): 3.2 (2.9; 3.6)

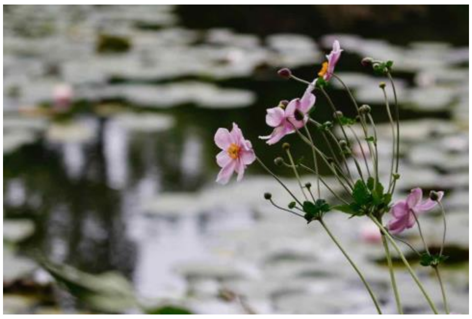

PHOTO ID: 328
Valence mean (95% CI): 7.3 (7.1; 7.6)
Arousal mean (95% CI): 3.5 (3.1; 4.0)

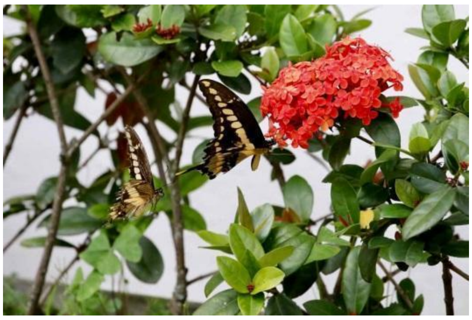

PHOTO ID: 15
Valence mean (95% CI): 7.7 (7.3; 8.1)

Arousal mean (95% CI): 3.9 (3.4; 4.6)

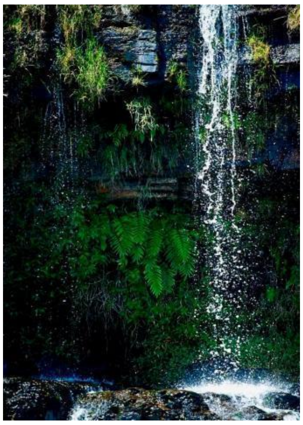

PHOTO ID: 36
Valence mean (95% CI): 7.2 (6.9; 7.6)
Arousal mean (95% CI): 3.4 (3.0; 3.9)

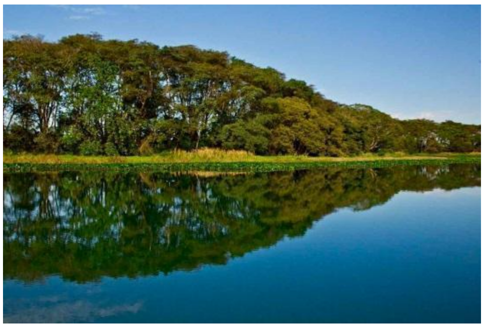

PHOTO ID: 139
Valence mean (95% CI): 7.2 (6.9; 7.6)
Arousal mean (95% CI): 3.4 (3.0; 3.8)

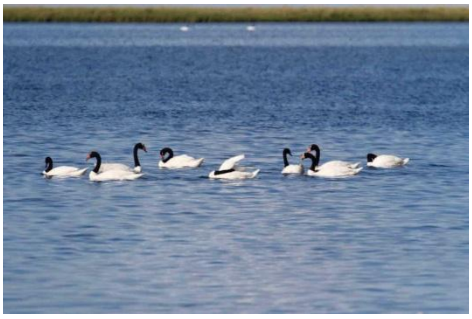

PHOTO ID: 154
Valence mean (95% CI): 7.7 (7.3; 8.1)

Arousal mean (95% CI): 3.9 (3.4; 4.4)

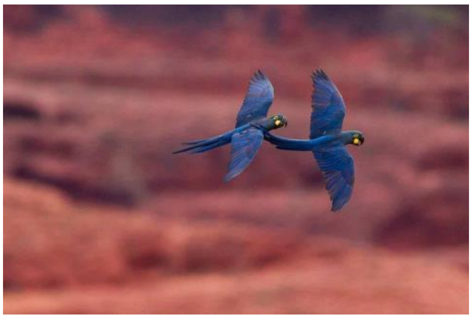

PHOTO ID: 178
Valence mean (95% CI): 7.2 (6.9; 7.6)
Arousal mean (95% CI): 3.4 (3.1; 3.9)

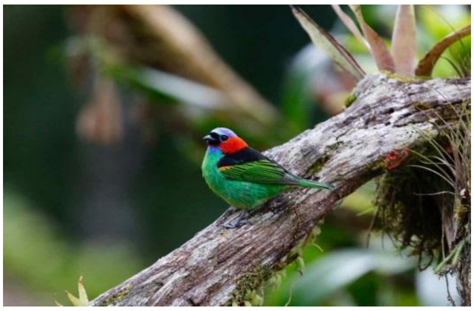

PHOTO ID: 202
Valence mean (95% CI): 7.4 (7.0; 7.7)
Arousal mean (95% CI): 3.6 (3.1; 4.1)

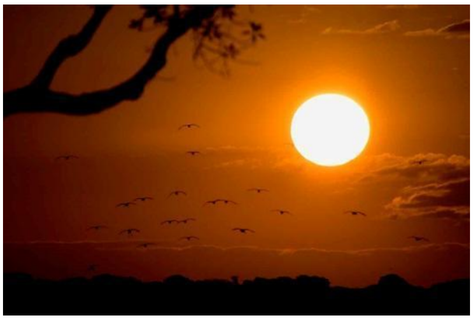

PHOTO ID: 245
Valence mean (95% CI): 7.2 (6.7; 7.6)

Arousal mean (95% CI): 3.4 (2.9; 4.0)

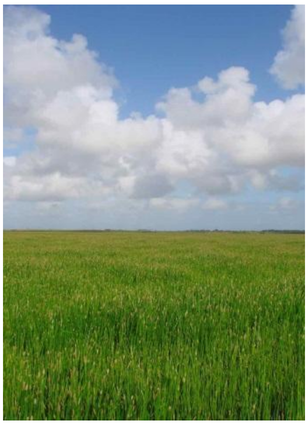

PHOTO ID: 246
Valence mean (95% CI): 7.2 (6.8; 7.6)
Arousal mean (95% CI): 3.4 (3.0; 3.9)

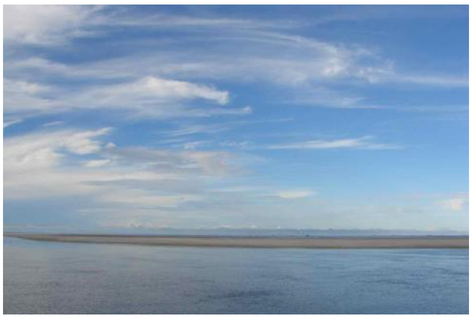

PHOTO ID: 384
Valence mean (95% CI): 7.2 (6.8; 7.6)
Arousal mean (95% CI): 3.4 (3.0; 4.0)

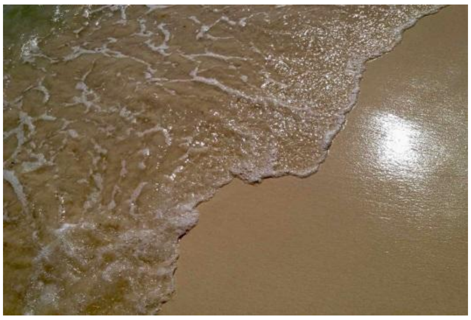

PHOTO ID: 391
Valence mean (95% CI): 7.4 (7.1; 7.8)

Arousal mean (95% CI): 3.6 (3.1; 4.2)

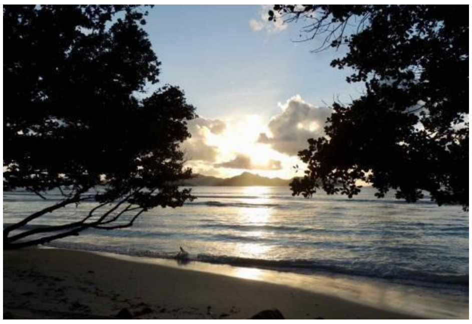

PHOTO ID: 118
Valence mean (95% CI): 7.1 (6.8; 7.4)
Arousal mean (95% CI): 3.4 (3.0; 3.9)

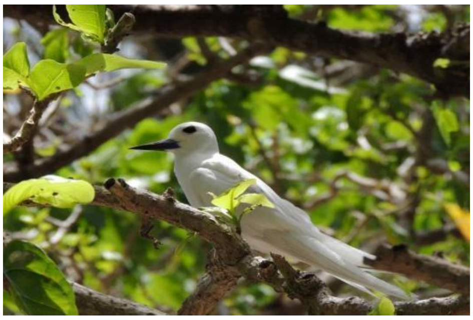

PHOTO ID: 169
Valence mean (95% CI): 7.3 (6.9; 7.7)
Arousal mean (95% CI): 3.6 (3.1; 4.2)

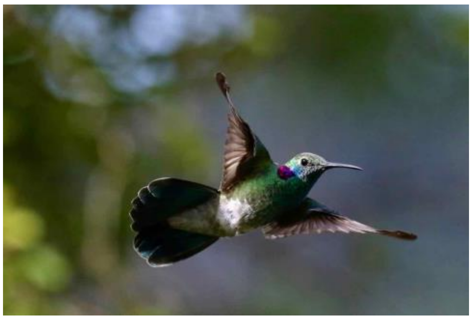

PHOTO ID: 174
Valence mean (95% CI): 7.3 (7.0; 7.6)
Arousal mean (95% CI): 3.6 (3.2; 4.2)

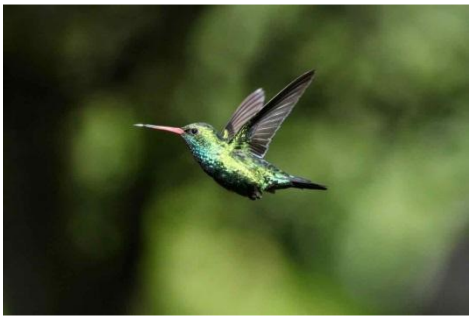

PHOTO ID: 187
Valence mean (95% CI): 7.3 (7.0; 7.7)

Arousal mean (95% CI): 3.6 (3.2; 4.0)

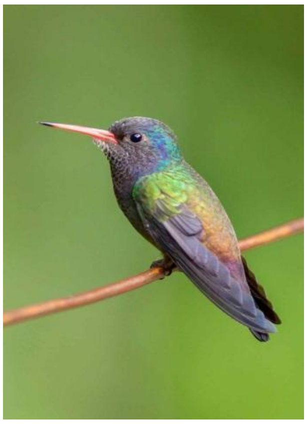

PHOTO ID: 195
Valence mean (95% CI): 7.6 (7.3; 7.9)
Arousal mean (95% CI): 3.9 (3.4; 4.5)

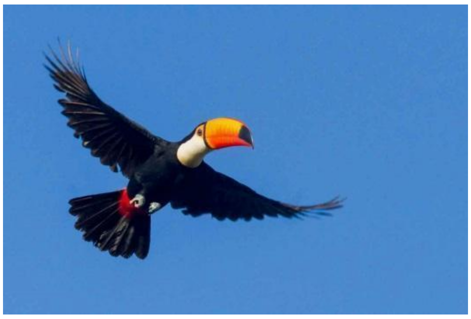

PHOTO ID: 200
Valence mean (95% CI): 7.3 (7.0; 7.6)
Arousal mean (95% CI): 3.6 (3.2; 4.1)

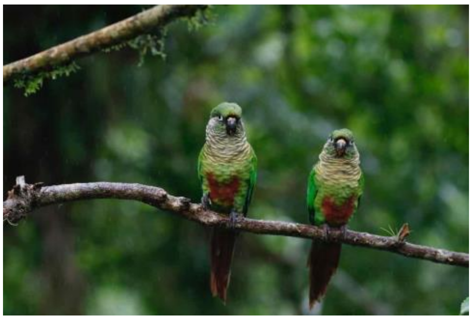

PHOTO ID: 244
Valence mean (95% CI): 7.3 (6.9; 7.7)

Arousal mean (95% CI): 3.6 (3.2; 4.2)

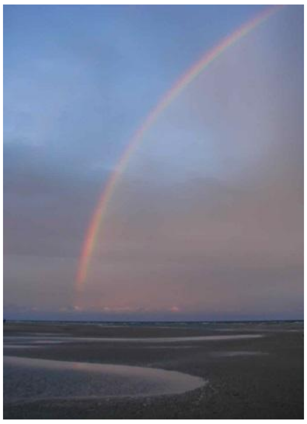

PHOTO ID: 263
Valence mean (95% CI): 7.1 (6.7; 7.4)
Arousal mean (95% CI): 3.4 (3.0; 3.9)

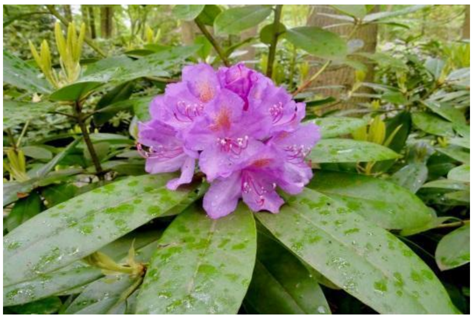

PHOTO ID: 286
Valence mean (95% CI): 7.3 (6.9; 7.7)
Arousal mean (95% CI): 3.6 (3.1; 4.0)

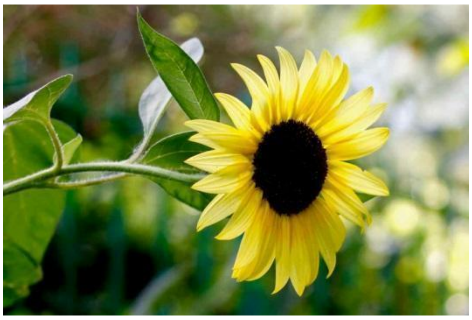

PHOTO ID: 443
Valence mean (95% CI): 7.3 (7.0; 7.6)

Arousal mean (95% CI): 3.6 (3.2; 4.1)

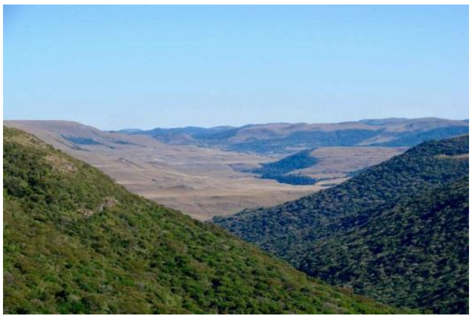

PHOTO ID: 86
Valence mean (95% CI): 7.2 (7.0; 7.5)
Arousal mean (95% CI): 3.5 (3.1; 3.9)

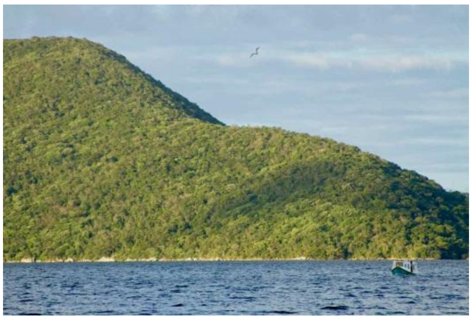

PHOTO ID: 128
Valence mean (95% CI): 7.5 (7.2; 7.8)
Arousal mean (95% CI): 3.8 (3.3; 4.4)

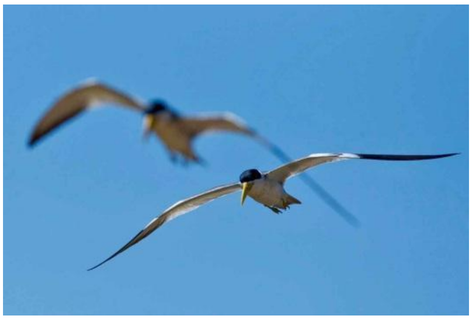

PHOTO ID: 177
Valence mean (95% CI): 7.5 (7.2; 7.8)
Arousal mean (95% CI): 3.8 (3.4; 4.3)

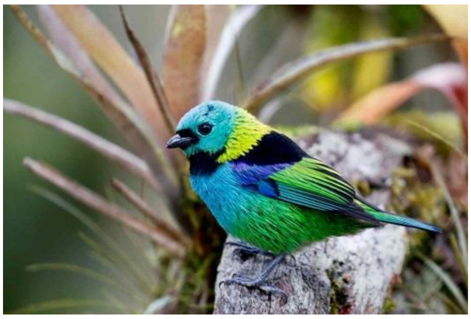

PHOTO ID: 188
Valence mean (95% CI): 7.2 (6.9; 7.5)

Arousal mean (95% CI): 3.5 (3.2; 3.9)

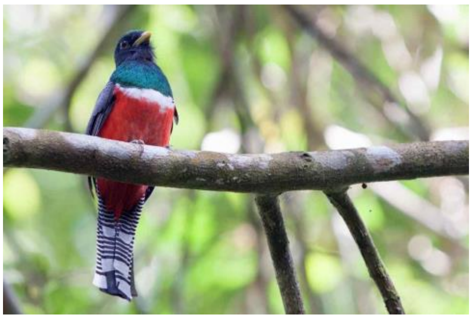

PHOTO ID: 213
Valence mean (95% CI): 6.7 (6.3; 7.1)
Arousal mean (95% CI): 3.0 (2.6; 3.5)

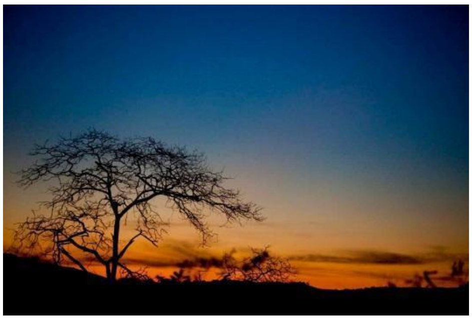

PHOTO ID: 262
Valence mean (95% CI): 7.5 (7.2; 7.8)
Arousal mean (95% CI): 3.8 (3.3; 4.3)

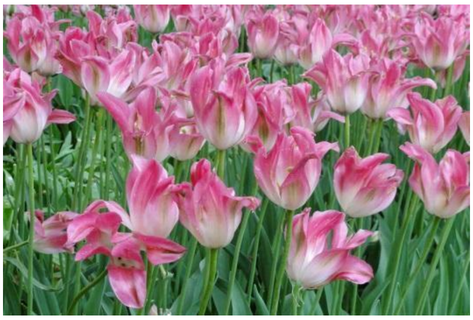

PHOTO ID: 279
Valence mean (95% CI): 7.4 (7.1; 7.7)
Arousal mean (95% CI): 3.7 (3.3; 4.3)

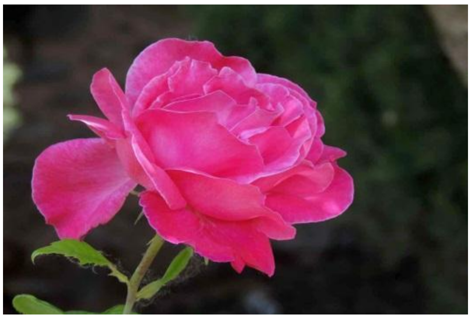

PHOTO ID: 283
Valence mean (95% CI): 7.0 (6.6; 7.4)

Arousal mean (95% CI): 3.3 (2.9; 3.7)

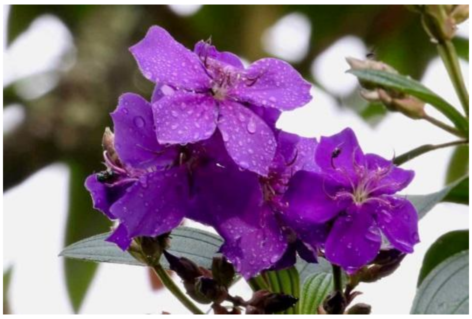

PHOTO ID: 284
Valence mean (95% CI): 7.2 (6.9; 7.4)
Arousal mean (95% CI): 3.5 (3.1; 3.9)

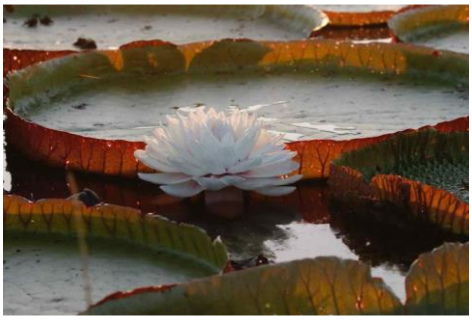

PHOTO ID: 330
Valence mean (95% CI): 7.2 (6.9; 7.5)
Arousal mean (95% CI): 3.5 (3.2; 4.0)

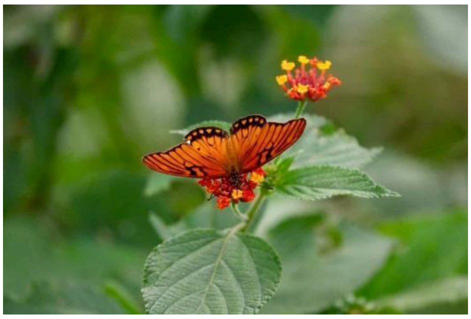

PHOTO ID: 151
Valence mean (95% CI): 7.1 (6.8; 7.4)
Arousal mean (95% CI): 3.5 (3.1; 3.9)

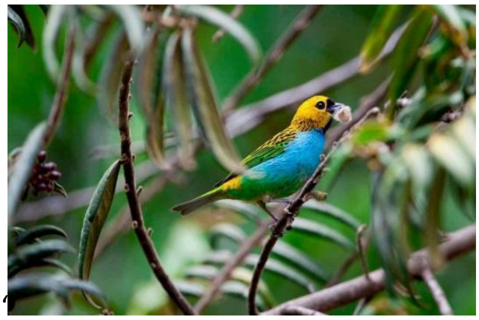

PHOTO ID: 348
Valence mean (95% CI): 7.3 (7.0; 7.6)

Arousal mean (95% CI): 3.7 (3.2; 4.2)

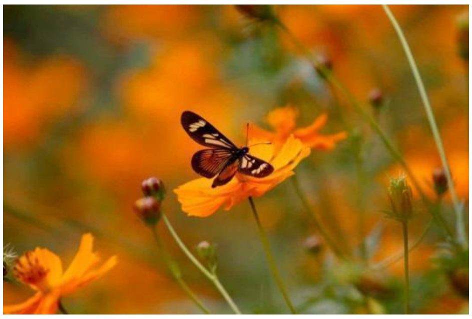

PHOTO ID: 354
Valence mean (95% CI): 7.1 (6.8; 7.4)
Arousal mean (95% CI): 3.5 (3.0; 4.0)

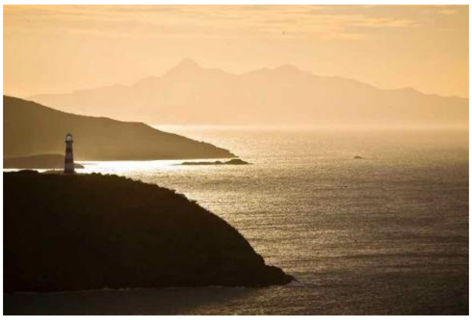

PHOTO ID: 48
Valence mean (95% CI): 7.2 (6.8; 7.6)
Arousal mean (95% CI): 3.6 (3.1; 4.1)

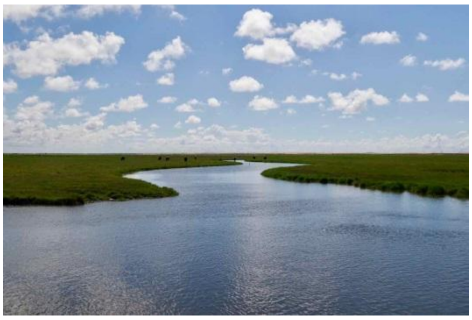

PHOTO ID: 55
Valence mean (95% CI): 7.0 (6.7; 7.4)
Arousal mean (95% CI): 3.4 (3.1; 3.8)

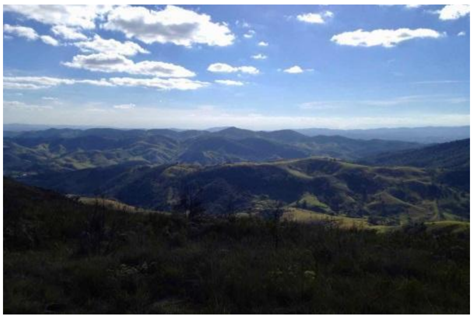

PHOTO ID: 114
Valence mean (95% CI): 7.2 (6.9; 7.5)

Arousal mean (95% CI): 3.6 (3.1; 4.0)

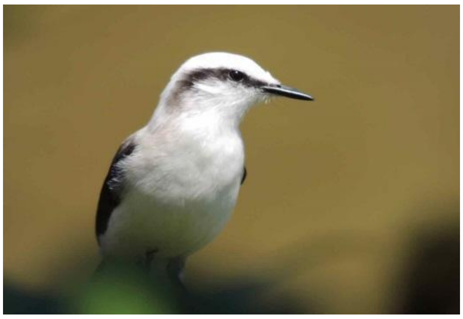

PHOTO ID: 119
Valence mean (95% CI): 7.0 (6.6; 7.4)
Arousal mean (95% CI): 3.4 (3.0; 3.9)

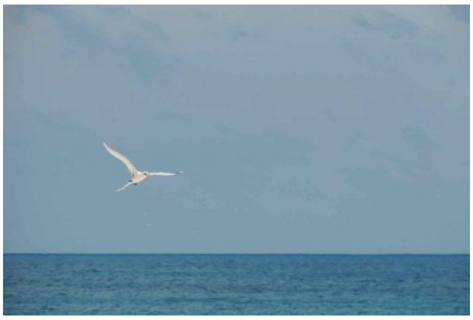

PHOTO ID: 203
Valence mean (95% CI): 7.2 (6.8; 7.6)
Arousal mean (95% CI): 3.6 (3.1; 4.2)

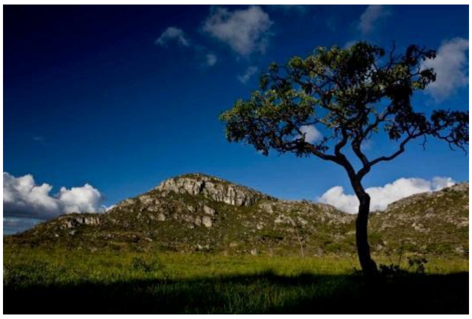

PHOTO ID: 258
Valence mean (95% CI): 7.2 (7.0; 7.5)

Arousal mean (95% CI): 3.6 (3.2; 4.0)

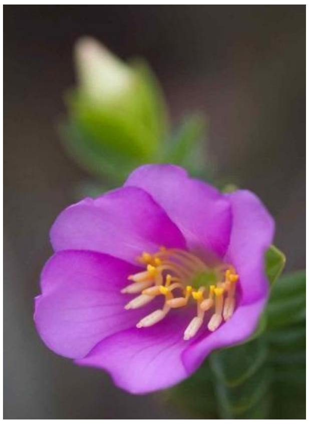

PHOTO ID: 321
Valence mean (95% CI): 7.2 (6.9; 7.4)
Arousal mean (95% CI): 3.6 (3.2; 4.0)

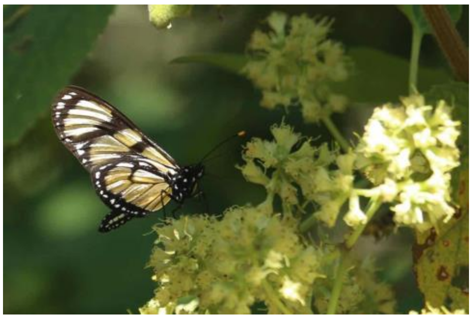

PHOTO ID: 349
Valence mean (95% CI): 7.2 (6.9; 7.6)
Arousal mean (95% CI): 3.6 (3.2; 4.2)

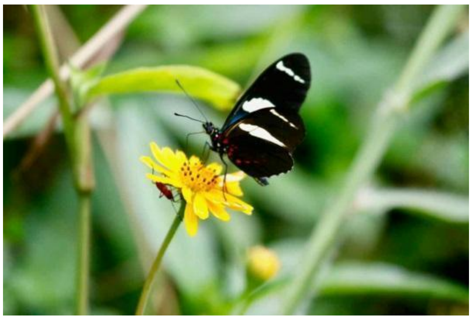

PHOTO ID: 374
Valence mean (95% CI): 7.5 (7.2; 7.8)

Arousal mean (95% CI): 3.9 (3.4; 4.4)

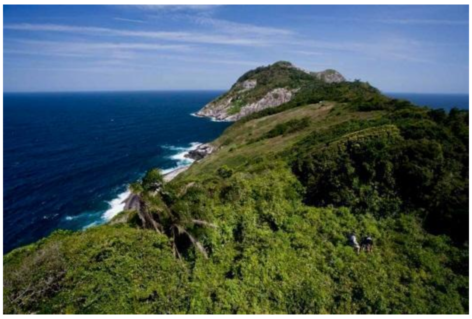

PHOTO ID: 173
Valence mean (95% CI): 7.4 (7.0; 7.8)
Arousal mean (95% CI): 3.8 (3.4; 4.3)

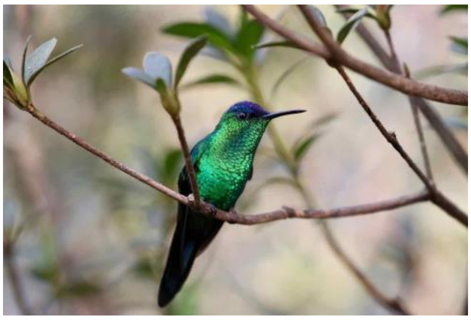

PHOTO ID: 275
Valence mean (95% CI): 7.4 (7.1; 7.7)
Arousal mean (95% CI): 3.8 (3.4; 4.1)

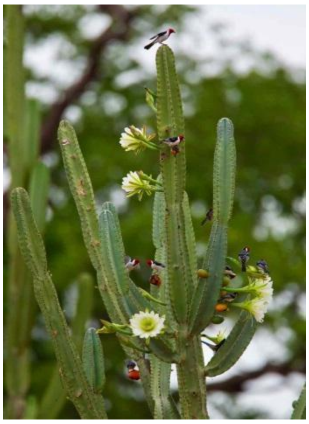

PHOTO ID: 212
Valence mean (95% CI): 7.1 (6.8; 7.5)

Arousal mean (95% CI): 3.6 (3.1; 4.1)

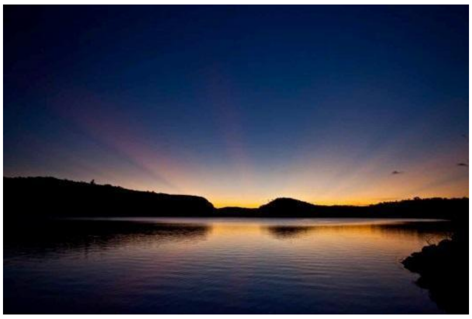

PHOTO ID: 227
Valence mean (95% CI): 7.1 (6.8; 7.3)
Arousal mean (95% CI): 3.6 (3.2; 4.1)

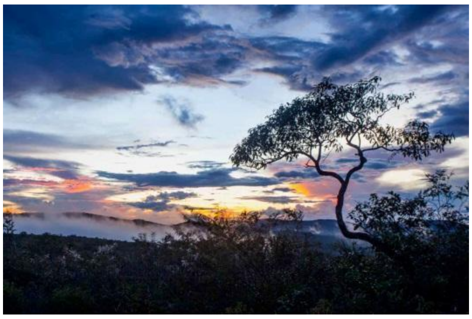

PHOTO ID: 231
Valence mean (95% CI): 7.1 (6.7; 7.5)
Arousal mean (95% CI): 3.6 (3.1; 4.2)

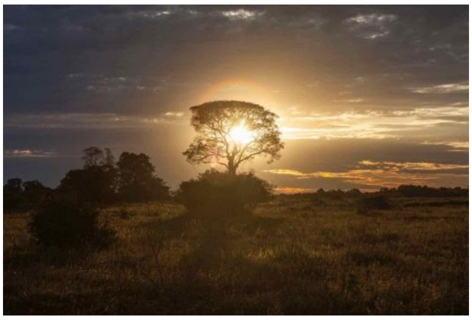

PHOTO ID: 31
Valence mean (95% CI): 7.5 (7.1; 7.9)

Arousal mean (95% CI): 4.0 (3.5; 4.5)

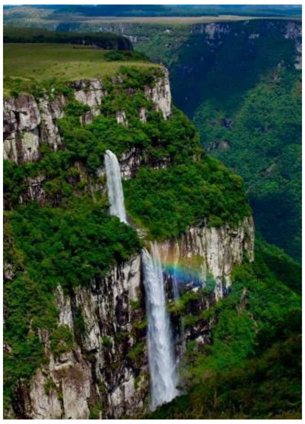

PHOTO ID: 37
Valence mean (95% CI): 6.9 (6.7; 7.2)
Arousal mean (95% CI): 3.4 (3.0; 3.9)

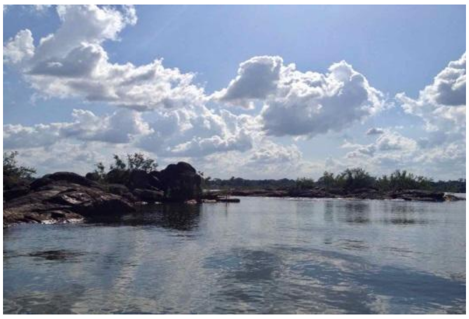

PHOTO ID: 45
Valence mean (95% CI): 6.9 (6.5; 7.3)
Arousal mean (95% CI): 3.4 (3.0; 3.9)

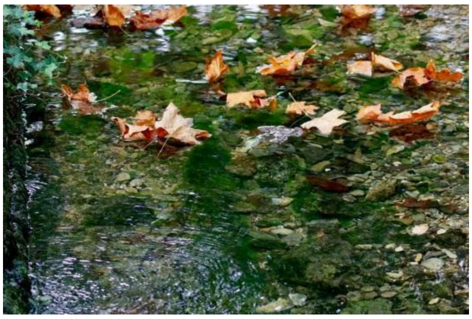

PHOTO ID: 58
Valence mean (95% CI): 7.3 (7.1; 7.6)

Arousal mean (95% CI): 3.8 (3.4; 4.2)

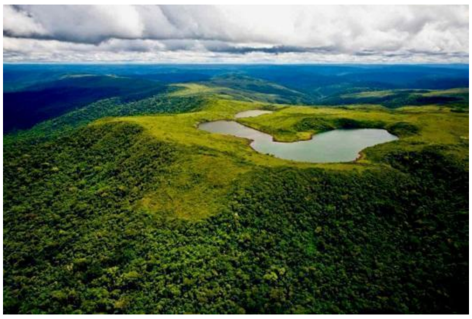

PHOTO ID: 117
Valence mean (95% CI): 7.0 (6.6; 7.4)
Arousal mean (95% CI): 3.5 (3.1; 4.0)

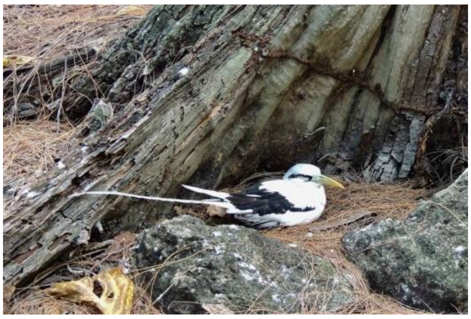

PHOTO ID: 239
Valence mean (95% CI): 6.8 (6.4; 7.3)
Arousal mean (95% CI): 3.3 (2.8; 3.8)

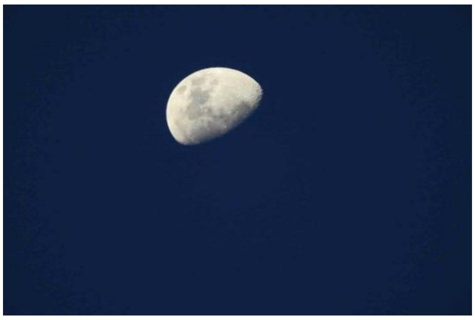

PHOTO ID: 252
Valence mean (95% CI): 6.9 (6.5; 7.3)
Arousal mean (95% CI): 3.4 (3.0; 3.9)

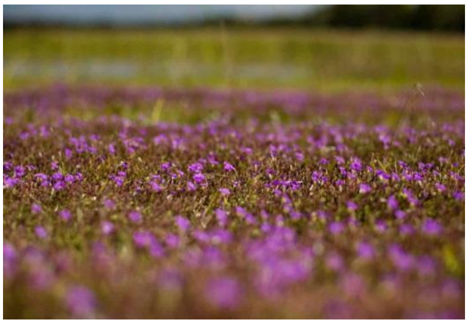

PHOTO ID: 294
Valence mean (95% CI): 7.3 (7.1; 7.6)

Arousal mean (95% CI): 3.8 (3.4; 4.3)

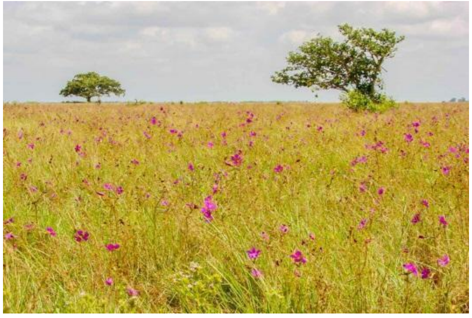

PHOTO ID: 299
Valence mean (95% CI): 6.9 (6.4; 7.3)
Arousal mean (95% CI): 3.4 3.4 (3.0; 3.9)

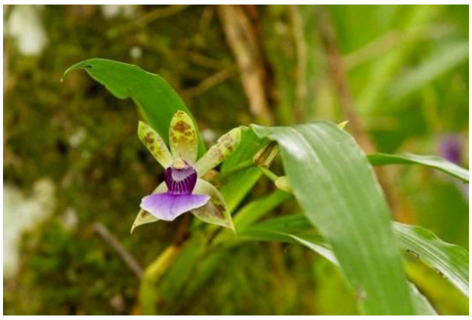

PHOTO ID: 364
Valence mean (95% CI): 7.3 (7.0; 7.6)
Arousal mean (95% CI): 3.8 (3.3; 4.3)

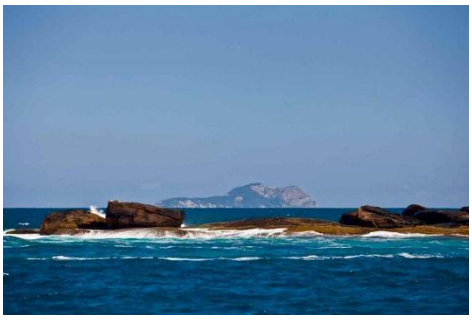

PHOTO ID: 448
Valence mean (95% CI): 7.3 (7.0; 7.7)
Arousal mean (95% CI): 3.8 (3.4; 4.4)

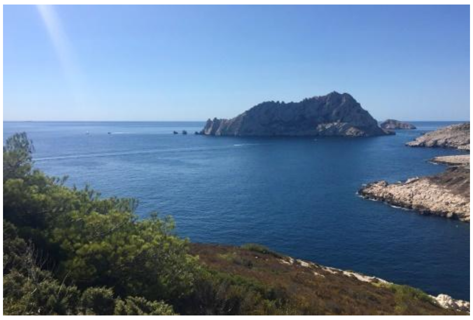

PHOTO ID: 167
Valence mean (95% CI): 7.4 (7.0; 7.8)

Arousal mean (95% CI): 3.9 (3.4; 4.4)

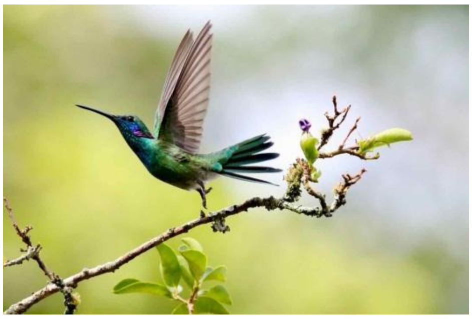

PHOTO ID: 2
Valence mean (95% CI): 7.1 (6.7; 7.4)
Arousal mean (95% CI): 3.7 (3.3; 4.1)

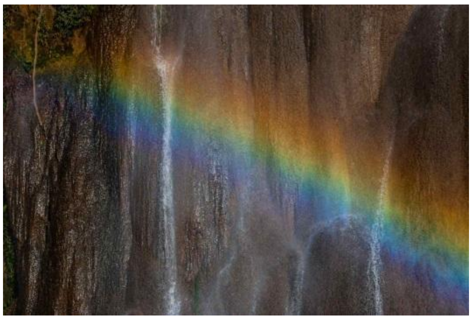

PHOTO ID: 50
Valence mean (95% CI): 7.6 (7.2; 8.0)
Arousal mean (95% CI): 4.2 (3.7; 4.9)

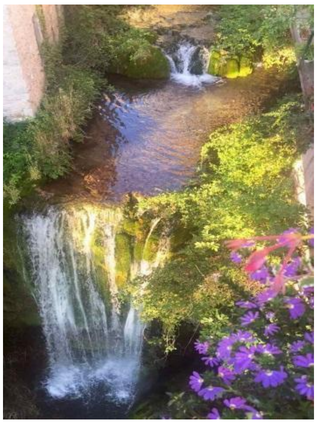

PHOTO ID: 240
Valence mean (95% CI): 7.1 (6.8; 7.5)

Arousal mean (95% CI): 3.7 (3.1; 4.3)

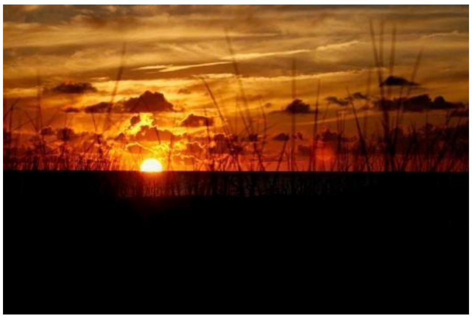

PHOTO ID: 355
Valence mean (95% CI): 7.1 (6.6; 7.6)
Arousal mean (95% CI): 3.7 (3.2; 4.4)

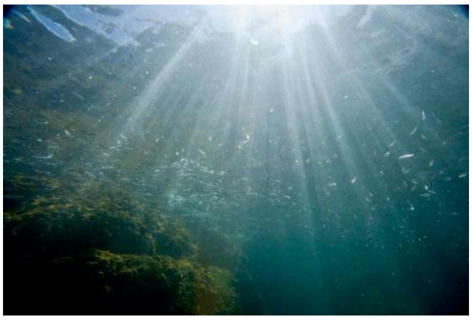

PHOTO ID: 27
Valence mean (95% CI): 6.8 (6.4; 7.2)
Arousal mean (95% CI): 3.4 (3.0; 3.8)

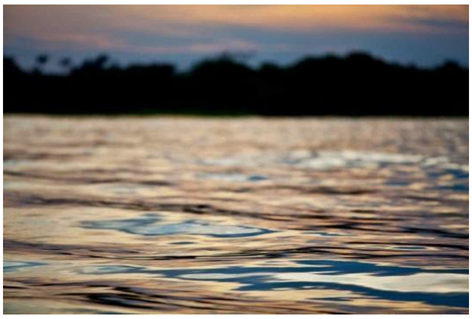

PHOTO ID: 28
Valence mean (95% CI): 7.0 (6.8; 7.3)
Arousal mean (95% CI): 3.6 (3.2; 4.0)

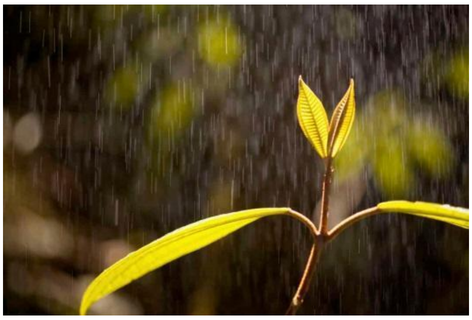

PHOTO ID: 46
Valence mean (95% CI): 6.9 (6.5; 7.3)

Arousal mean (95% CI): 3.5 (3.1; 4.0)

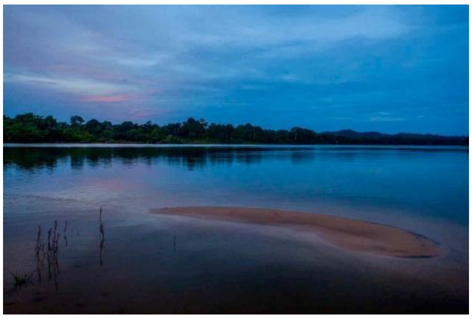

PHOTO ID: 77
Valence mean (95% CI): 7.0 (6.7; 7.3)
Arousal mean (95% CI): 3.6 (3.3; 4.0)

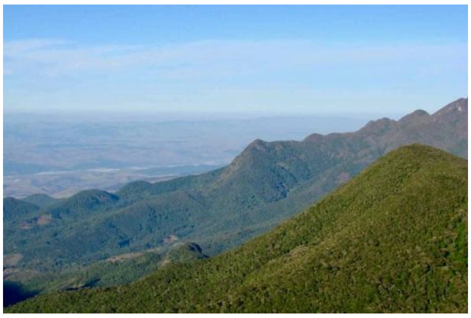

PHOTO ID: 138
Valence mean (95% CI): 7.3 (7.0; 7.6)
Arousal mean (95% CI): 3.9 (3.5; 4.4)

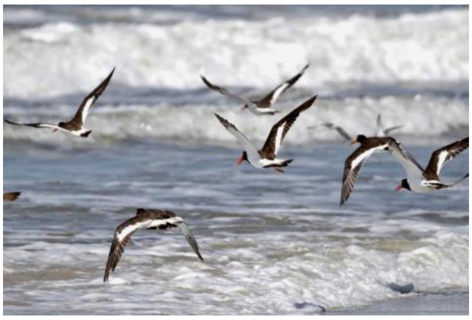

PHOTO ID: 166
Valence mean (95% CI): 7.3 (7.0; 7.6)
Arousal mean (95% CI): 3.9 (3.5; 4.4)

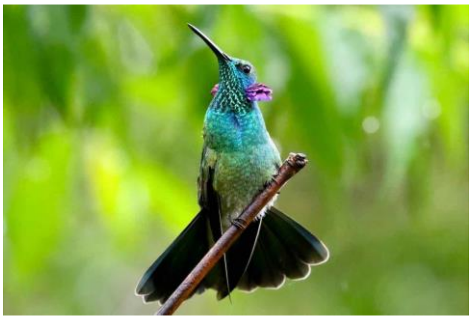

PHOTO ID: 176
Valence mean (95% CI): 7.5 (7.2; 7.8)

Arousal mean (95% CI): 4.1 (3.6; 4.7)

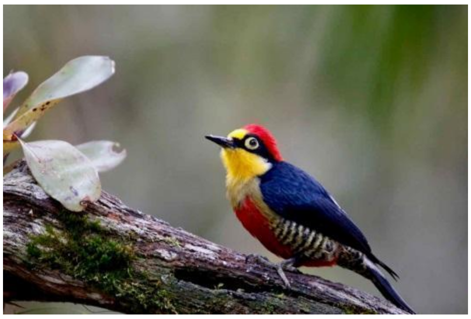

PHOTO ID: 201
Valence mean (95% CI): 7.0 (6.6; 7.4)
Arousal mean (95% CI): 3.6 (3.2; 4.2)

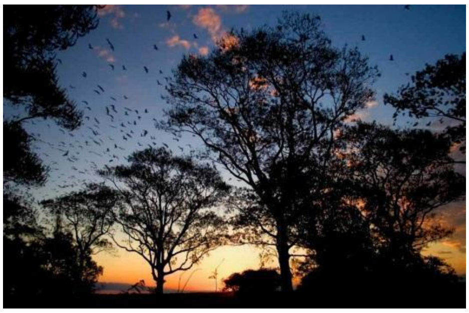

PHOTO ID: 229
Valence mean (95% CI): 6.8 (6.4; 7.2)
Arousal mean (95% CI): 3.4 (3.0; 3.9)

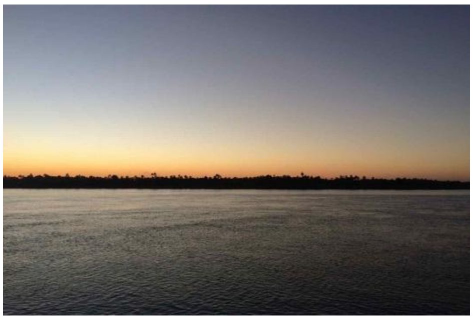

PHOTO ID: 230
Valence mean (95% CI): 7.0 (6.6; 7.4)
Arousal mean (95% CI): 3.6 (3.1; 4.1)

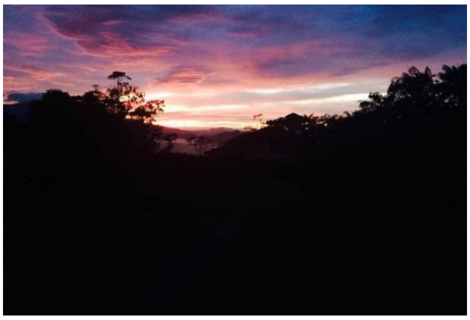

PHOTO ID: 253
Valence mean (95% CI): 7.5 (7.2; 7.8)

Arousal mean (95% CI): 4.1 (3.6; 4.7)

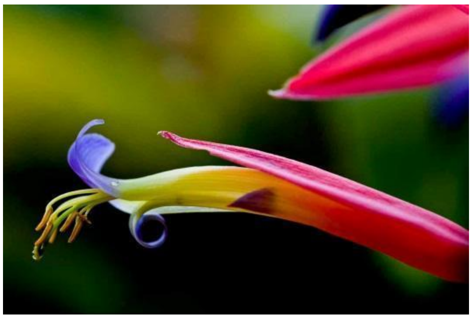

PHOTO ID: 268
Valence mean (95% CI): 6.9 (6.5; 7.4)
Arousal mean (95% CI): 3.5 (3.0; 3.9)

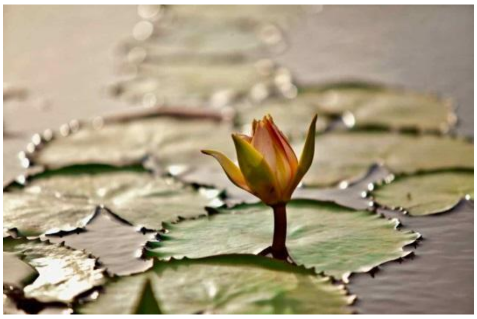

PHOTO ID: 338
Valence mean (95% CI): 7.0 (6.8; 7.3)
Arousal mean (95% CI): 3.6 (3.3; 4.1)

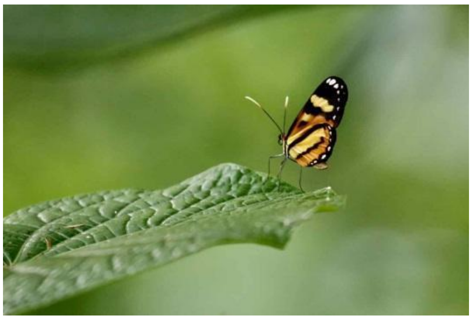

PHOTO ID: 389
Valence mean (95% CI): 7.3 (6.9; 7.7)
Arousal mean (95% CI): 3.9 (3.3; 4.6)

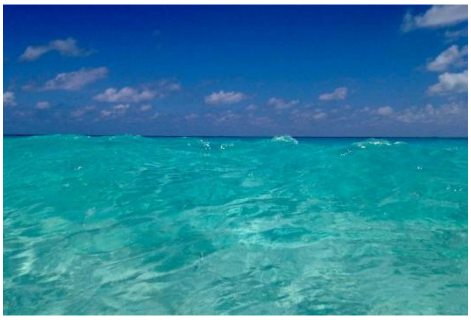

PHOTO ID: 182
Valence mean (95% CI): 7.4 (7.0; 7.7)

Arousal mean (95% CI): 4.0 (3.6; 4.6)

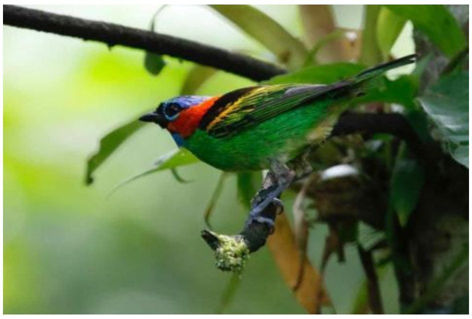

PHOTO ID: 194
Valence mean (95% CI): 7.2 (6.9; 7.6)
Arousal mean (95% CI): 3.8 (3.4; 4.4)

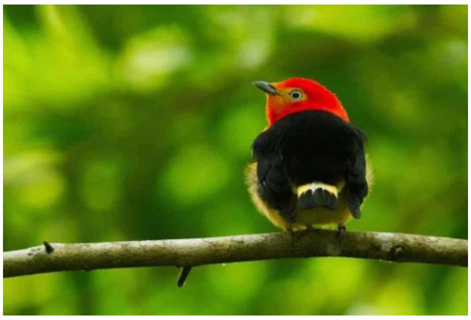

PHOTO ID: 261
Valence mean (95% CI): 7.4 (7.2; 7.7)
Arousal mean (95% CI): 4.0 (3.4; 4.6)

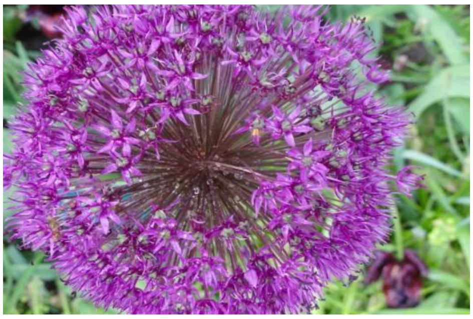

PHOTO ID: 5
Valence mean (95% CI): 7.0 (6.6; 7.3)

Arousal mean (95% CI): 3.7 (3.3; 4.2)

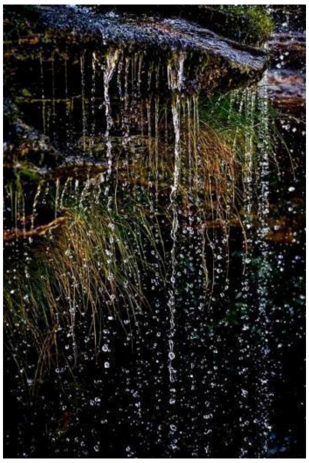

PHOTO ID: 108
Valence mean (95% CI): 6.9 (6.5; 7.2)
Arousal mean (95% CI): 3.6 (3.2; 4.1)

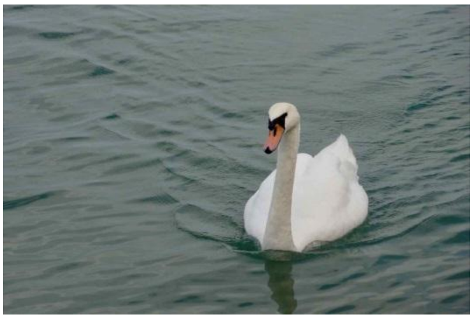

PHOTO ID: 141
Valence mean (95% CI): 7.3 (6.9; 7.6)
Arousal mean (95% CI): 4.0 (3.5; 4.7)

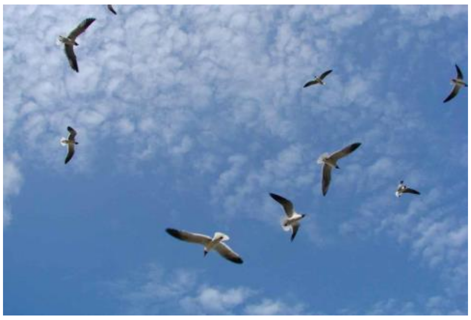

PHOTO ID: 156
Valence mean (95% CI): 7.5 (7.2; 7.8)

Arousal mean (95% CI): 4.2 (3.7; 4.7)

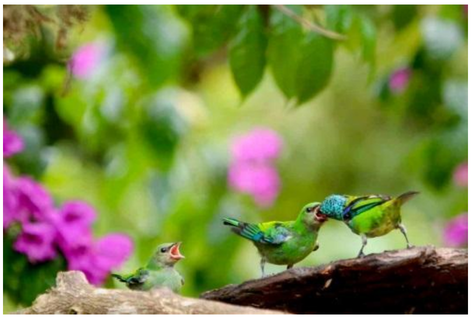

PHOTO ID: 206
Valence mean (95% CI): 7.1 (6.8; 7.4)
Arousal mean (95% CI): 3.8 (3.3; 4.3)

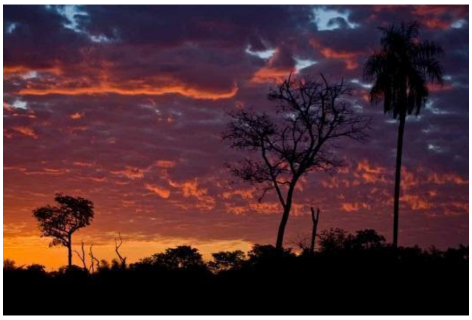

PHOTO ID: 220
Valence mean (95% CI): 6.6 (6.2; 7.0)
Arousal mean (95% CI): 3.3 (3.0; 3.7)

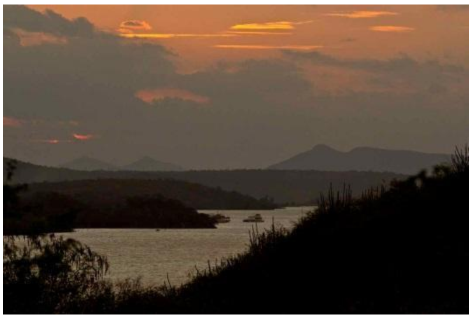

PHOTO ID: 257
Valence mean (95% CI): 7.1 (6.8; 7.4)
Arousal mean (95% CI): 3.8 (3.3; 4.3)

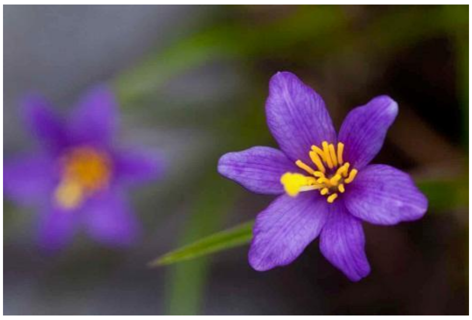

PHOTO ID: 259
Valence mean (95% CI): 7.1 (6.8; 7.5)

Arousal mean (95% CI): 3.8 (3.4; 4.3)

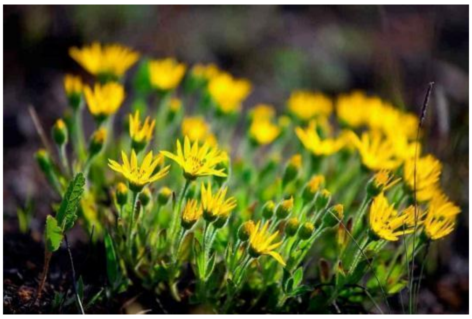

PHOTO ID: 281
Valence mean (95% CI): 6.9 (6.6; 7.2)
Arousal mean (95% CI): 3.6 (3.2; 4.2)

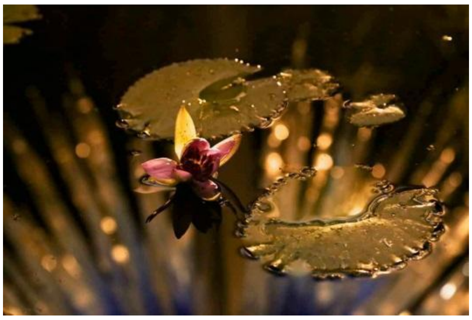

PHOTO ID: 289
Valence mean (95% CI): 6.8 (6.5; 7.1)
Arousal mean (95% CI): 3.5 (3.1; 3.9)

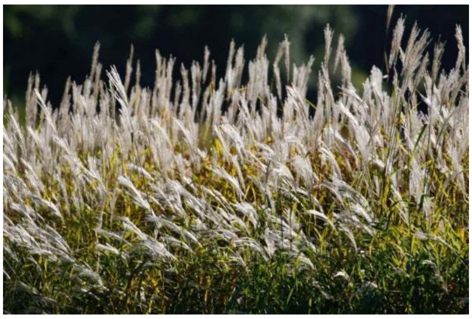

PHOTO ID: 291
Valence mean (95% CI): 6.9 (6.5; 7.3)
Arousal mean (95% CI): 3.6 (3.2; 4.0)

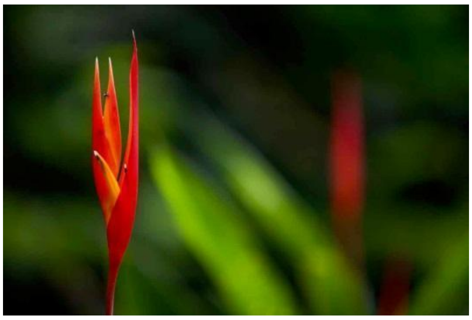

PHOTO ID: 310
Valence mean (95% CI): 7.1 (6.8; 7.4)

Arousal mean (95% CI): 3.8 (3.4; 4.3)

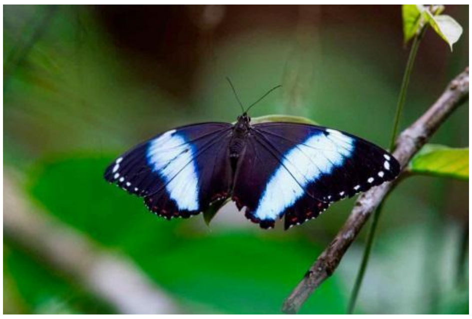

PHOTO ID: 396
Valence mean (95% CI): 7.1 (6.8; 7.4)
Arousal mean (95% CI): 3.8 (3.3; 4.4)

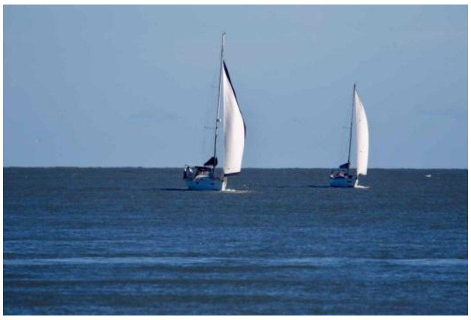

PHOTO ID: 397
Valence mean (95% CI): 7.0 (6.6; 7.4)
Arousal mean (95% CI): 3.7 (3.3; 4.3)

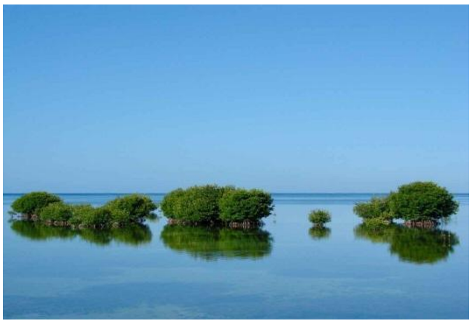

PHOTO ID: 447
Valence mean (95% CI): 6.9 (6.5; 7.3)
Arousal mean (95% CI): 3.6 (3.1; 4.2)

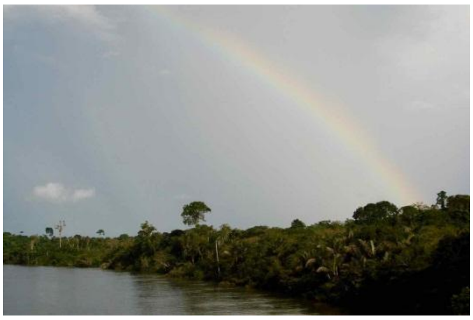

PHOTO ID: 110
Valence mean (95% CI): 6.7 (6.3; 7.1)

Arousal mean (95% CI): 3.4 (3.0; 3.9)

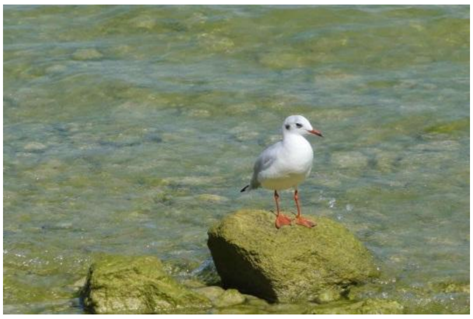

PHOTO ID: 172
Valence mean (95% CI): 7.2 (6.9; 7.5)
Arousal mean (95% CI): 3.9 (3.5; 4.3)

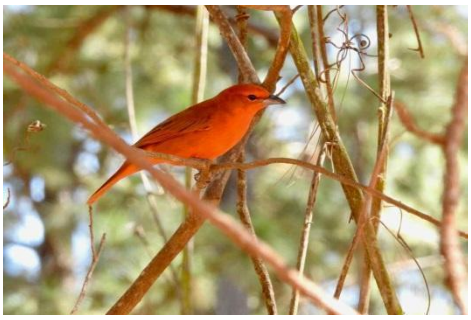

PHOTO ID: 211
Valence mean (95% CI): 6.7 (6.3; 7.1)
Arousal mean (95% CI): 3.4 (3.0; 3.9)

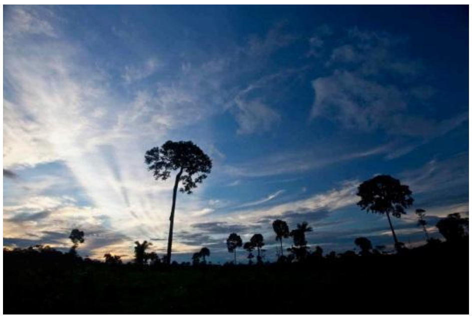

PHOTO ID: 265
Valence mean (95% CI): 7.2 (6.9; 7.5)
Arousal mean (95% CI): 3.9 (3.4; 4.4)

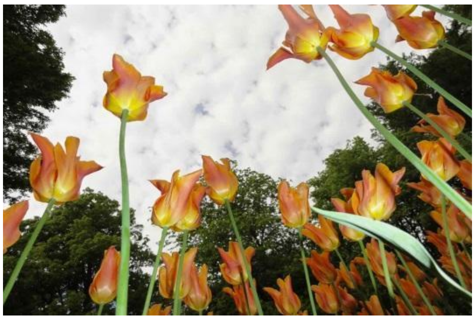

PHOTO ID: 311
Valence mean (95% CI): 7.2 (6.8; 7.6)

Arousal mean (95% CI): 3.9 (3.5; 4.4)

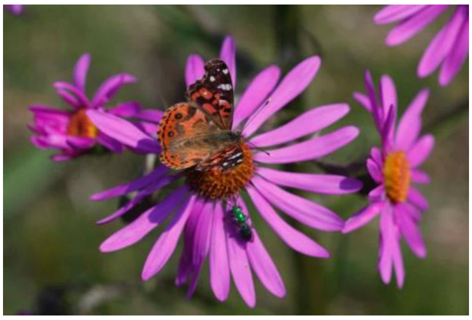

PHOTO ID: 22
Valence mean (95% CI): 7.1 (6.7; 7.5)
Arousal mean (95% CI): 3.9 (3.4; 4.4)

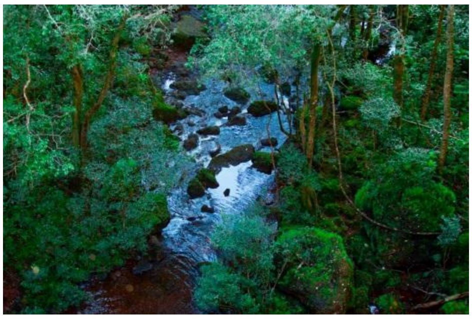

PHOTO ID: 140
Valence mean (95% CI): 7.1 (6.7; 7.5)
Arousal mean (95% CI): 3.9 (3.4; 4.4)

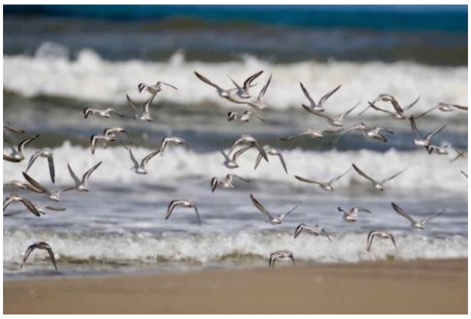

PHOTO ID: 190
Valence mean (95% CI): 7.1 (6.8; 7.5)

Arousal mean (95% CI): 3.9 (3.5; 4.4)

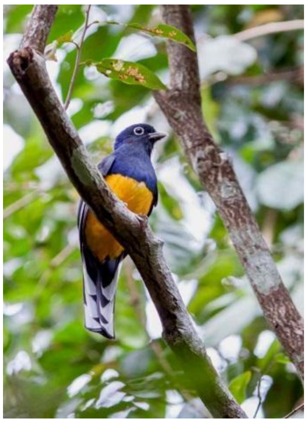

PHOTO ID: 296
Valence mean (95% CI): 7.1 (6.8; 7.4)
Arousal mean (95% CI): 3.9 (3.5; 4.3)

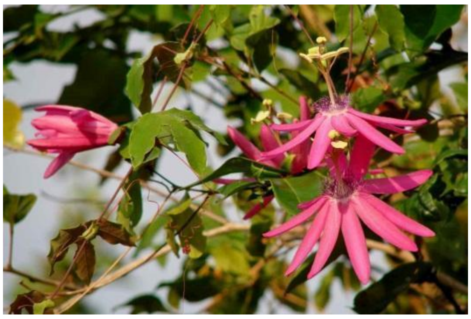

PHOTO ID: 309
Valence mean (95% CI): 7.1 (6.8; 7.5)
Arousal mean (95% CI): 3.9 (3.5; 4.3)

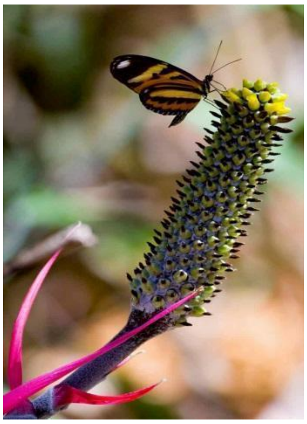

PHOTO ID: 19
Valence mean (95% CI): 6.5 (6.1; 6.9)

Arousal mean (95% CI): 3.3 (2.9; 3.8)

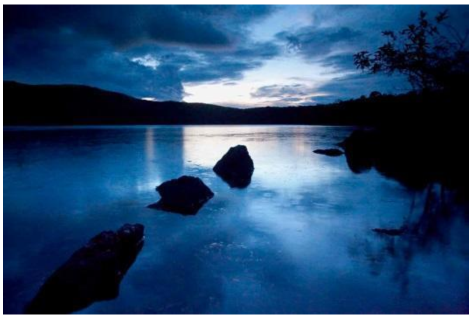

PHOTO ID: 215
Valence mean (95% CI): 6.7 (6.2; 7.2)
Arousal mean (95% CI): 3.5 (3.0; 4.1)

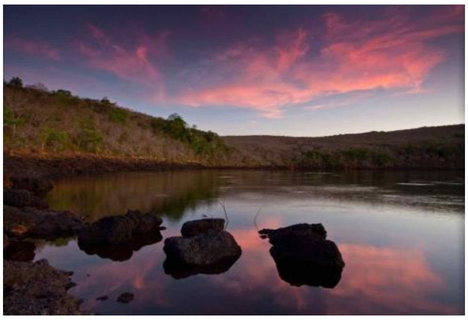

PHOTO ID: 250
Valence mean (95% CI): 6.9 (6.5; 7.3)
Arousal mean (95% CI): 3.7 (3.2; 4.2)

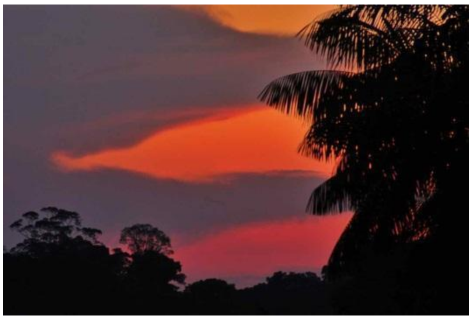

PHOTO ID: 260
Valence mean (95% CI): 6.7 (6.3; 7.0)
Arousal mean (95% CI): 3.5 (3.1; 4.0)

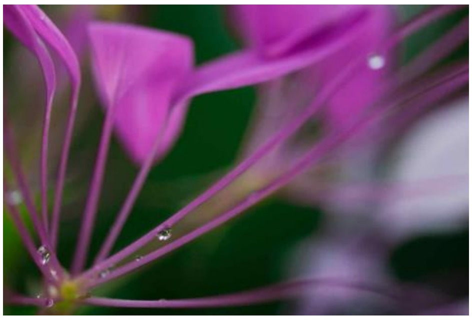

PHOTO ID: 288
Valence mean (95% CI): 7.0 (6.6; 7.4)

Arousal mean (95% CI): 3.8 (3.4; 4.4)

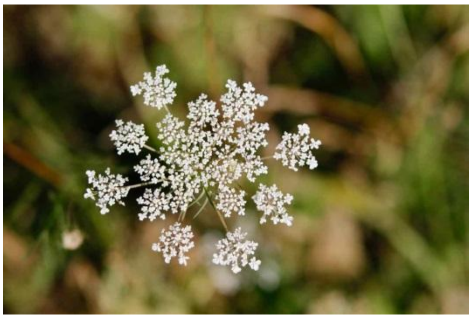

PHOTO ID: 365
Valence mean (95% CI): 7.2 (6.8; 7.6)
Arousal mean (95% CI): 4.0 (3.5; 4.6)

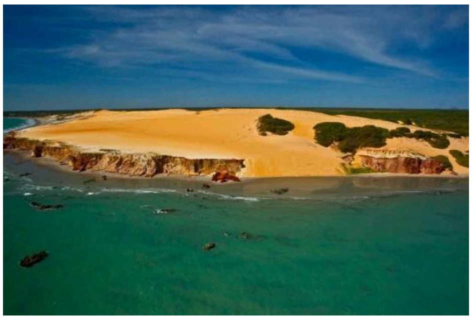

PHOTO ID: 38
Valence mean (95% CI): 6.8 (6.5; 7.2)
Arousal mean (95% CI): 3.7 (3.2; 4.2)

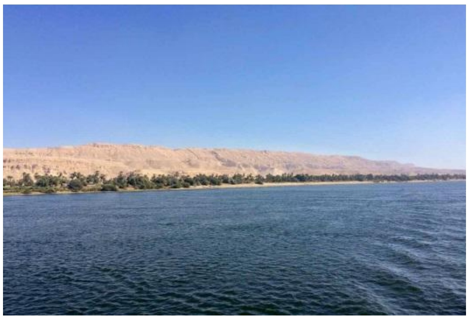

PHOTO ID: 103
Valence mean (95% CI): 6.9 (6.5; 7.3)
Arousal mean (95% CI): 3.8

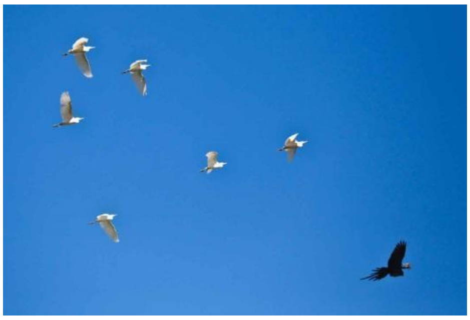

PHOTO ID: 181
Valence mean (95% CI): 7.3 (6.9; 7.7)

Arousal mean (95% CI): 4.2 (3.7; 4.7)

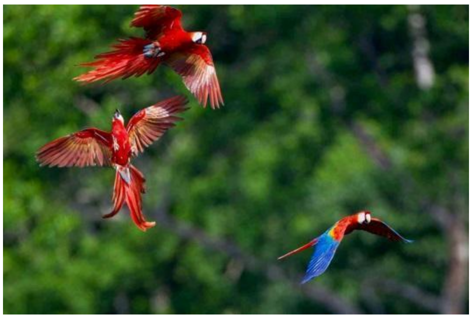

PHOTO ID: 298
Valence mean (95% CI): 6.8 (6.4; 7.1)
Arousal mean (95% CI): 3.7 (3.3; 4.2)

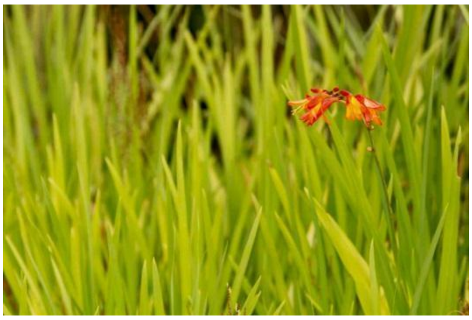

PHOTO ID: 322
Valence mean (95% CI): 6.8 (6.4; 7.2)
Arousal mean (95% CI): 3.7 (3.3; 4.2)

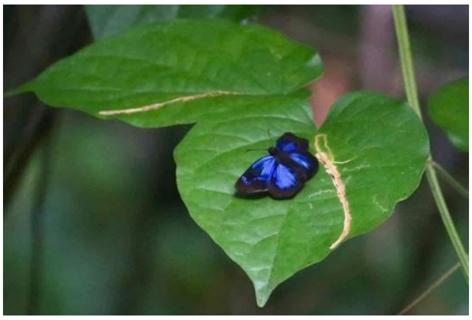

PHOTO ID: 337
Valence mean (95% CI): 6.8 (6.5; 7.2)
Arousal mean (95% CI): 3.7 (3.3; 4.3)

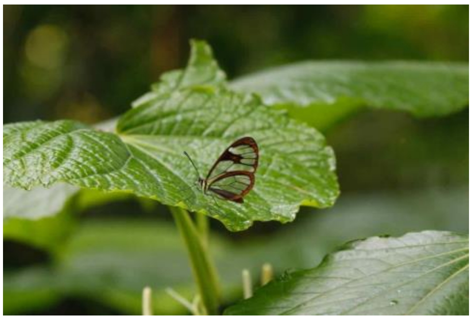

PHOTO ID: 377

Valence mean (95% CI): 7.1 (6.7; 7.5)
Arousal mean (95% CI): 4.0 (3.5; 4.6)

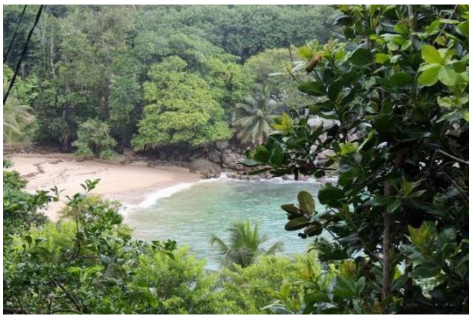

PHOTO ID: 107
Valence mean (95% CI): 7.0 (6.7; 7.4)
Arousal mean (95% CI): 3.9

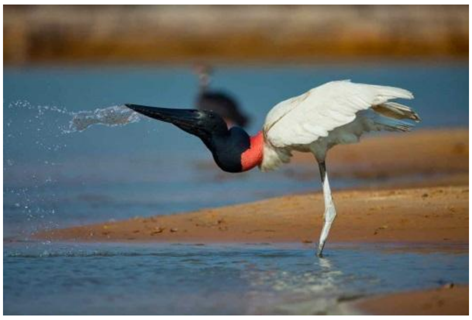

PHOTO ID: 216
Valence mean (95% CI): 7.0 (6.5; 7.4)
Arousal mean (95% CI): 3.9 (3.3; 4.5)

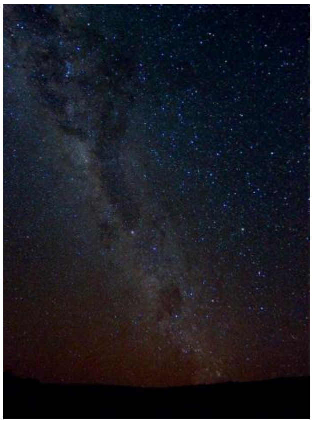

PHOTO ID: 224

Valence mean (95% CI): 7.0 (6.7; 7.3)
Arousal mean (95% CI): 3.9 (3.4; 4.4)

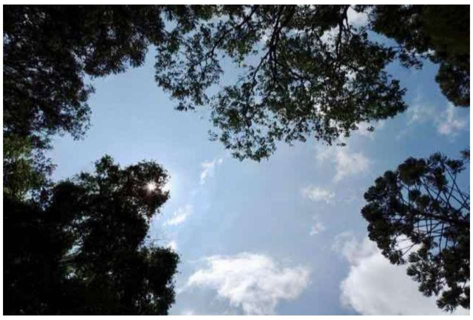

PHOTO ID: 334
Valence mean (95% CI): 7.0 (6.7; 7.3)
Arousal mean (95% CI): 3.9 9 (3.4; 4.4)

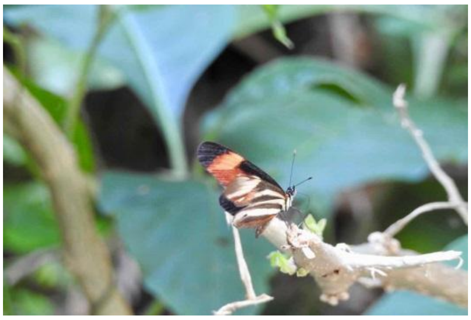

PHOTO ID: 183
Valence mean (95% CI): 7.1 (6.8; 7.5)
Arousal mean (95% CI): 4.1 (3.6; 4.7)

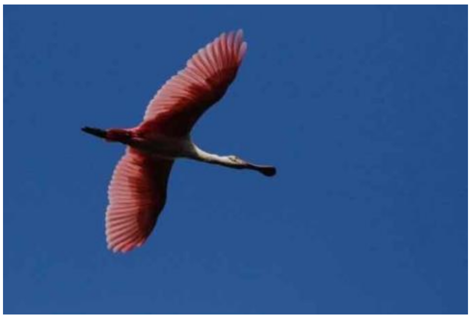

PHOTO ID: 317
Valence mean (95% CI): 7.1 (6.8; 7.4)
Arousal mean (95% CI): 4.1

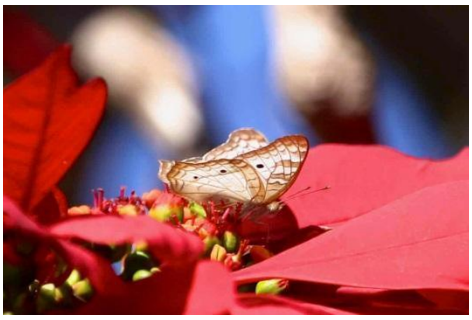

PHOTO ID: 34

Valence mean (95% CI): 6.7 (6.2; 7.2)
Arousal mean (95% CI): 3.7 (3.3; 4.2)

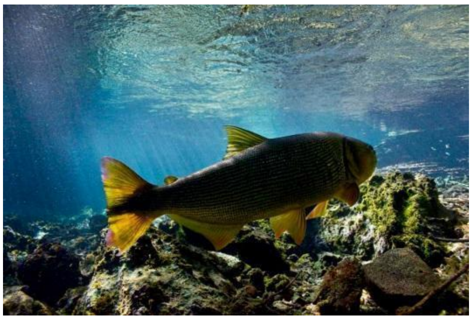

PHOTO ID: 115
Valence mean (95% CI): 7.2 (6.9; 7.5)
Arousal mean (95% CI): 4.2 (3.8; 4.7)

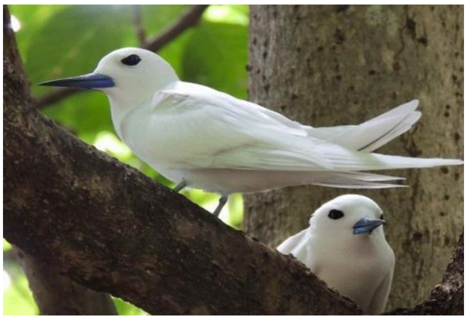

PHOTO ID: 125
Valence mean (95% CI): 6.8 (6.5; 7.2)
Arousal mean (95% CI): 3.8 (3.5; 4.2)

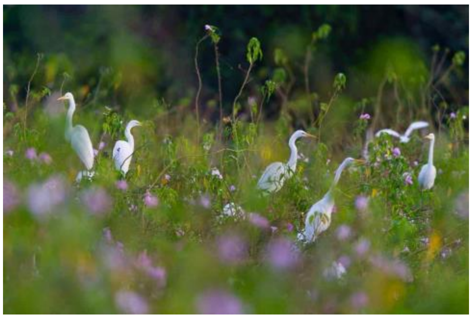

PHOTO ID: 159
Valence mean (95% CI): 7.3 (7.0; 7.6)
Arousal mean (95% CI): 4.3 (3.8; 4.8)

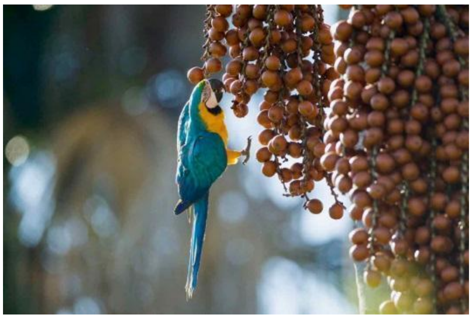

PHOTO ID: 197

Valence mean (95% CI): 7.2 (6.8; 7.5)
Arousal mean (95% CI): 4.2 (3.7; 4.7)

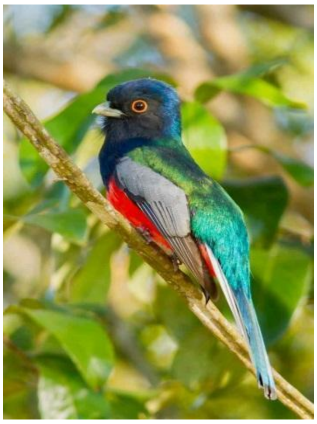

PHOTO ID: 198
Valence mean (95% CI): 7.5 (7.1; 7.8)
Arousal mean (95% CI): 4.5 (4.0; 5.0)

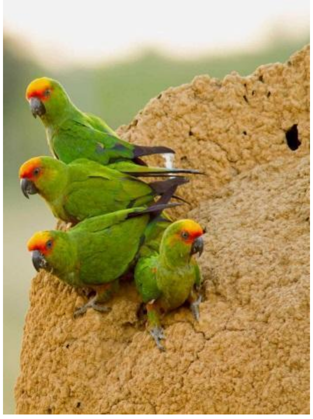

PHOTO ID: 226
Valence mean (95% CI): 6.8 (6.4; 7.2)
Arousal mean (95% CI): 3.8 (3.3; 4.3)

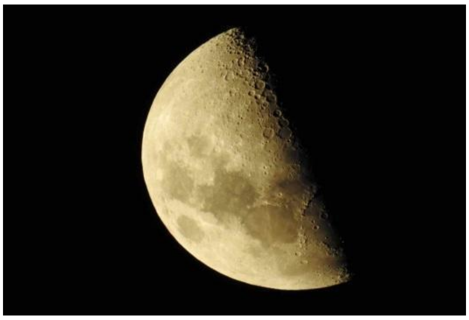

PHOTO ID: 308

Valence mean (95% CI): 7.0 (6.7; 7.3)
Arousal mean (95% CI): 4.0 (3.6; 4.5)

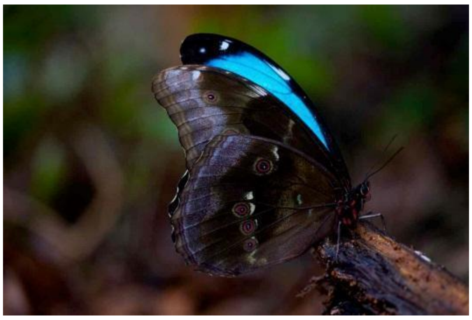

PHOTO ID: 323
Valence mean (95% CI): 6.9 (6.5; 7.2)
Arousal mean (95% CI): 3.9 (3.5; 4.4)

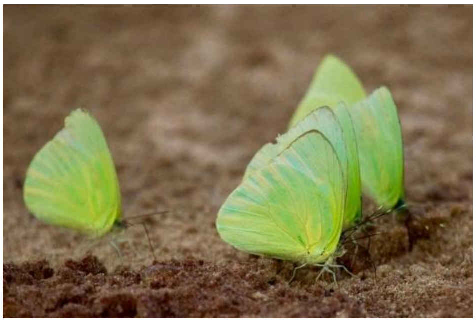

PHOTO ID: 42
Valence mean (95% CI): 7.4 (7.0; 7.9)
Arousal mean (95% CI): 4.4 (3.7; 5.2)

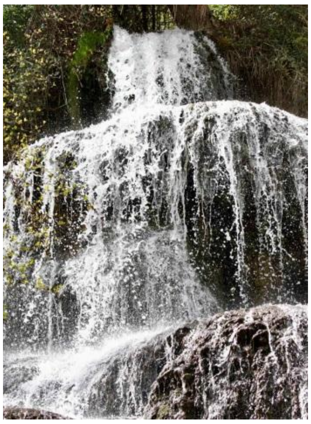

PHOTO ID: 145

Valence mean (95% CI): 7.4 (7.1; 7.7)
Arousal mean (95% CI): 4.4 (3.9; 4.9)

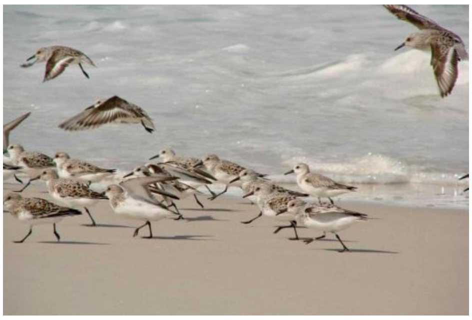

PHOTO ID: 21
Valence mean (95% CI): 7.1 (6.7; 7.4)
Arousal mean (95% CI): 4.2 (3.6; 4.8)

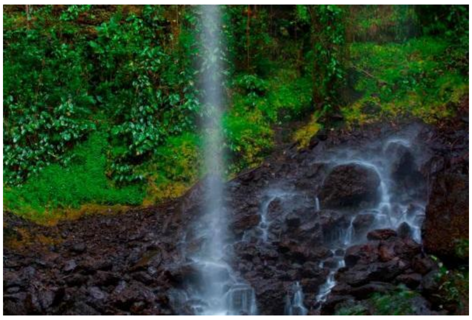

PHOTO ID: 278
Valence mean (95% CI): 7.1 (6.8; 7.4)
Arousal mean (95% CI): 4.2 (3.7; 4.6)

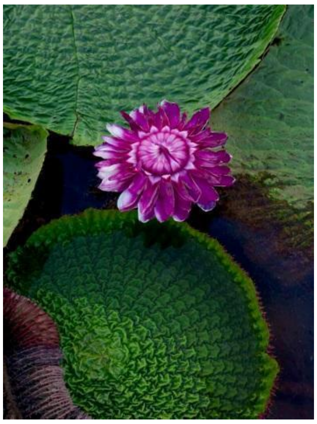

PHOTO ID: 300

Valence mean (95% CI): 7.1 (6.8; 7.5)
Arousal mean (95% CI): 4.2 (3.7; 4.7)

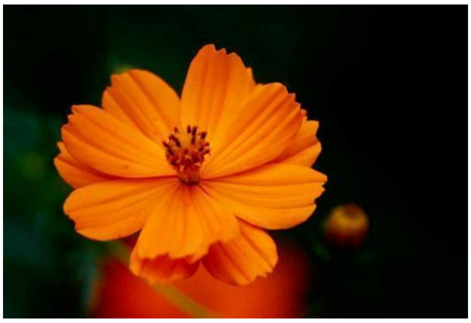

PHOTO ID: 24
Valence mean (95% CI): 6.9 (6.6; 7.3)
Arousal mean (95% CI): 4.0 (3.6; 4.5)

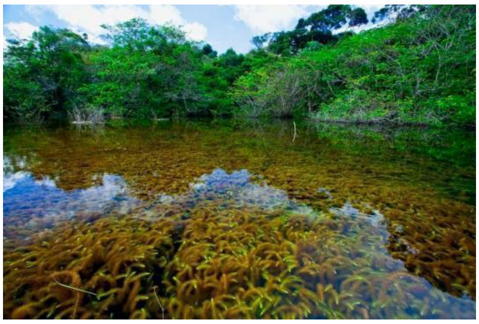

PHOTO ID: 134
Valence mean (95% CI): 6.8 (6.4; 7.2)
Arousal mean (95% CI): 3.9 (3.4; 4.4)

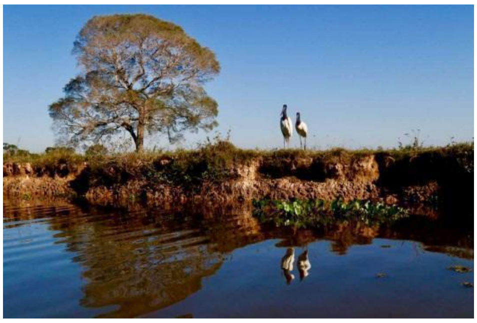

PHOTO ID: 136
Valence mean (95% CI): 6.8 (6.4; 7.2)
Arousal mean (95% CI): 3.9 (3.4; 4.4)

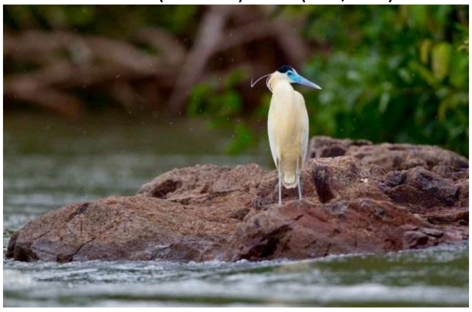

PHOTO ID: 137

Valence mean (95% CI): 7.0 (6.7; 7.4)
Arousal mean (95% CI): 4.1 (3.6; 4.7)

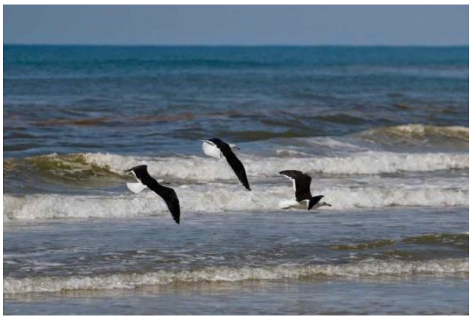

PHOTO ID: 147
Valence mean (95% CI): 6.8 (6.5; 7.2)
Arousal mean (95% CI): 3.9 (3.4; 4.4)

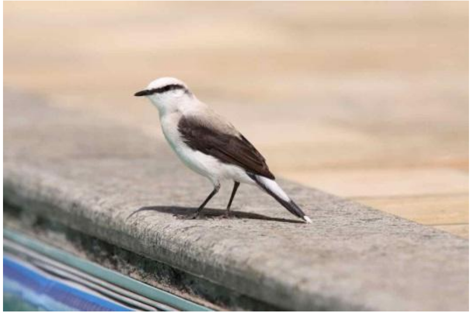

PHOTO ID: 175
Valence mean (95% CI): 7.3 (6.9; 7.7)
Arousal mean (95% CI): 4.4 (3.9; 4.9)

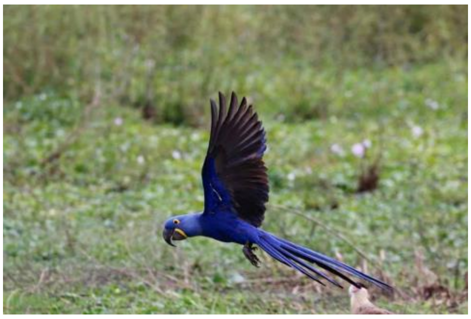

PHOTO ID: 184
Valence mean (95% CI): 6.8 (6.6; 7.1)
Arousal mean (95% CI): 3.9 (3.6; 4.3)

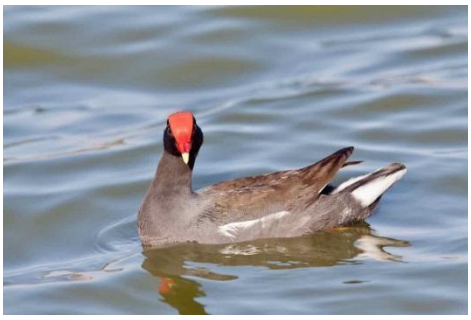

PHOTO ID: 191

Valence mean (95% CI): 7.0 (6.6; 7.4)
Arousal mean (95% CI): 4.1 (3.6; 4.6)

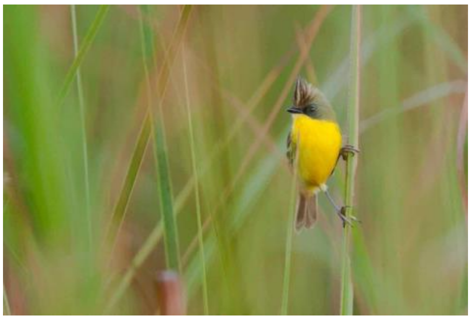

PHOTO ID: 228
Valence mean (95% CI): 6.4 (6.0; 6.8)
Arousal mean (95% CI): 3.5 (3.1; 4.0)

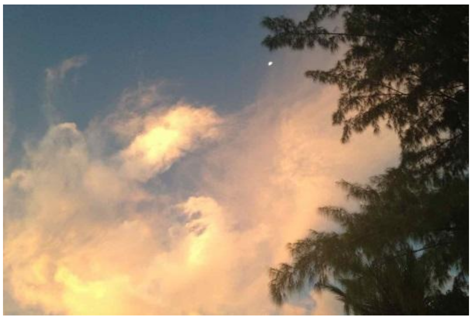

PHOTO ID: 256
Valence mean (95% CI): 6.8 (6.5; 7.2)
Arousal mean (95% CI): 3.9 (3.5; 4.4)

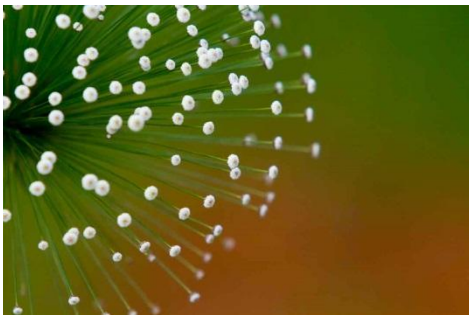

PHOTO ID: 307
Valence mean (95% CI): 6.8 (6.4; 7.3)
Arousal mean (95% CI): 3.9 (3.5; 4.3)

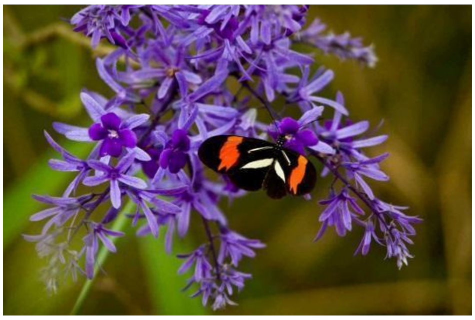

PHOTO ID: 319

Valence mean (95% CI): 6.8 (6.4; 7.1)
Arousal mean (95% CI): 3.9 (3.4; 4.3)

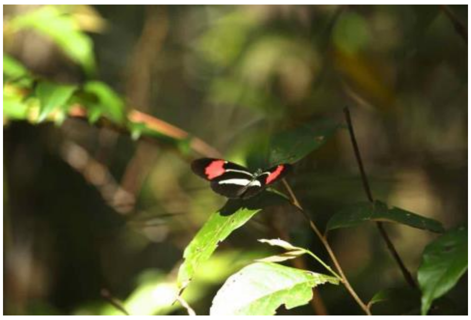

PHOTO ID: 41
Valence mean (95% CI): 6.6 (6.3; 7.0)
Arousal mean (95% CI): 3.7 (3.2; 4.2)

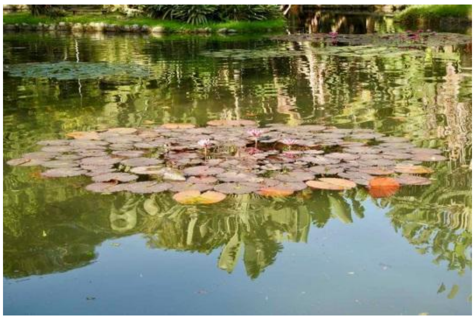

PHOTO ID: 214
Valence mean (95% CI): 6.7 (6.3; 7.2)
Arousal mean (95% CI): 3.8 (3.3; 4.3)

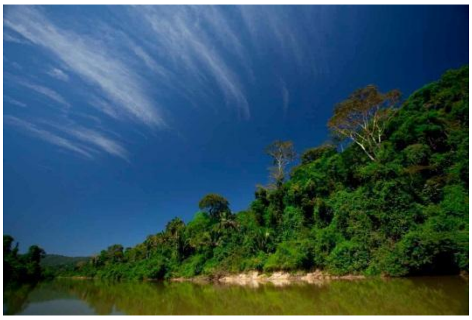

PHOTO ID: 264
Valence mean (95% CI): 7.2 (6.8; 7.6)
Arousal mean (95% CI): 4.3 (3.7; 4.9)

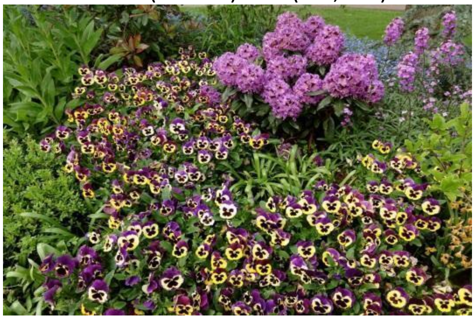

PHOTO ID: 267

Valence mean (95% CI): 6.7 (6.5; 7.0)
Arousal mean (95% CI): 3.8 (3.5; 4.2)

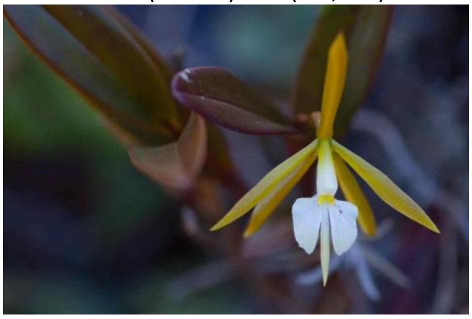

PHOTO ID: 47
Valence mean (95% CI): 7.0 (6.6; 7.3)
Arousal mean (95% CI): 4.2 (3.7; 4.7)

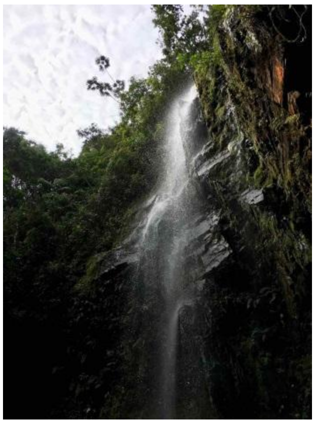

PHOTO ID: 105
Valence mean (95% CI): 7.0 (6.7; 7.3)
Arousal mean (95% CI): 4.2 (3.8; 4.6)

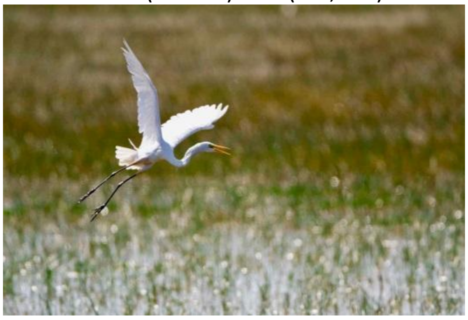

PHOTO ID: 131
Valence mean (95% CI): 6.9 (6.6; 7.2)
Arousal mean (95% CI): 4.1 (3.6; 4.6)

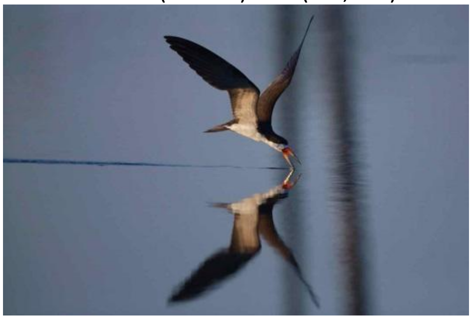

PHOTO ID: 155

Valence mean (95% CI): 7.0 (6.7; 7.4)
Arousal mean (95% CI): 4.2 (3.8; 4.7)

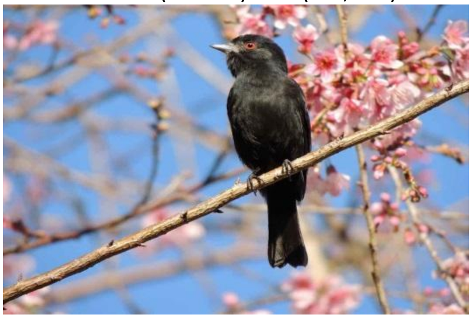

PHOTO ID: 157
Valence mean (95% CI): 7.1 (6.8; 7.5)
Arousal mean (95% CI): 4.3 (3.8; 4.8)

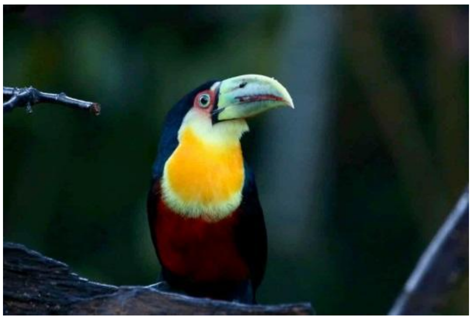

PHOTO ID: 161
Valence mean (95% CI): 6.9 (6.5; 7.3)
Arousal mean (95% CI): 4.1 (3.7; 4.5)

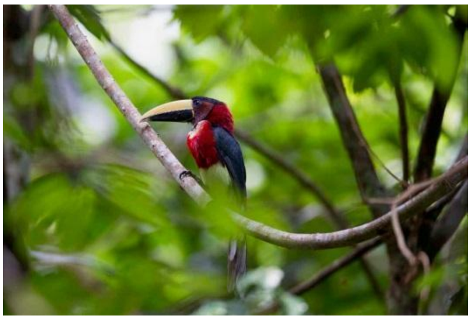

PHOTO ID: 170
Valence mean (95% CI): 7.1 (6.8; 7.4)
Arousal mean (95% CI): 4.3 (3.9; 4.8)

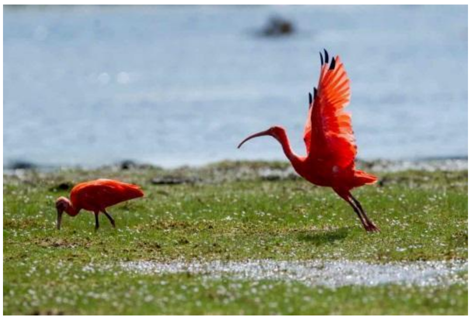

PHOTO ID: 232

Valence mean (95% CI): 6.5 (6.1; 6.9)
Arousal mean (95% CI): 3.7 (3.2; 4.2)

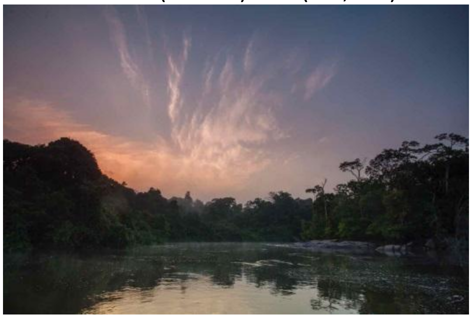

PHOTO ID: 236
Valence mean (95% CI): 6.6 (6.2; 6.9)
Arousal mean (95% CI): 3.8 (3.3; 4.3)

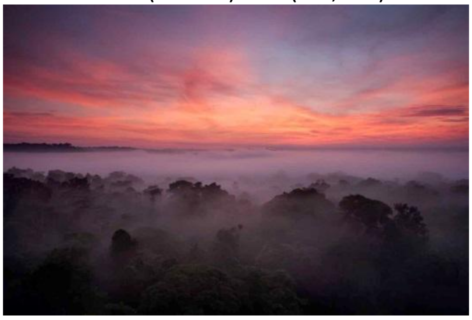

PHOTO ID: 270
Valence mean (95% CI): 6.8 (6.5; 7.1)
Arousal mean (95% CI): 4.0 (3.5; 4.4)

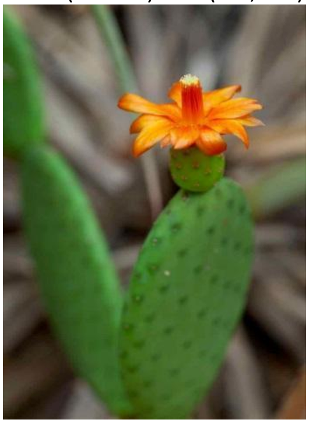

PHOTO ID: 277
Valence mean (95% CI): 6.5 (6.1; 7.0)
Arousal mean (95% CI): 3.7 (3.2; 4.2)

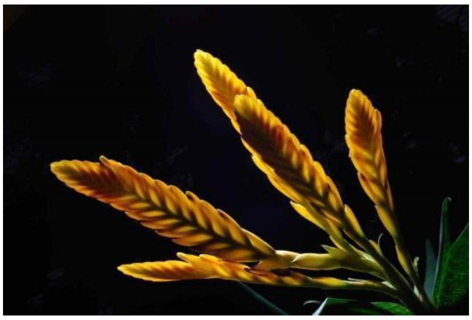

PHOTO ID: 324
Valence mean (95% CI): 7.0 (6.7; 7.3)
Arousal mean (95% CI): 4.2 (3.8; 4.7)

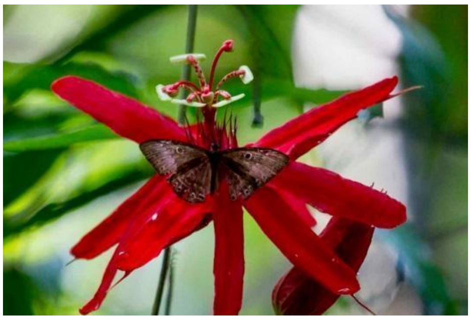

PHOTO ID: 327
Valence mean (95% CI): 7.0 (6.6; 7.4)
Arousal mean (95% CI): 4.2 (3.7; 4.7)

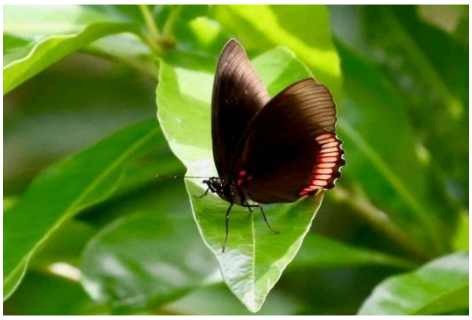

PHOTO ID: 392
Valence mean (95% CI): 6.8 (6.3; 7.2)
Arousal mean (95% CI): 4.0 (3.4; 4.7)

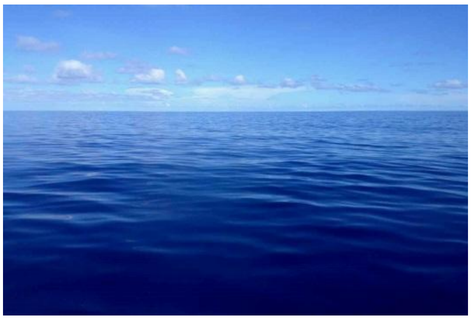

PHOTO ID: 248
Valence mean (95% CI): 6.7 (6.3; 7.0)
Arousal mean (95% CI): 3.9 (3.5; 4.4)

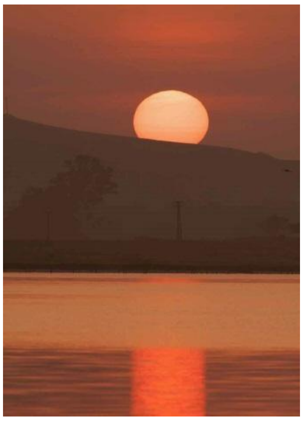

PHOTO ID: 273
Valence mean (95% CI): 6.7 (6.2; 7.1)
Arousal mean (95% CI): 3.9 (3.4; 4.4)

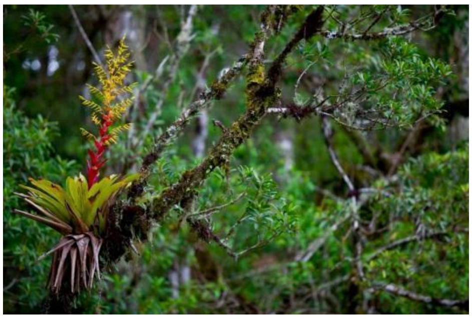

PHOTO ID: 16
Valence mean (95% CI): 7.3 (7.0; 7.7)
Arousal mean (95% CI): 4.6 (4.0; 5.1)

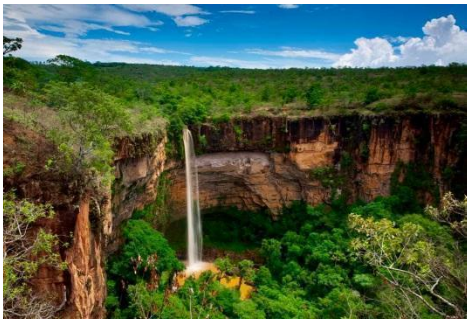

PHOTO ID: 64
Valence mean (95% CI): 6.8 (6.5; 7.0)
Arousal mean (95% CI): 4.1 (3.7; 4.6)

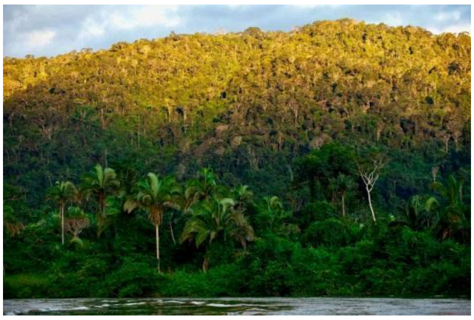

PHOTO ID: 193
Valence mean (95% CI): 7.1 (6.8; 7.5)
Arousal mean (95% CI): 4.4 (3.9; 5.0)

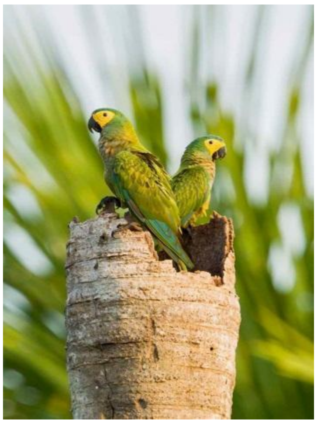

PHOTO ID: 255
Valence mean (95% CI): 6.8 (6.5; 7.2)
Arousal mean (95% CI): 4.1 (3.7; 4.5)

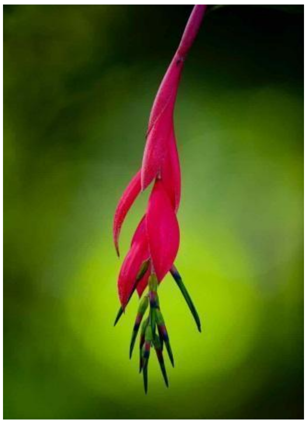

PHOTO ID: 79

Valence mean (95% CI): 6.7 (6.4; 7.0)
Arousal mean (95% CI): 4.0 (3.6; 4.4)

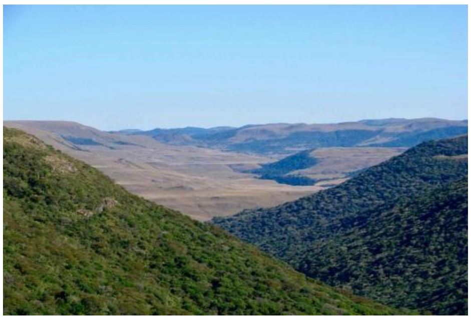

PHOTO ID: 97
Valence mean (95% CI): 6.5 (6.2; 6.8)
Arousal mean (95% CI): 3.8 (3.4; 4.2)

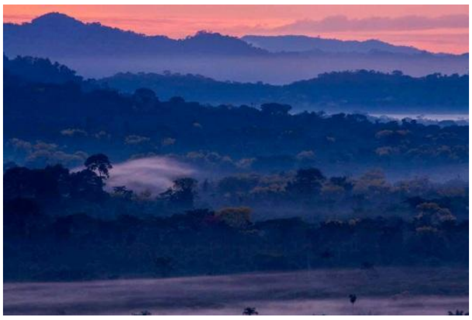

PHOTO ID: 98
Valence mean (95% CI): 6.9 (6.6; 7.3)
Arousal mean (95% CI): 4.2 (3.8; 4.7)

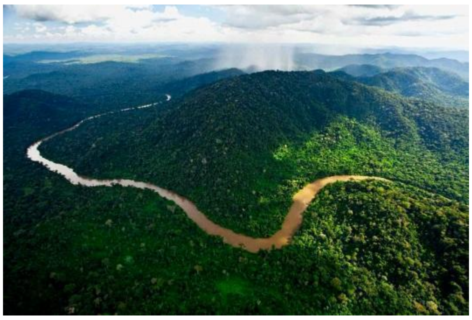

PHOTO ID: 120
Valence mean (95% CI): 6.7 (6.3; 7.0)
Arousal mean (95% CI): 4.0 (3.5; 4.6)

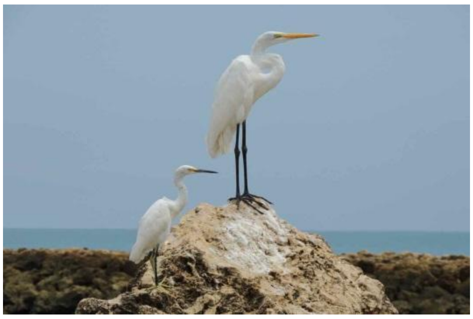

PHOTO ID: 162

Valence mean (95% CI): 7.0 (6.7; 7.3)
Arousal mean (95% CI): 4.3 (3.8; 4.8)

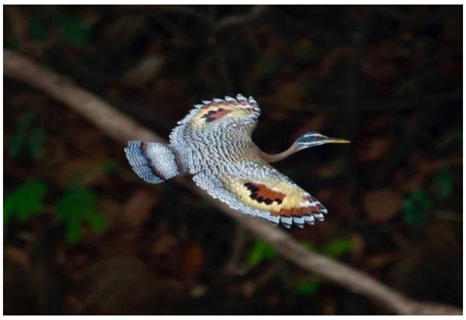

PHOTO ID: 171
Valence mean (95% CI): 7.0 (6.6; 7.4)
Arousal mean (95% CI): 4.3 (3.8; 4.8)

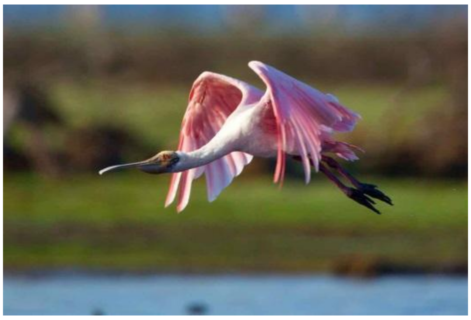

PHOTO ID: 189
Valence mean (95% CI): 6.9 (6.6; 7.4)
Arousal mean (95% CI): 4.2 (3.7; 4.8)

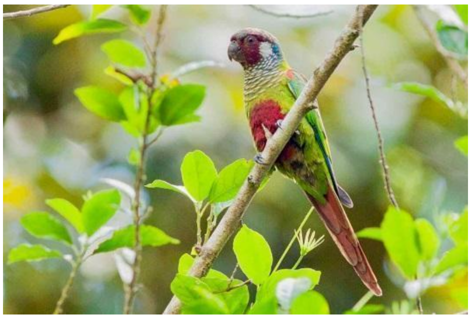

PHOTO ID: 218
Valence mean (95% CI): 6.7 (6.4; 7.0)
Arousal mean (95% CI): 4.0 (3.5; 4.6)

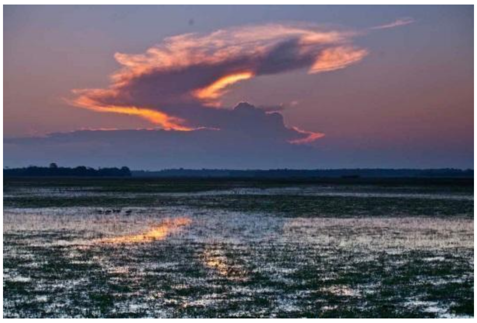

PHOTO ID: 314
Valence mean (95% CI): 6.4 (6.0; 6.8)
Arousal mean (95% CI): 3.7 (3.2; 4.2)

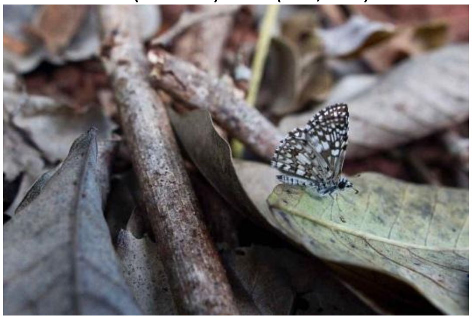

PHOTO ID: 249
Valence mean (95% CI): 6.9 (6.5; 7.4)
Arousal mean (95% CI): 4.3 (3.8; 4.9)

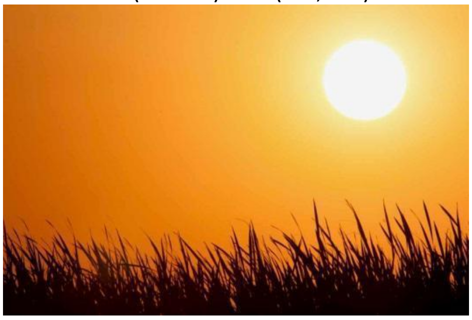

PHOTO ID: 380
Valence mean (95% CI): 7.0 (6.6; 7.4)
Arousal mean (95% CI): 4.3 (3.8; 4.9)

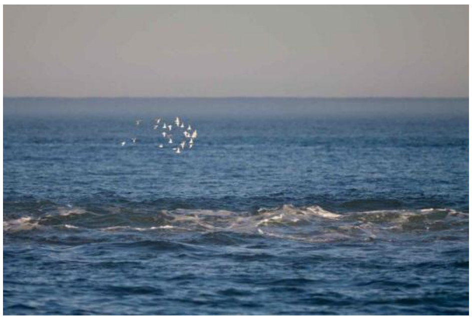

PHOTO ID: 75
Valence mean (95% CI): 6.6 (6.4; 6.9)
Arousal mean (95% CI): 4.0 (3.7; 4.4)

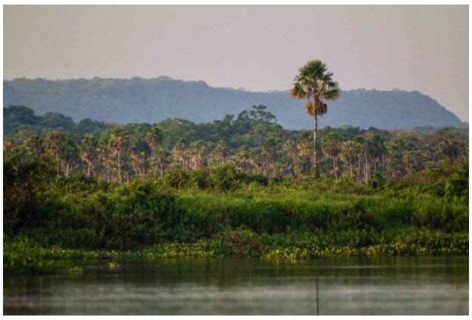

PHOTO ID: 13
Valence mean (95% CI): 6.8 (6.4; 7.2)
Arousal mean (95% CI): 4.2 (3.7; 4.7)

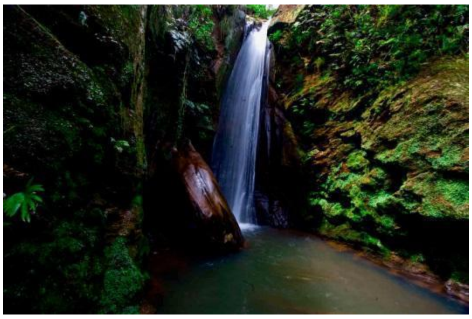

PHOTO ID: 81
Valence mean (95% CI): 6.6 (6.3; 7.0)
Arousal mean (95% CI): 4.0 (3.6; 4.5)

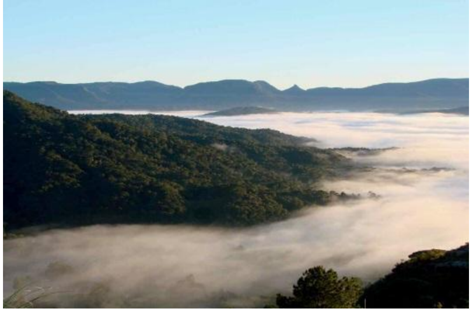

PHOTO ID: 233
Valence mean (95% CI): 6.8 (6.4; 7.2)
Arousal mean (95% CI): 4.2 (3.6; 4.9)

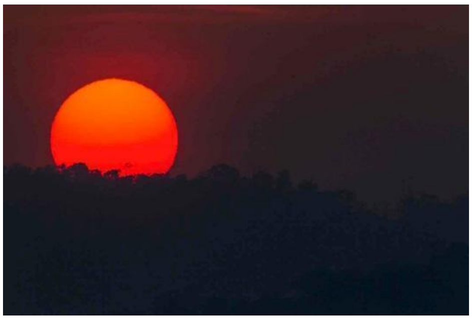

PHOTO ID: 92
Valence mean (95% CI): 6.7 (6.4; 7.0)
Arousal mean (95% CI): 4.1 (3.7; 4.5)

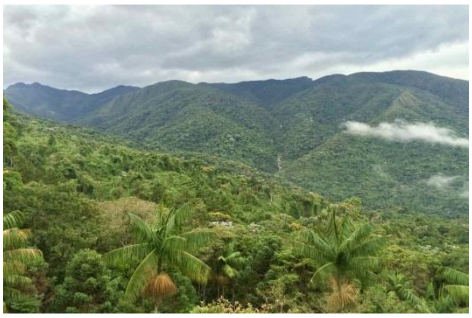

PHOTO ID: 95

Valence mean (95% CI): 6.7 (6.4; 7.1)
Arousal mean (95% CI): 4.1 (3.7; 4.5)

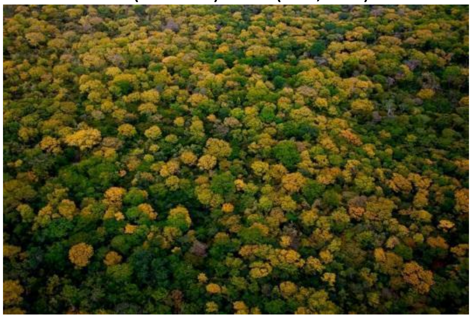

PHOTO ID: 130

Valence mean (95% CI): 6.6 (6.2; 7.1)
Arousal mean (95% CI): 4.0 (3.6; 4.5)

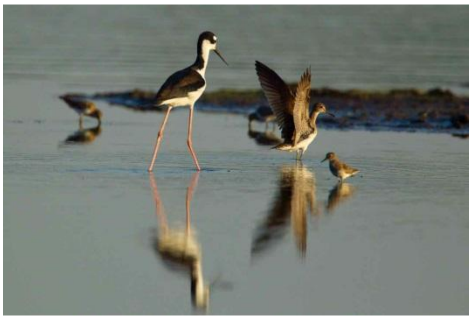

PHOTO ID: 146

Valence mean (95% CI): 6.6 (6.1; 7.0)
Arousal mean (95% CI): 4.0 (3.5; 4.6)

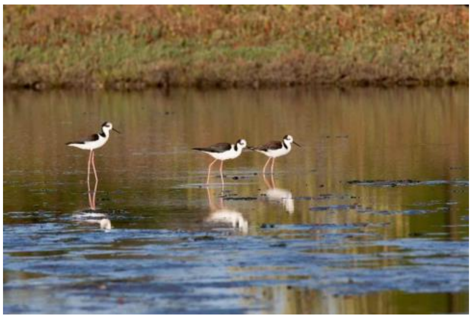

PHOTO ID: 225

Valence mean (95% CI): 6.7 (6.3; 7.1)
Arousal mean (95% CI): 4.1 (3.6; 4.7)

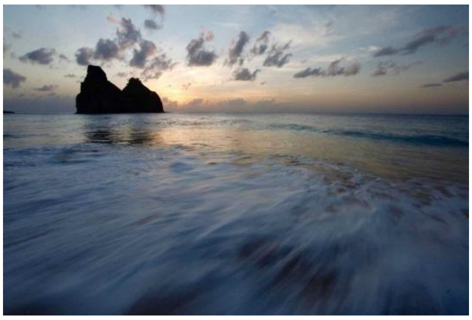

PHOTO ID: 292

Valence mean (95% CI): 6.2 (5.8; 6.7)
Arousal mean (95% CI): 3.6 (3.1; 4.0)

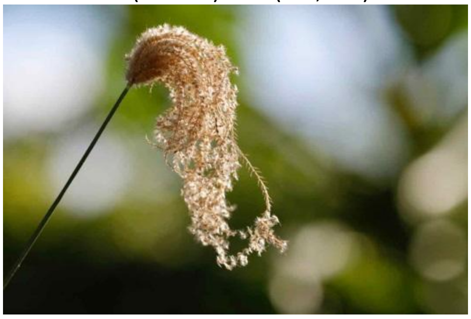

PHOTO ID: 406

Valence mean (95% CI): 6.7 (6.4; 7.1)
Arousal mean (95% CI): 4.1 (3.6; 4.6)

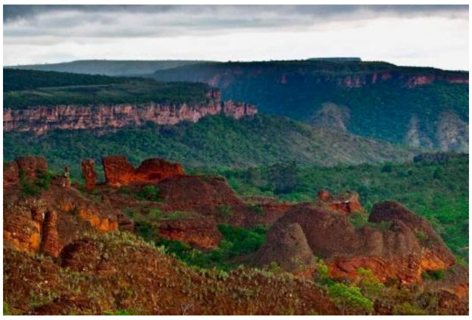

PHOTO ID: 20

Valence mean (95% CI): 7.4 (7.0; 7.7)
Arousal mean (95% CI): 4.8 (4.2; 5.5)

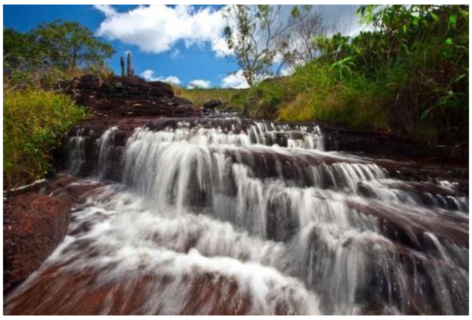

PHOTO ID: 135

Valence mean (95% CI): 7.1 (6.7; 7.4)
Arousal mean (95% CI): 4.6 (4.0; 5.2)

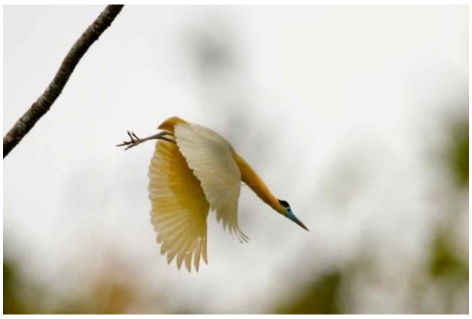

PHOTO ID: 251

Valence mean (95% CI): 7.1 (6.7; 7.5)
Arousal mean (95% CI): 4.6 (4.1; 5.2)

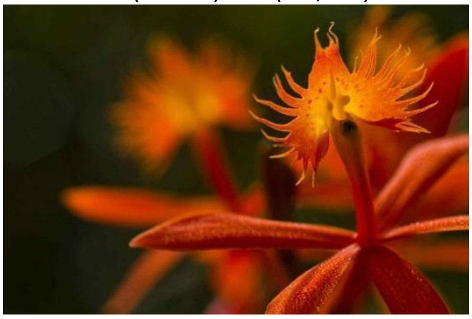

PHOTO ID: 269
Valence mean (95% CI): 7.1 (6.7; 7.4)
Arousal mean (95% CI): 4.6 (4.0; 5.2)

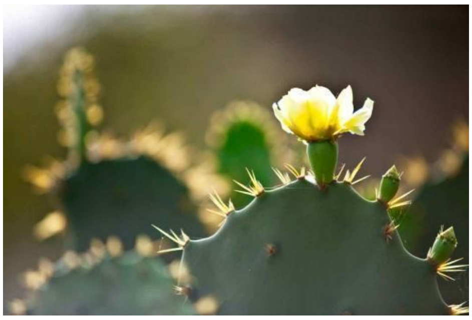

PHOTO ID: 6
Valence mean (95% CI): 6.4 (6.0; 6.9)
Arousal mean (95% CI): 3.9 (3.4; 4.4)

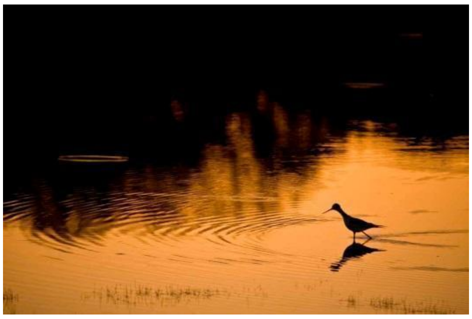

PHOTO ID: 57
Valence mean (95% CI): 7.0 (6.7; 7.3)
Arousal mean (95% CI): 4.5 (4.1; 5.0)

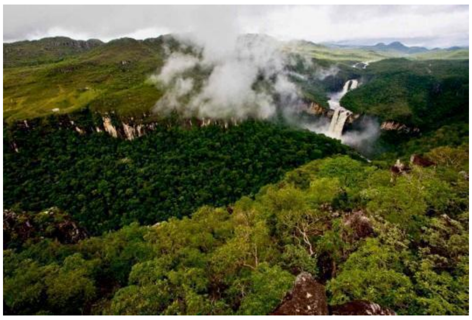

PHOTO ID: 60

Valence mean (95% CI): 6.9 (6.6; 7.1)
Arousal mean (95% CI): 4.4 (4.0; 4.9)

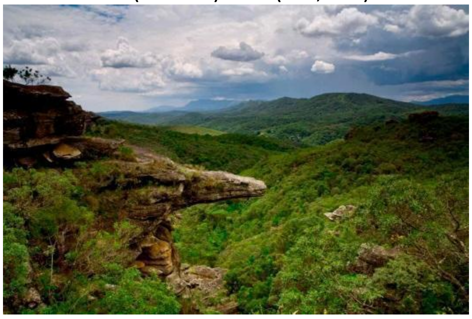

PHOTO ID: 67
Valence mean (95% CI): 6.7 (6.4; 7.0)
Arousal mean (95% CI): 4.2 (3.8; 4.6)

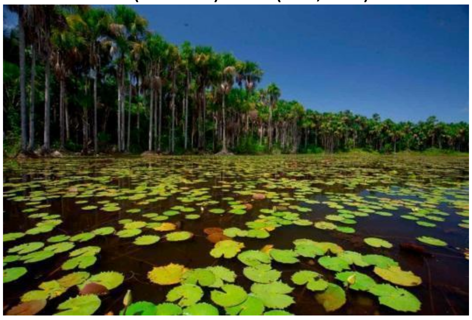

PHOTO ID: 87
Valence mean (95% CI): 6.8 (6.5; 7.1)
Arousal mean (95% CI): 4.3 (4.0; 4.7)

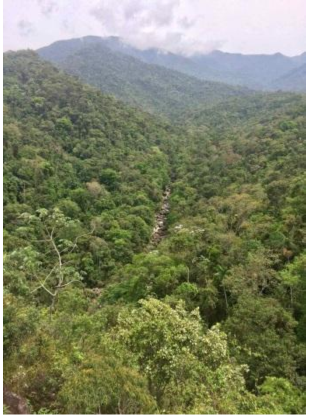

PHOTO ID: 106
Valence mean (95% CI): 7.0 (6.7; 7.4)
Arousal mean (95% CI): 4.5 (4.1; 5.1)

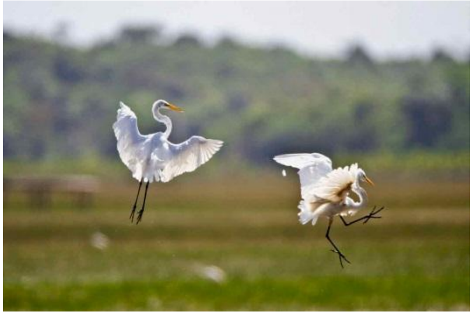

PHOTO ID: 126
Valence mean (95% CI): 6.5 (6.4; 7.2)
Arousal mean (95% CI): 4.0 (3.3; 4.3)

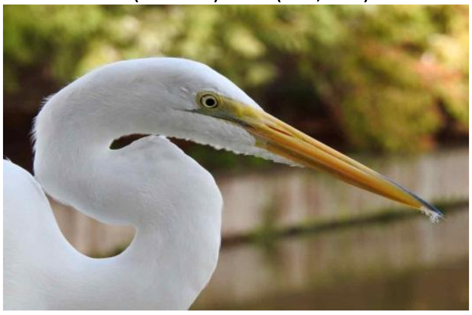

PHOTO ID: 132
Valence mean (95% CI): 6.7 (6.1; 6.9)
Arousal mean (95% CI): 4.2 (3.2; 4.2)

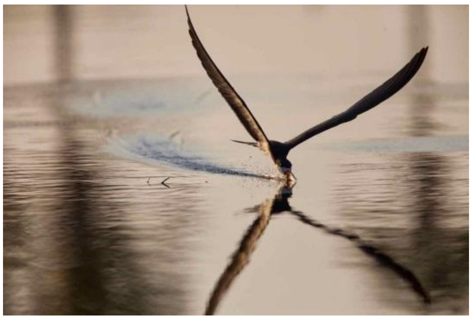

PHOTO ID: 219
Valence mean (95% CI): 6.4 (5.9; 6.8)
Arousal mean (95% CI): 3.9 (3.4; 4.4)

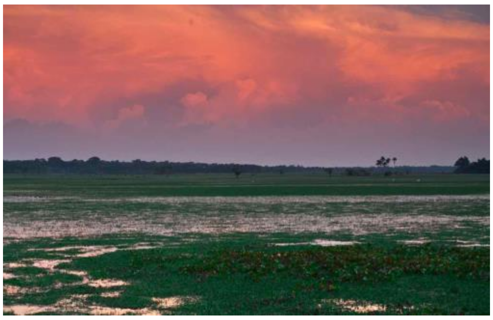

PHOTO ID: 326
Valence mean (95% CI): 6.7 (6.3; 7.0)
Arousal mean (95% CI): 4.2 (3.7; 4.8)

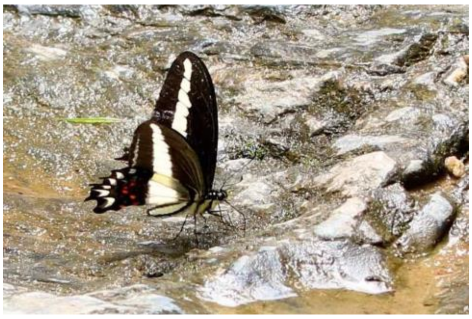

PHOTO ID: 3

Valence mean (95% CI): 6.4 (6.0; 6.9)
Arousal mean (95% CI): 4.0 (3.5; 4.5)

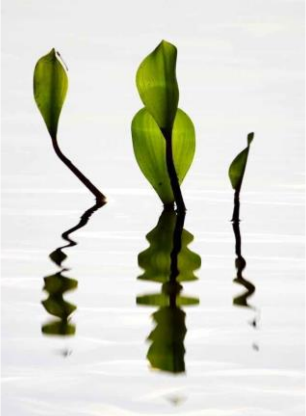

PHOTO ID: 52
Valence mean (95% CI): 6.4 (6.0; 6.8)
Arousal mean (95% CI): 4.0 (3.5; 4.6)

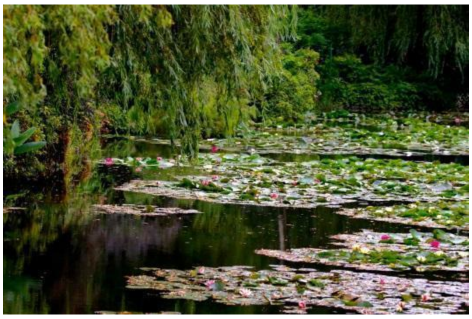

PHOTO ID: 68
Valence mean (95% CI): 6.4 (6.2; 6.7)
Arousal mean (95% CI): 4.0 (3.7; 4.4)

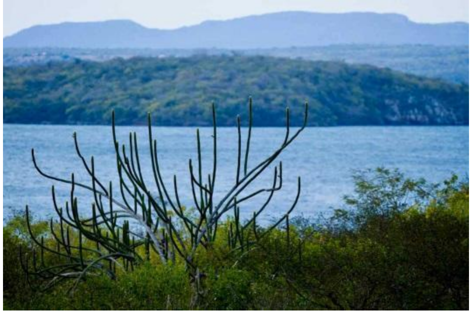

PHOTO ID: 71

Valence mean (95% CI): 7.0 (6.7; 7.3)
Arousal mean (95% CI): 4.6 (4.1; 5.1)

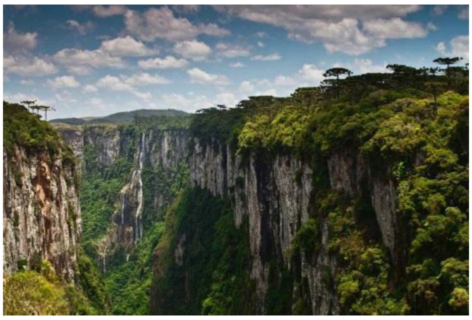

PHOTO ID: 80
Valence mean (95% CI): 6.9 (6.6; 7.1)
Arousal mean (95% CI): 4.5 (4.1; 4.9)

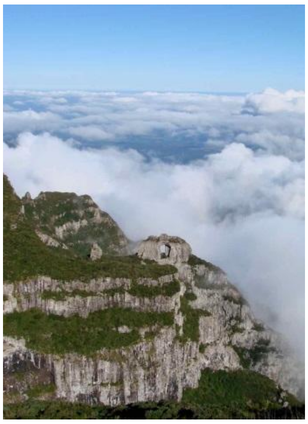

PHOTO ID: 90
Valence mean (95% CI): 6.5 (6.1; 6.8)
Arousal mean (95% CI): 4.1 (3.7; 4.6)

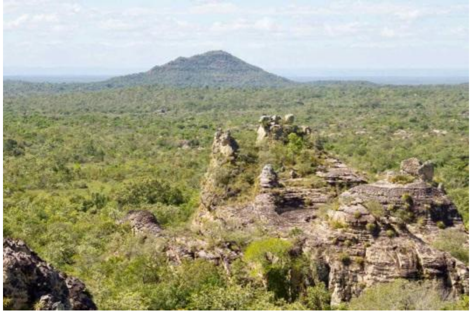

PHOTO ID: 104

Valence mean (95% CI): 6.5 (6.1; 7.0)
Arousal mean (95% CI): 4.1 (3.6; 4.7)

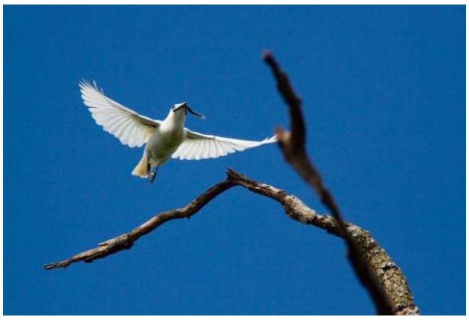

PHOTO ID: 208
Valence mean (95% CI): 6.4 (5.9; 6.9)
Arousal mean (95% CI): 4.0 (3.5; 4.6)

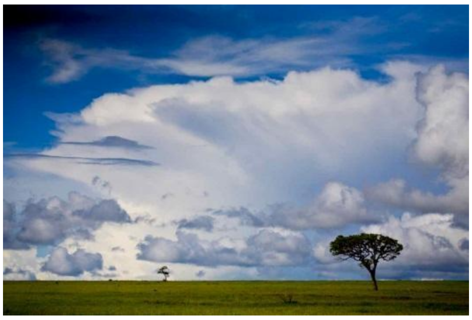

PHOTO ID: 221
Valence mean (95% CI): 6.3 (5.9; 6.7)
Arousal mean (95% CI): 3.9 (3.4; 4.4)

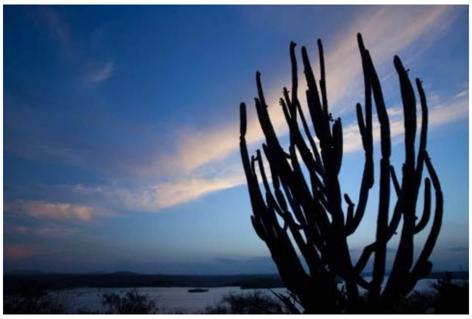

PHOTO ID: 340
Valence mean (95% CI): 6.3 (5.8; 6.8)
Arousal mean (95% CI): 3.9 (3.4; 4.5)

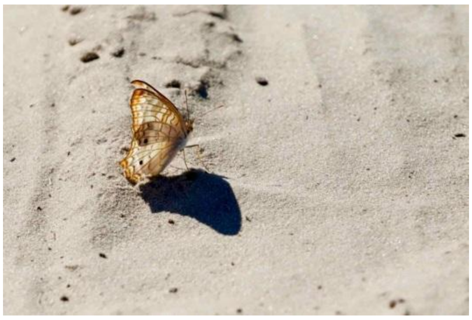

PHOTO ID: 69

Valence mean (95% CI): 6.6 (6.3; 6.9)
Arousal mean (95% CI): 4.2 (3.8; 4.7)

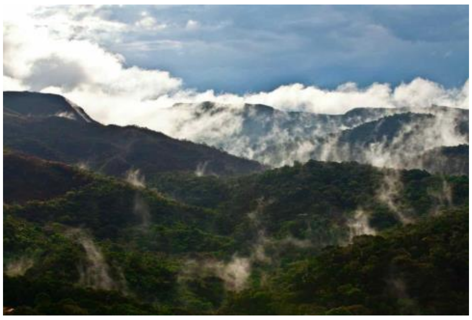

PHOTO ID: 72
Valence mean (95% CI): 6.6 (6.3; 6.9)
Arousal mean (95% CI): 4.2 (3.9; 4.6)

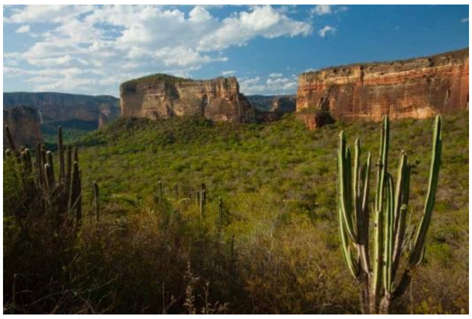

PHOTO ID: 63
Valence mean (95% CI): 6.4 (6.1; 6.8)
Arousal mean (95% CI): 4.1 (3.7; 4.5)

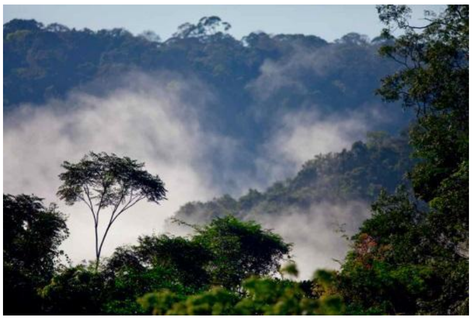

PHOTO ID: 143
Valence mean (95% CI): 6.9 (6.5; 7.3)
Arousal mean (95% CI): 4.6 (4.2; 5.2)

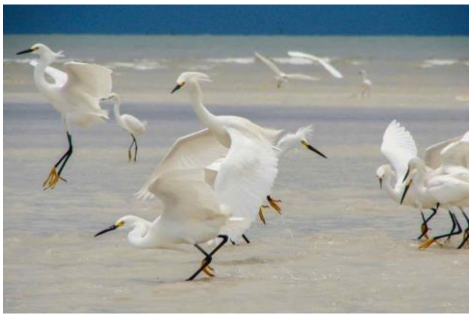

PHOTO ID: 148

Valence mean (95% CI): 6.3 (5.9; 6.8)
Arousal mean (95% CI): 4.0 (3.5; 4.6)

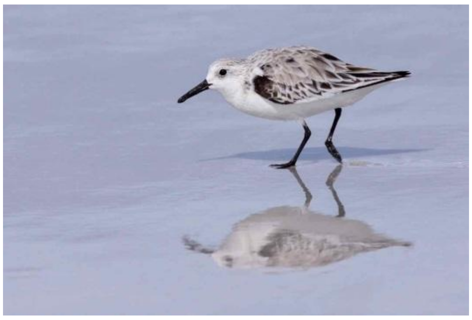

PHOTO ID: 199
Valence mean (95% CI): 7.1 (6.7; 7.5)
Arousal mean (95% CI): 4.8 (4.2; 5.4)

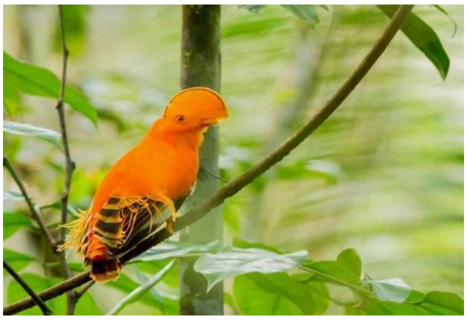

PHOTO ID: 207
Valence mean (95% CI): 6.5 (6.0; 6.9)
Arousal mean (95% CI): 4.2 (3.7; 4.7)

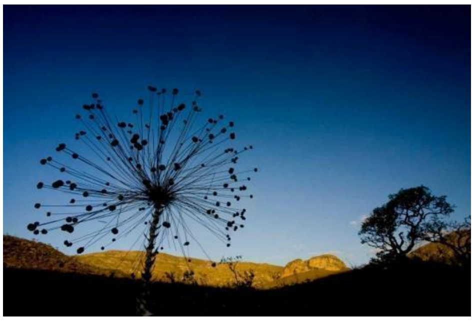

PHOTO ID: 222
Valence mean (95% CI): 6.6 (6.1; 7.1)
Arousal mean (95% CI): 4.3 (3.7; 4.9)

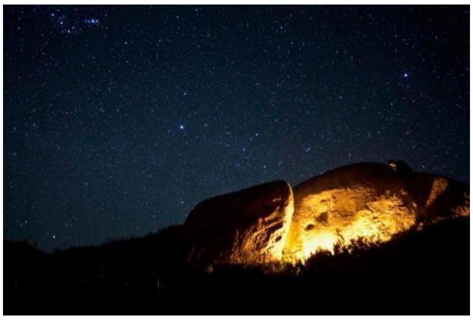

PHOTO ID: 247

Valence mean (95% CI): 6.1 (5.6; 6.6)
Arousal mean (95% CI): 3.8 (3.3; 4.4)

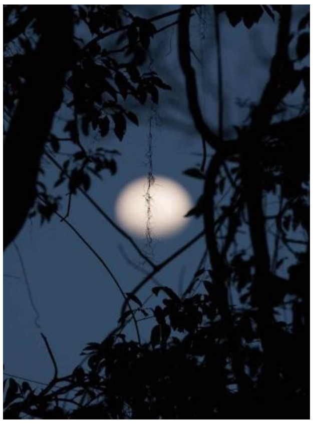

PHOTO ID: 325
Valence mean (95% CI): 6.5 (6.1; 7.0)
Arousal mean (95% CI): 4.2 (3.8; 4.8)

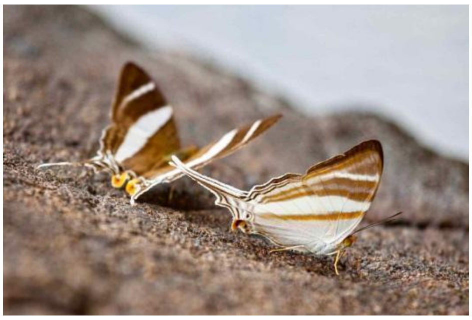

PHOTO ID: 180
Valence mean (95% CI): 6.7 (6.3; 7.1)
Arousal mean (95% CI): 4.4 (3.9; 4.9)

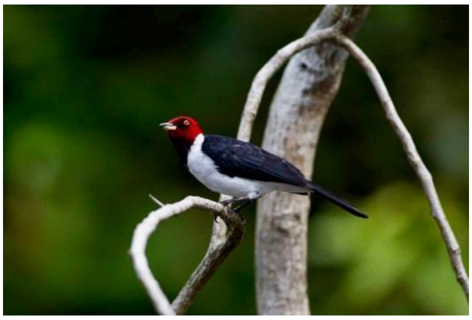

PHOTO ID: 40

Valence mean (95% CI): 6.3 (5.9; 6.7)
Arousal mean (95% CI): 4.1 (3.6; 4.8)

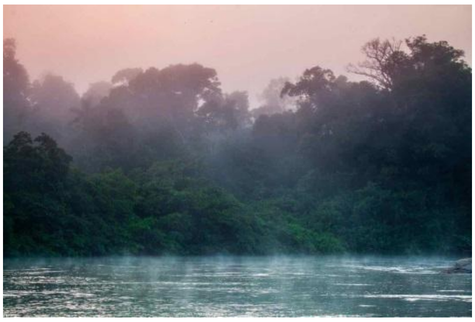

PHOTO ID: 142
Valence mean (95% CI): 7.1 (6.8; 7.4)
Arousal mean (95% CI): 4.9 (4.3; 5.5)

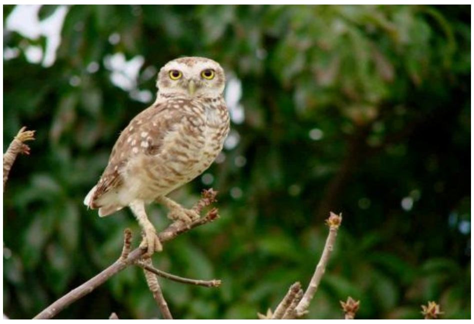

PHOTO ID: 192
Valence mean (95% CI): 6.8 (6.4; 7.1)
Arousal mean (95% CI): 4.6 (4.1; 5.1)

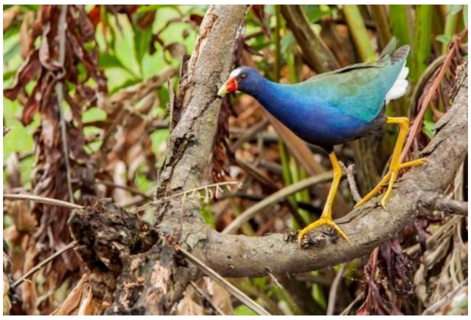

PHOTO ID: 4
Valence mean (95% CI): 6.5 (6.0; 6.9)
Arousal mean (95% CI): 4.3 (3.9; 4.9)

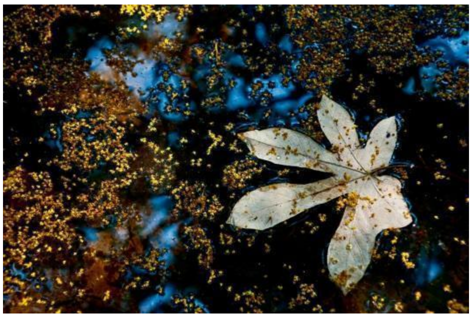

PHOTO ID: 12

Valence mean (95% CI): 6.5 (6.1; 6.9)
Arousal mean (95% CI): 4.3 (3.7; 4.9)

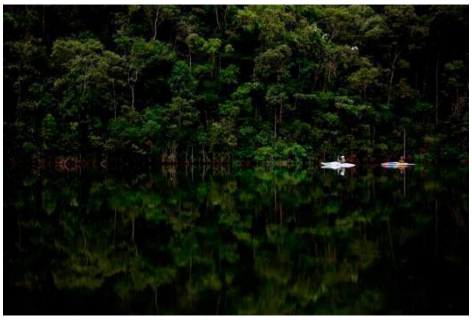

PHOTO ID: 121
Valence mean (95% CI): 6.9 (6.6; 7.2)
Arousal mean (95% CI): 4.7 (4.2; 5.2)

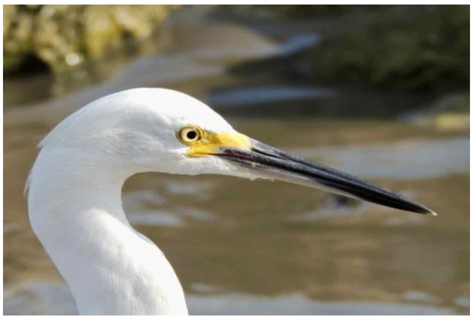

PHOTO ID: 149
Valence mean (95% CI): 6.5 (6.1; 6.9)
Arousal mean (95% CI): 4.3 (3.9; 4.8)

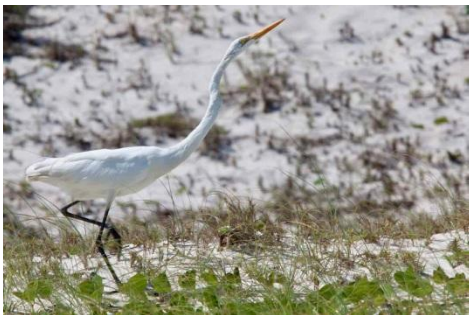

PHOTO ID: 163
Valence mean (95% CI): 7.0 (6.6; 7.4)
Arousal mean (95% CI): 4.8 (4.2; 5.4)

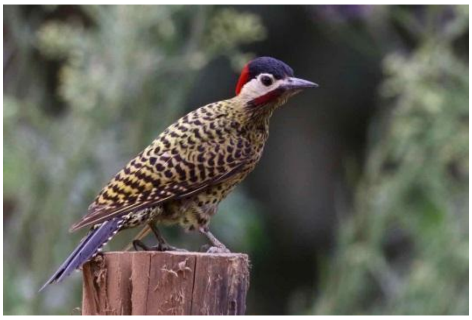

PHOTO ID: 196

Valence mean (95% CI): 6.6 (6.1; 7.0)
Arousal mean (95% CI): 4.4 (3.9; 5.0)

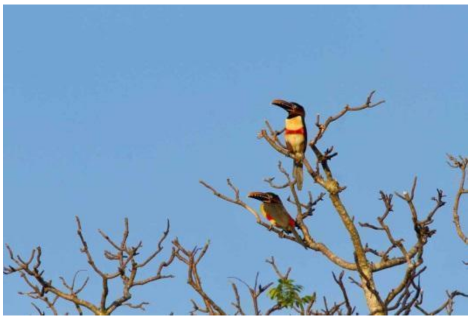

PHOTO ID: 204
Valence mean (95% CI): 6.4 (6.0; 6.9)
Arousal mean (95% CI): 4.2 (3.7; 4.8)

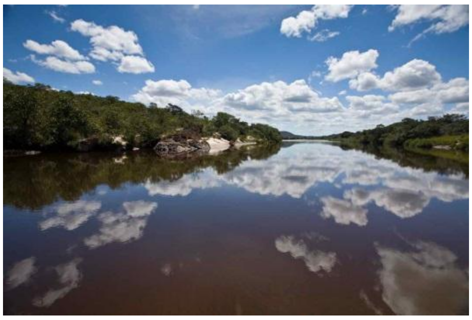

PHOTO ID: 66
Valence mean (95% CI): 6.3 (6.0; 6.6)
Arousal mean (95% CI): 4.2 (3.8; 4.7)

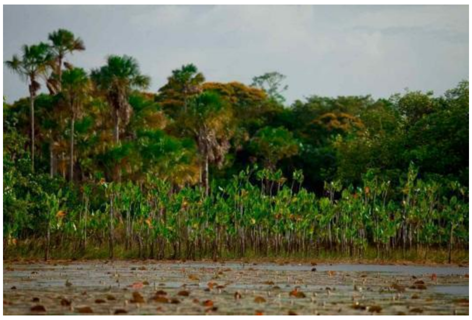

PHOTO ID: 83

Valence mean (95% CI): 6.4 (6.1; 6.8)
Arousal mean (95% CI): 4.3 (3.9; 4.7)

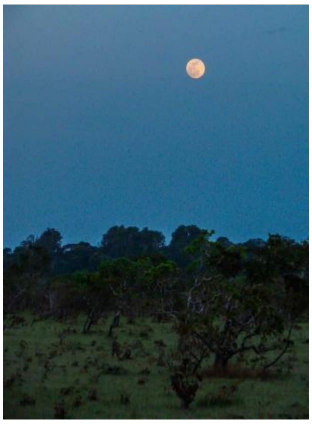

PHOTO ID: 99
Valence mean (95% CI): 6.3 (5.9; 6.7)
Arousal mean (95% CI): 4.2 (3.8; 4.6)

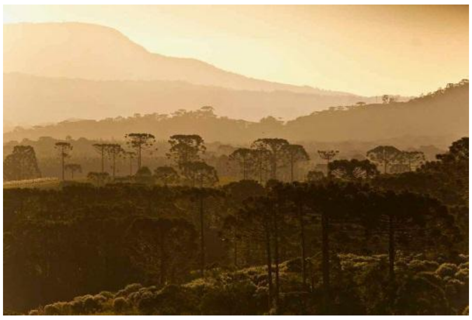

PHOTO ID: 297
Valence mean (95% CI): 6.8 (6.5; 7.2)
Arousal mean (95% CI): 4.7 (4.2; 5.3)

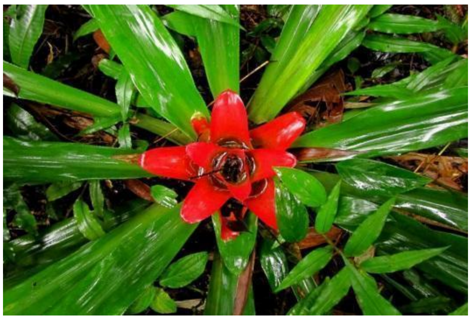

PHOTO ID: 301

Valence mean (95% CI): 6.3 (5.9; 6.7)
Arousal mean (95% CI): 4.2 (3.7; 4.7)

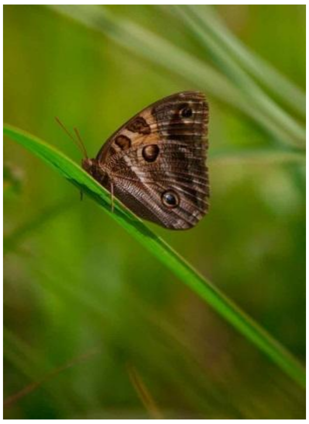

PHOTO ID: 320
Valence mean (95% CI): 6.4 (6.0; 6.8)
Arousal mean (95% CI): 4.3 (3.8; 4.7)

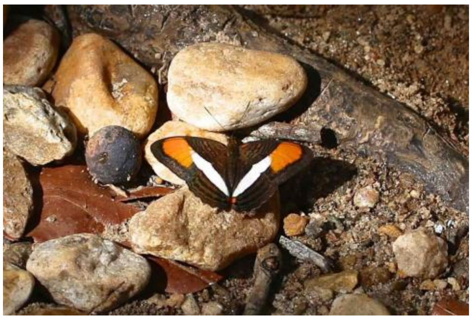

PHOTO ID: 7
Valence mean (95% CI): 6.7 (6.3; 7.2)
Arousal mean (95% CI): 4.6 (4.1; 5.3)

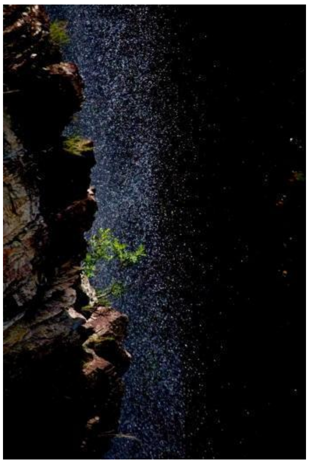

PHOTO ID: 124

Valence mean (95% CI): 6.5 (6.1; 6.9)
Arousal mean (95% CI): 4.4 (3.9; 4.9)

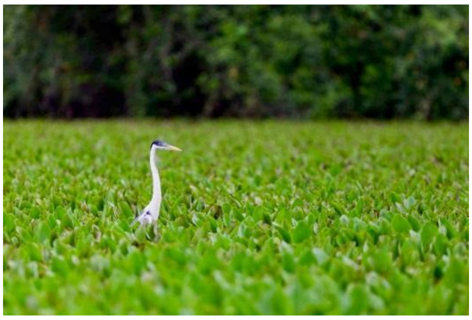

PHOTO ID: 158
Valence mean (95% CI): 6.7 (6.3; 7.1)
Arousal mean (95% CI): 4.6 (4.1; 5.1)

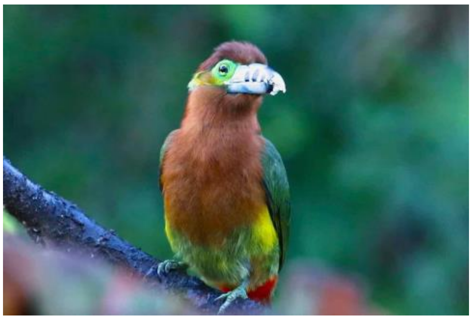

PHOTO ID: 70
Valence mean (95% CI): 6.7 (6.5; 7.0)
Arousal mean (95% CI): 4.7 (4.2; 5.1)

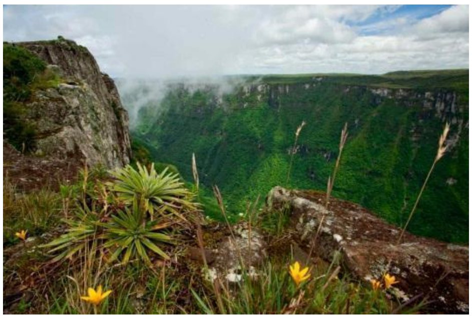

PHOTO ID: 74
Valence mean (95% CI): 6.4 (6.1; 6.8)
Arousal mean (95% CI): 4.4 (4.1; 4.9)

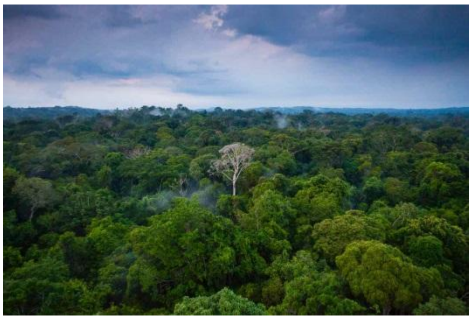

PHOTO ID: 352

Valence mean (95% CI): 6.8 (6.4; 7.2)
Arousal mean (95% CI): 4.8 (4.2; 5.4)

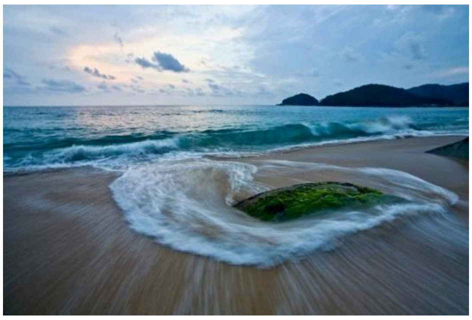

PHOTO ID: 353
Valence mean (95% CI): 6.4 (5.9; 6.9)
Arousal mean (95% CI): 4.4 (3.8; 5.0)

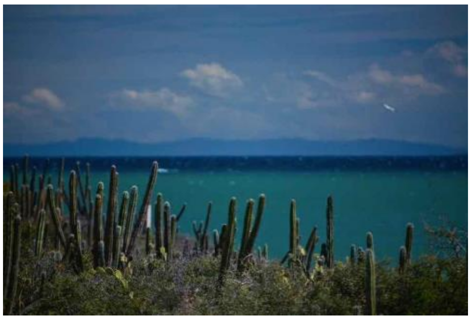

PHOTO ID: 366
Valence mean (95% CI): 6.8 (6.4; 7.1)
Arousal mean (95% CI): 4.8 (4.2; 5.6)

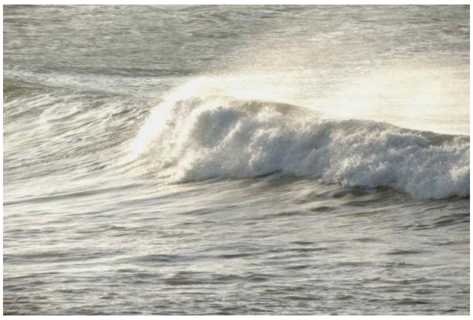

PHOTO ID: 102
Valence mean (95% CI): 6.4 (5.9; 6.8)
Arousal mean (95% CI): 4.5 (4.0; 5.0)

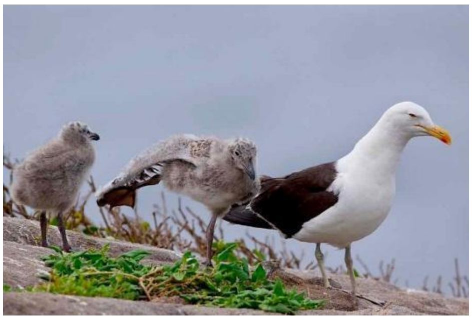

PHOTO ID: 350

Valence mean (95% CI): 6.5 (6.2; 6.9)
Arousal mean (95% CI): 4.6 (4.1; 5.2)

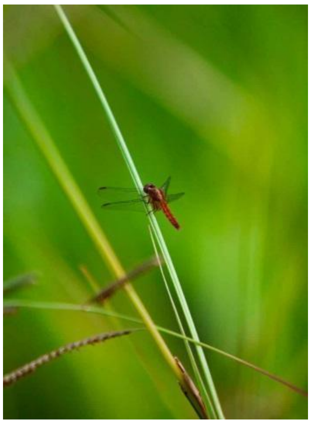

PHOTO ID: 73
Valence mean (95% CI): 6.6 (6.3; 6.9)
Arousal mean (95% CI): 4.7 (4.3; 5.1)

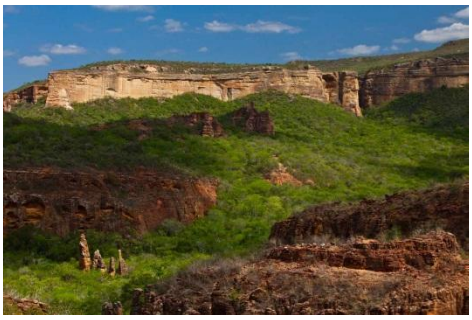

PHOTO ID: 78
Valence mean (95% CI): 6.6 (6.3; 6.8)
Arousal mean (95% CI): 4.7 (4.4; 5.1)

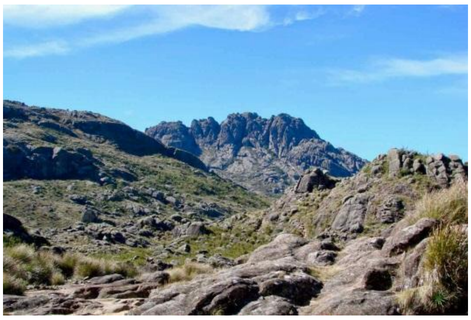

PHOTO ID: 109

Valence mean (95% CI): 6.2 (5.8; 6.7)
Arousal mean (95% CI): 4.3 (3.8; 4.9)

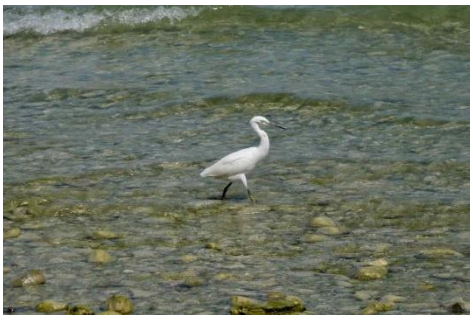

PHOTO ID: 359
Valence mean (95% CI): 6.7 (6.3; 7.1)
Arousal mean (95% CI): 4.8 (4.2; 5.5)

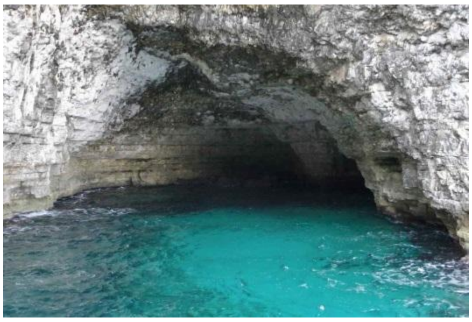

PHOTO ID: 209
Valence mean (95% CI): 6.1 (5.7; 6.7)
Arousal mean (95% CI): 4.3 (3.7; 4.8)

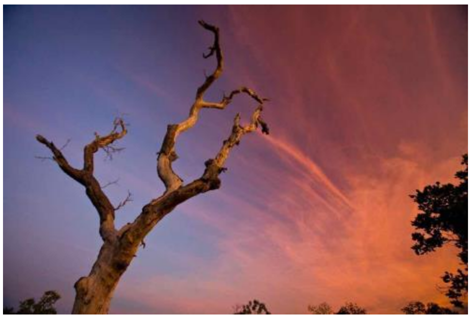

PHOTO ID: 312
Valence mean (95% CI): 6.5 (6.1; 6.9)
Arousal mean (95% CI): 4.7 (4.2; 5.2)

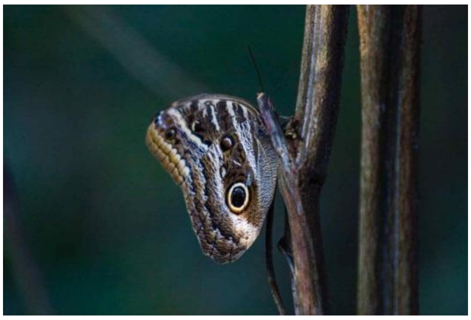

PHOTO ID: 217

Valence mean (95% CI): 5.8 (5.3; 6.3)
Arousal mean (95% CI): 4.1 (3.6; 4.8)

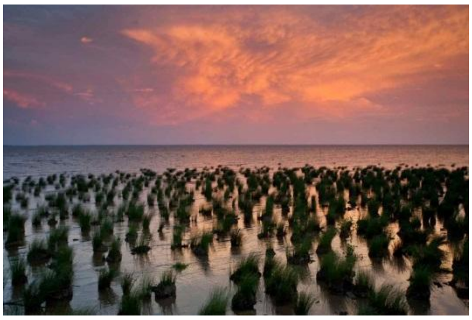

PHOTO ID: 62
Valence mean (95% CI): 6.5 (6.1; 6.9)
Arousal mean (95% CI): 4.8 (4.4; 5.3)

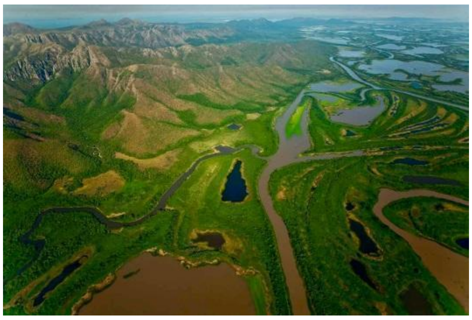

PHOTO ID: 101
Valence mean (95% CI): 6.6 (6.2; 6.9)
Arousal mean (95% CI): 4.9 (4.3; 5.5)

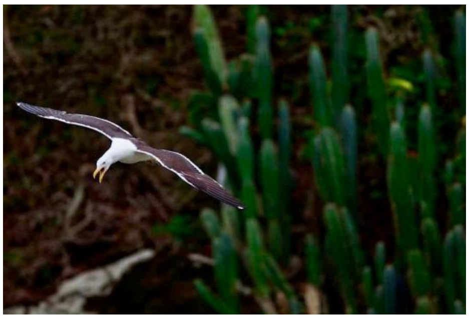

PHOTO ID: 123

Valence mean (95% CI): 6.6 (6.2; 7.1)
Arousal mean (95% CI): 4.9 (4.4; 5.5)

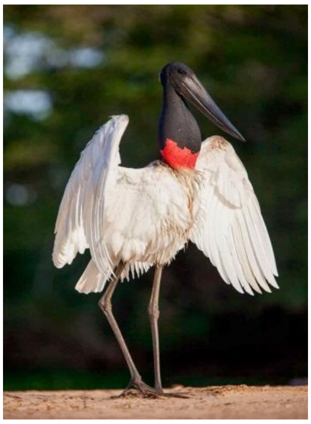

PHOTO ID: 133
Valence mean (95% CI): 6.5 (6.1; 6.9)
Arousal mean (95% CI): 4.8 (4.3; 5.4)

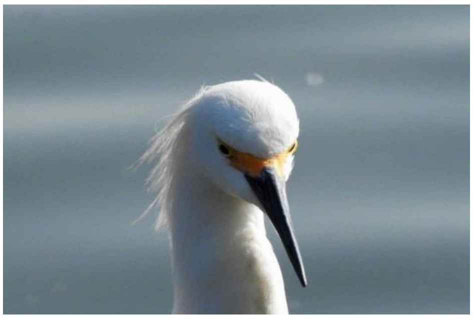

PHOTO ID: 185
Valence mean (95% CI): 6.1 (5.7; 6.5)
Arousal mean (95% CI): 4.4 (3.8; 5.0)

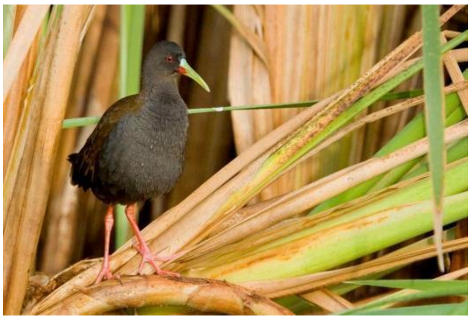

PHOTO ID: 51

Valence mean (95% CI): 6.3 (5.9; 6.7)
Arousal mean (95% CI): 4.7 (4.3; 5.2)

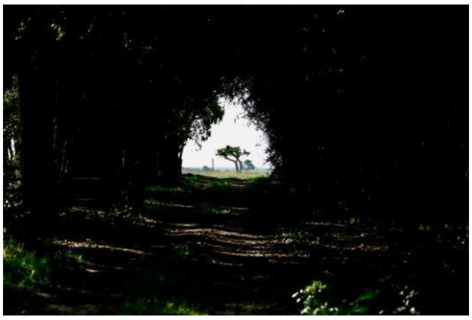

PHOTO ID: 316
Valence mean (95% CI): 6.4 (6.0; 6.9)
Arousal mean (95% CI): 4.8 (4.3; 5.5)

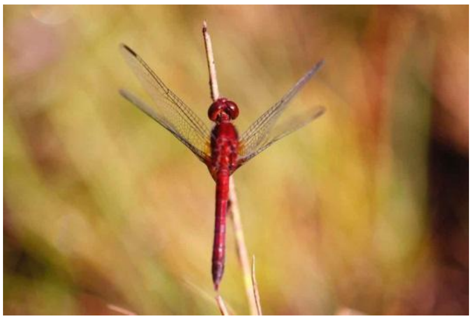

PHOTO ID: 84
Valence mean (95% CI): 6.2 (5.8; 6.5)
Arousal mean (95% CI): 4.6 (4.2; 5.1)

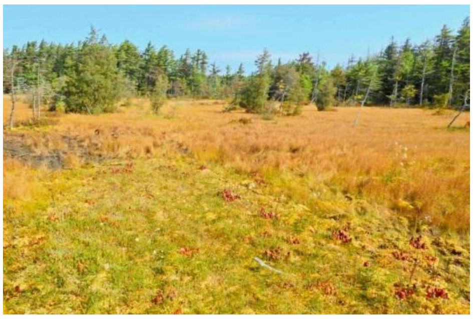

PHOTO ID: 85
Valence mean (95% CI): 6.1 (5.8; 6.5)
Arousal mean (95% CI): 4.5 (4.1; 5.0)

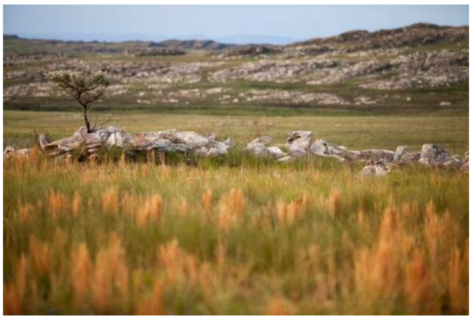

PHOTO ID: 376

Valence mean (95% CI): 6.5 (6.1; 7.0)
Arousal mean (95% CI): 4.9 (4.4; 5.5)

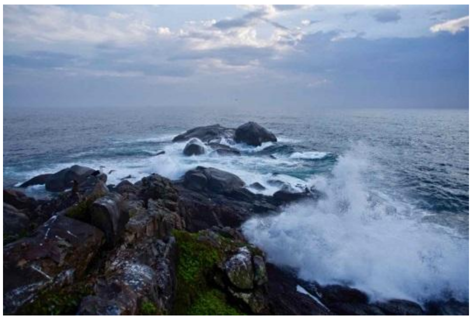

PHOTO ID: 76
Valence mean (95% CI): 5.9 (5.6; 6.3)
Arousal mean (95% CI): 4.4 (4.0; 4.8)

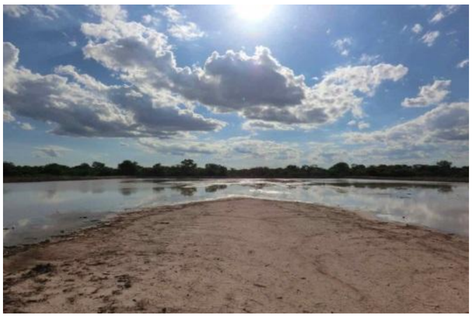

PHOTO ID: 89
Valence mean (95% CI): 5.9 (5.5; 6.3)
Arousal mean (95% CI): 4.4 (3.9; 4.9)

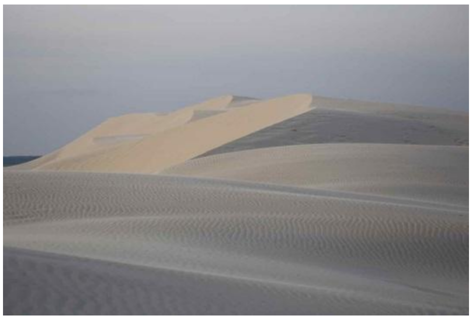

PHOTO ID: 362
Valence mean (95% CI): 6.4 (5.9; 6.8)
Arousal mean (95% CI): 4.9 9 (4.3; 5.4)

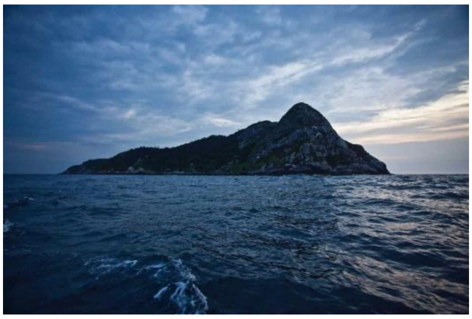

PHOTO ID: 375

Valence mean (95% CI): 6.2 (5.7; 6.7)
Arousal mean (95% CI): 4.8 (4.3; 5.4)

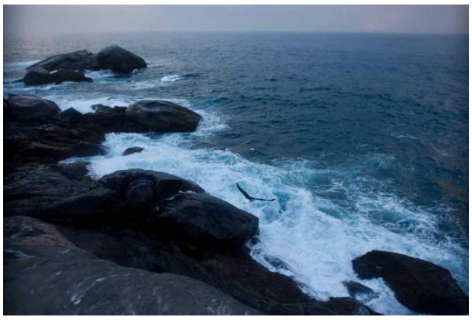

PHOTO ID: 14
Valence mean (95% CI): 5.8 (5.4; 6.3)
Arousal mean (95% CI): 4.6 (4.0; 5.3)

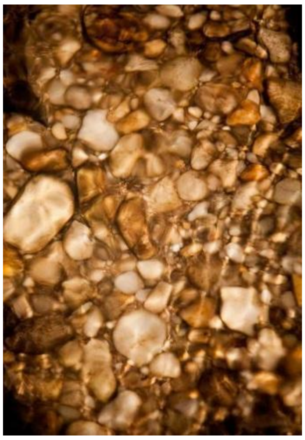

PHOTO ID: 91
Valence mean (95% CI): 6.0 (5.6; 6.4)
Arousal mean (95% CI): 4.8 (4.3; 5.4)

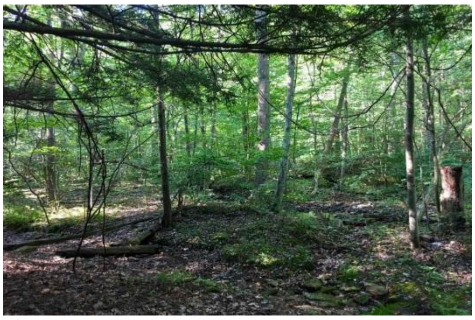

PHOTO ID: 127

Valence mean (95% CI): 6.0 (5.5; 6.5)
Arousal mean (95% CI): 4.9 (4.3; 5.6)

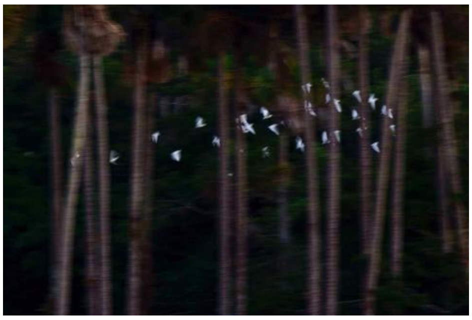

PHOTO ID: 59
Valence mean (95% CI): 5.8 (5.5; 6.2)
Arousal mean (95% CI): 4.8 (4.3; 5.3)

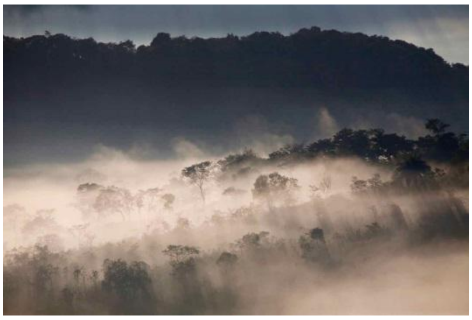

PHOTO ID: 293
Valence mean (95% CI): 5.7 (5.3; 6.1)
Arousal mean (95% CI): 4.7 (4.2; 5.3)

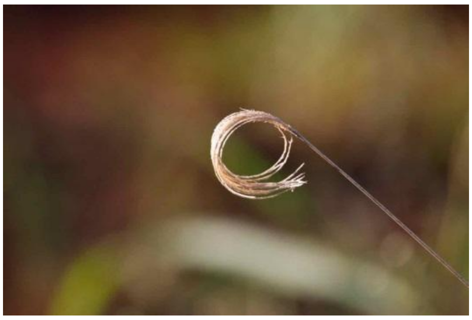

PHOTO ID: 335
Valence mean (95% CI): 5.6 (5.1; 6.1)
Arousal mean (95% CI): 4.6 (4.0; 5.2)

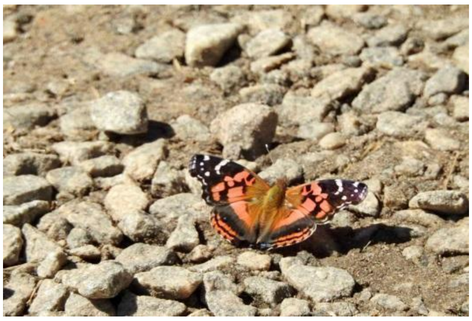

QUADRANT 2

PHOTO ID: 152
Valence mean (95% CI): 7.0 (6.7; 7.3)
Arousal mean (95% CI): 5.0 (4.5; 5.5)

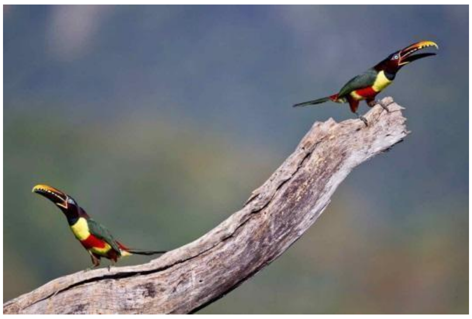

PHOTO ID: 302
Valence mean (95% CI): 6.9 (6.5; 7.3)
Arousal mean (95% CI): 5.0 (4.5; 5.6)

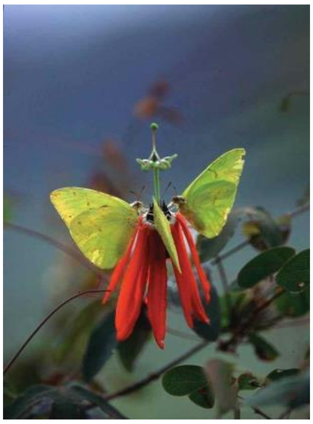

PHOTO ID: 368
Valence mean (95% CI): 7.2 (6.9; 7.6)
Arousal mean (95% CI): 5.3 (4.8; 6.0)

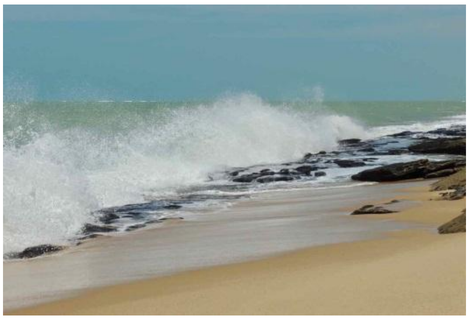

PHOTO ID: 29
Valence mean (95% CI): 6.9 (6.5; 7.3)
Arousal mean (95% CI): 5.1 (4.6; 5.7)

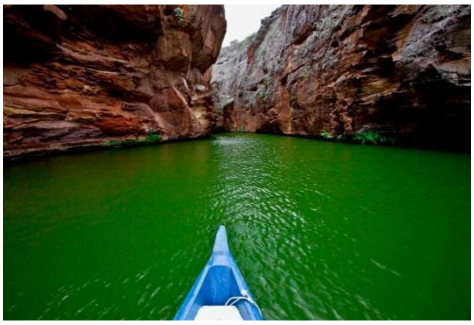

PHOTO ID: 363
Valence mean (95% CI): 7.2 (6.9; 7.6)
Arousal mean (95% CI): 5.6 (4.9; 6.4)

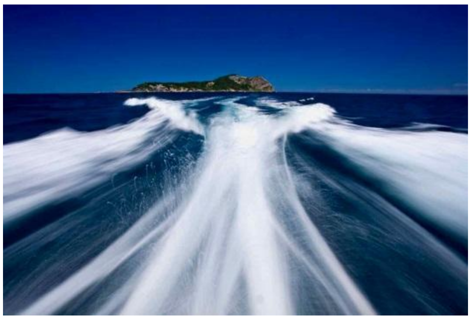

PHOTO ID: 8
Valence mean (95% CI): 6.8 (6.4; 7.3)
Arousal mean (95% CI): 5.3 (4.7; 5.9)

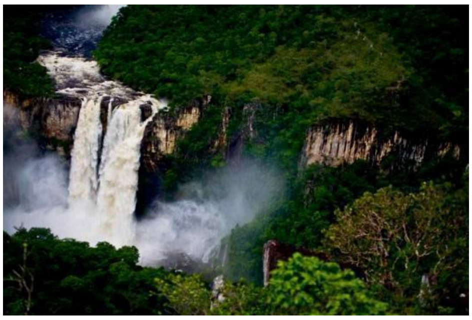

PHOTO ID: 23
Valence mean (95% CI): 6.6 (6.3; 7.0)
Arousal mean (95% CI): 5.1 (4.5; 5.8)

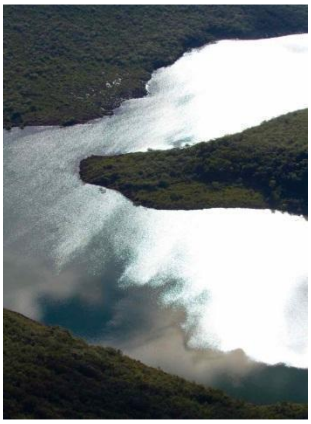

PHOTO ID: 44
Valence mean (95% CI): 6.9 (6.5; 7.2)
Arousal mean (95% CI): 5.4 (4.8; 6.1)

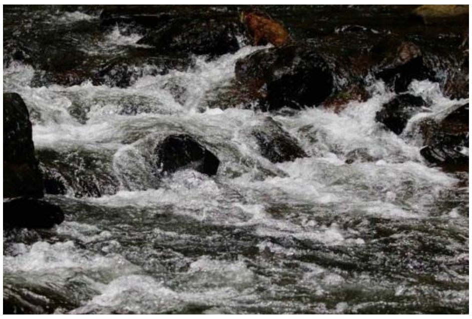

PHOTO ID: 144
Valence mean (95% CI): 6.5 (6.1; 6.9)
Arousal mean (95% CI): 5.1 (4.6; 5.7)

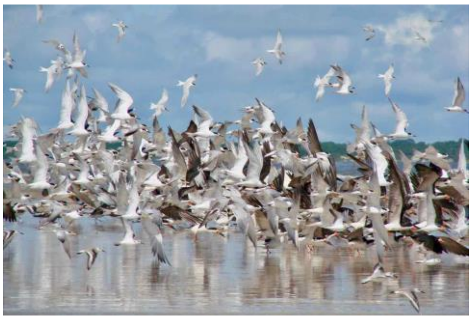

PHOTO ID: 129
Valence mean (95% CI): 6.7 (6.3; 7.1)
Arousal mean (95% CI): 5.3 (4.8; 5.9)

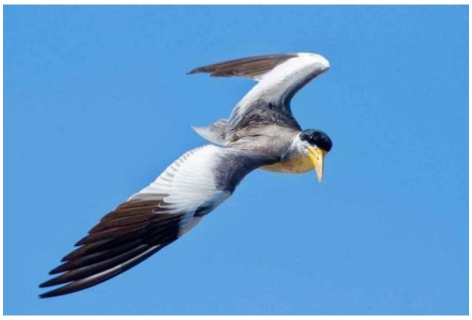

PHOTO ID: 17
Valence mean (95% CI): 6.3 (5.8; 6.7)
Arousal mean (95% CI): 5.0 (4.4; 5.7)

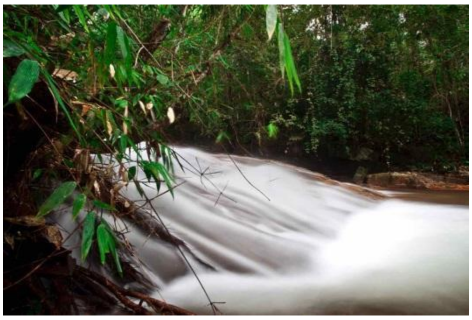

PHOTO ID: 96
Valence mean (95% CI): 6.4 (6.1; 6.8)
Arousal mean (95% CI): 5.1 (4.6; 5.6)

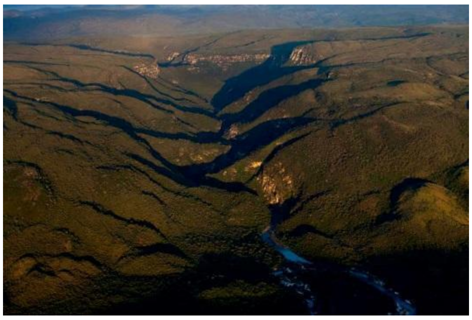

PHOTO ID: 480
Valence mean (95% CI): 6.5 (6.2; 6.9)
Arousal mean (95% CI): 5.2 (4.8; 5.7

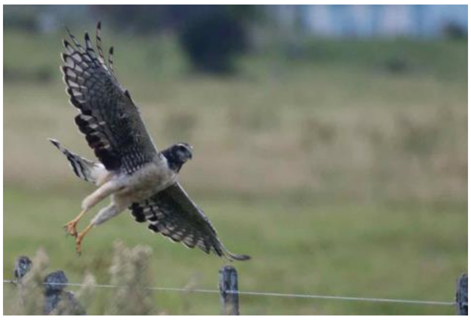

PHOTO ID: 65
Valence mean (95% CI): 6.3 (5.9; 6.7)
Arousal mean (95% CI): 5.1 (4.6; 5.6)

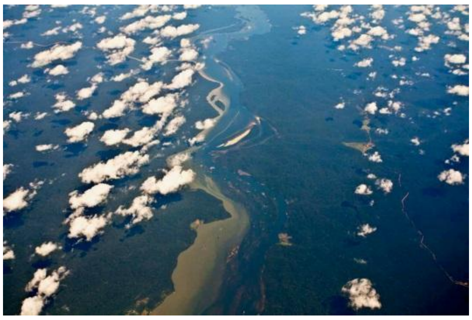

PHOTO ID: 271
Valence mean (95% CI): 6.3 (5.9; 6.7)
Arousal mean (95% CI): 5.1 (4.5; 5.8)

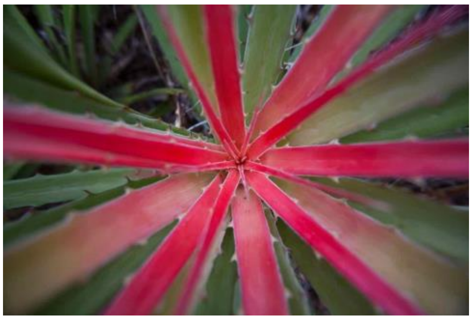

PHOTO ID: 357
Valence mean (95% CI): 6.5 (6.0; 6.9)
Arousal mean (95% CI): 5.3 (4.7; 6.0)

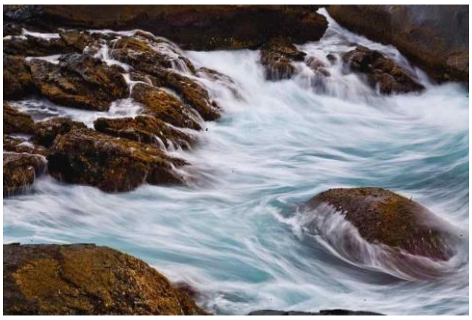

PHOTO ID: 303
Valence mean (95% CI): 6.4 (6.0; 6.7)
Arousal mean (95% CI): 5.3 (4.8; 5.9)

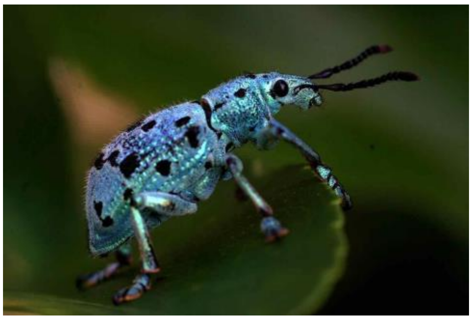

PHOTO ID: 18
Valence mean (95% CI): 6.5 (6.0; 7.0)
Arousal mean (95% CI): 5.4 (4.9; 6.1)

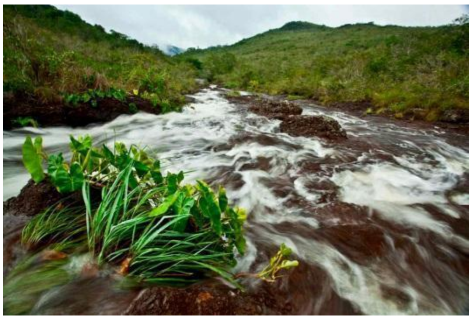

PHOTO ID: 295
Valence mean (95% CI): 6.1 (5.6; 6.5)
Arousal mean (95% CI): 5.0 (4.4; 5.7)

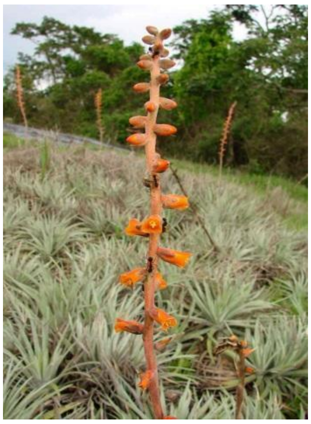

PHOTO ID: 35
Valence mean (95% CI): 6.6 (6.2; 7.1)
Arousal mean (95% CI): 5.6 (5.0; 6.2)

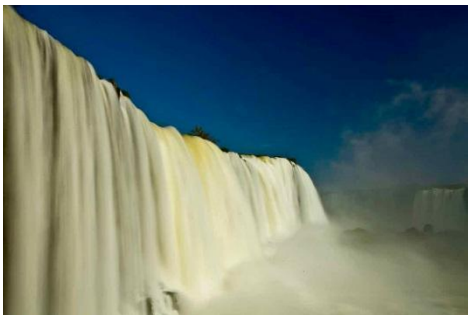

PHOTO ID: 373
Valence mean (95% CI): 6.8 (6.5; 7.2)
Arousal mean (95% CI): 5.8 (5.2; 6.5)

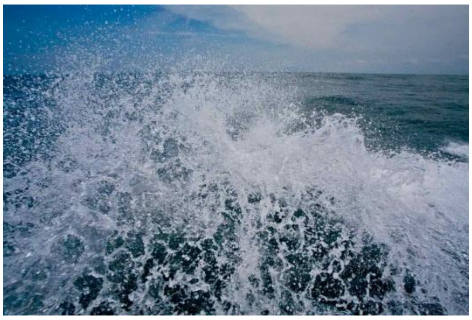

PHOTO ID: 305
Valence mean (95% CI): 6.2 (5.8; 6.7)
Arousal mean (95% CI): 5.3 (4.7; 6.0)

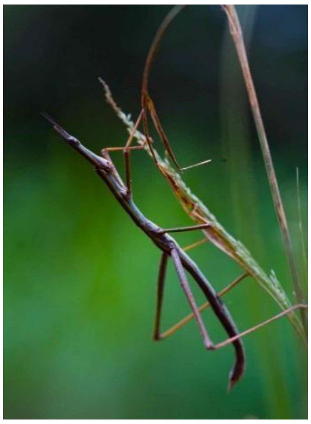

PHOTO ID: 351
Valence mean (95% CI): 5.8 (5.3; 6.4)
Arousal mean (95% CI): 5.1 (4.6; 5.6)

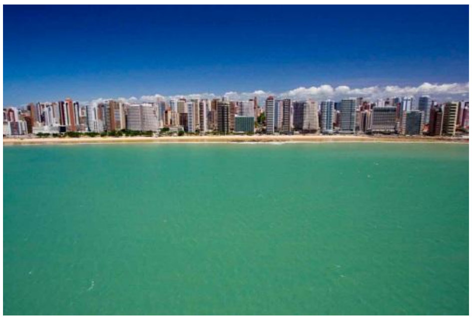

PHOTO ID: 10
Valence mean (95% CI): 5.9 (5.4; 6.4)
Arousal mean (95% CI): 5.2 (4.7; 5.8)

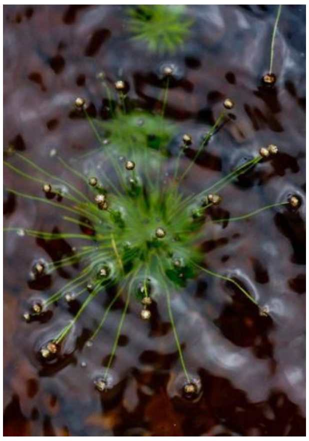

PHOTO ID: 306
Valence mean (95% CI): 5.9 (5.5; 6.4)
Arousal mean (95% CI): 5.2 (4.6; 5.8)

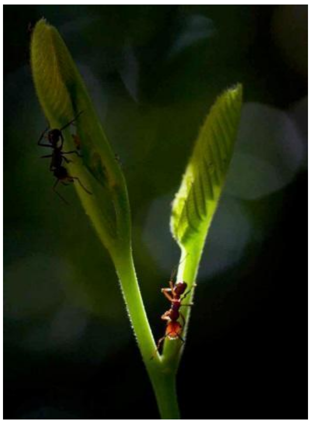

PHOTO ID: 358
Valence mean (95% CI): 6.0 (5.7; 6.4)
Arousal mean (95% CI): 5.3 (4.8; 6.0)

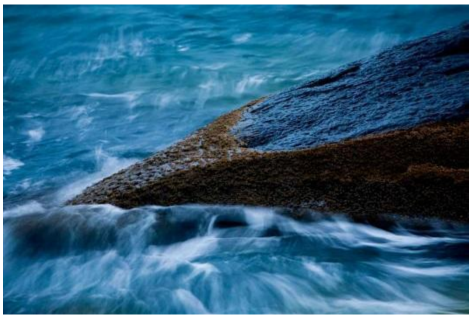

PHOTO ID: 93
Valence mean (95% CI): 5.8 (5.4; 6.2)
Arousal mean (95% CI): 5.3 (4.8; 5.7)

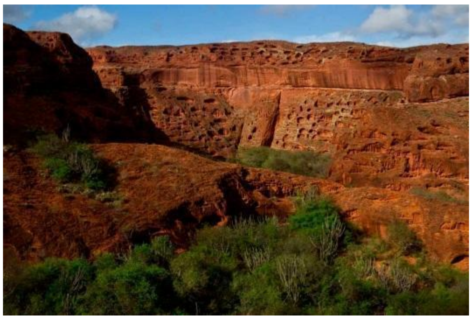

PHOTO ID: 94
Valence mean (95% CI): 5.7 (5.2; 6.2)
Arousal mean (95% CI): 5.2 (4.7; 5.8)

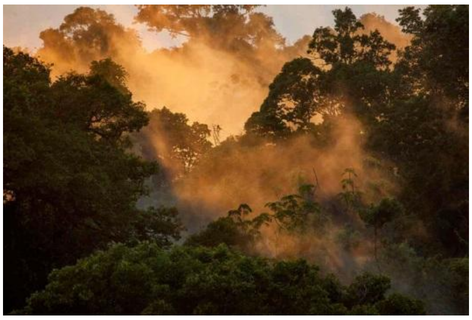

PHOTO ID: 370
Valence mean (95% CI): 6.4 (6.0; 6.9)
Arousal mean (95% CI): 6.0 (5.4; 6.6)

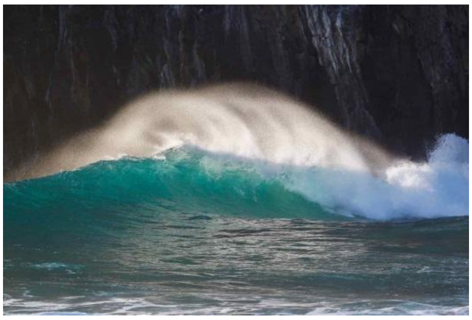

PHOTO ID: 33
Valence mean (95% CI): 5.7 (5.2; 6.2)
Arousal mean (95% CI): 5.3 (4.7; 5.9)

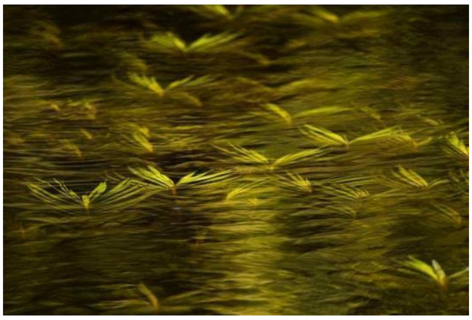

PHOTO ID: 313
Valence mean (95% CI): 6.1 (5.6; 6.6)
Arousal mean (95% CI): 5.7 (5.2; 6.4)

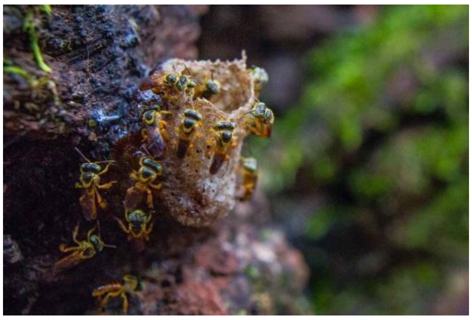

PHOTO ID: 336
Valence mean (95% CI): 5.9 (5.5; 6.4)
Arousal mean (95% CI): 5.6 (5.0; 6.4)

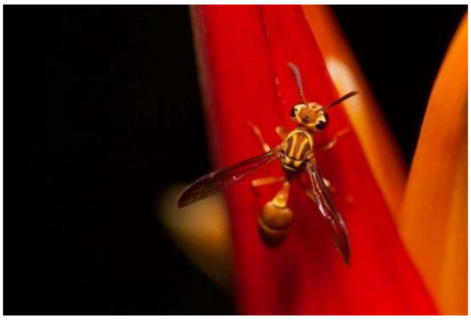

PHOTO ID: 210
Valence mean (95% CI): 5.9 (5.4; 6.5)
Arousal mean (95% CI): 5.9 (5.2; 6.8)

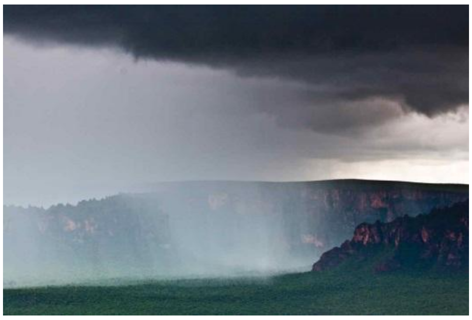

PHOTO ID: 479
Valence mean (95% CI): 6.3 (5.9; 6.6)
Arousal mean (95% CI): 6.3 (5.8; 6.9)

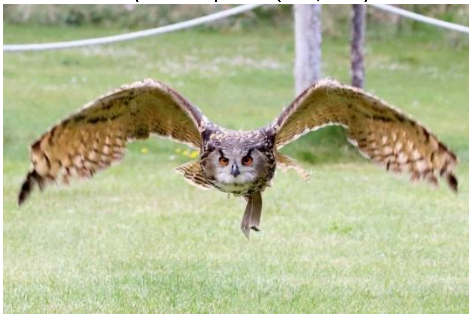

PHOTO ID: 11
Valence mean (95% CI): 5.5 (5.0; 6.0)
Arousal mean (95% CI): 5.9 (5.4; 6.5)

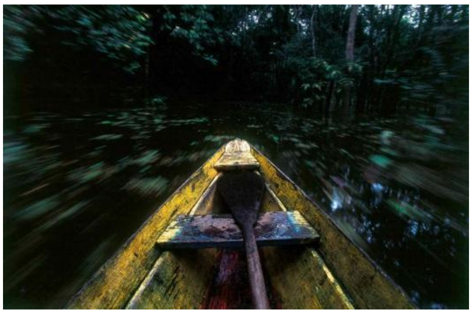

PHOTO ID: 26
Valence mean (95% CI): 5.5 (5.0; 6.1)
Arousal mean (95% CI): 6.1 (5.4; 6.8)

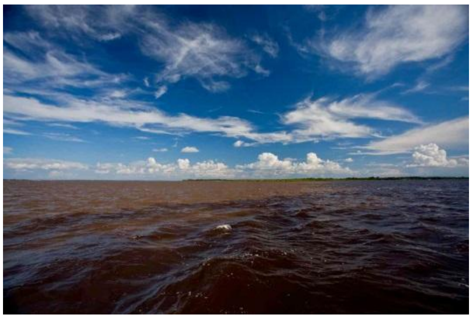

PHOTO ID: 339
Valence mean (95% CI): 5.2 (4.8; 5.6)
Arousal mean (95% CI): 5.8 (5.2; 6.5)

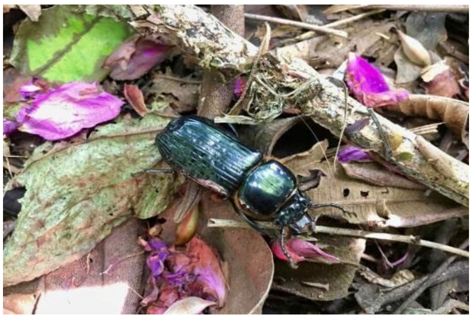

PHOTO ID: 304
Valence mean (95% CI): 5.3 (4.9; 5.7)
Arousal mean (95% CI): 6.1 (5.6; 6.8)

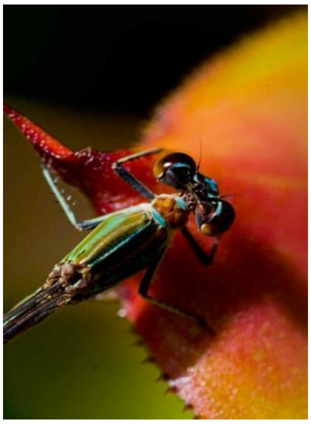

PHOTO ID: 356
Valence mean (95% CI): 5.7 (5.2; 6.3)
Arousal mean (95% CI): 6.7 (6.1; 7.4)

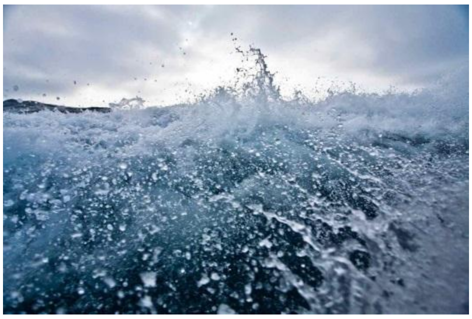

QUADRANT 3

No images

QUADRANT 4

PHOTO ID: 25

Valence mean (95% CI): 4.3 (3.7; 5.0)

Arousal mean (95% CI): 6.5 (5.9; 7.3)

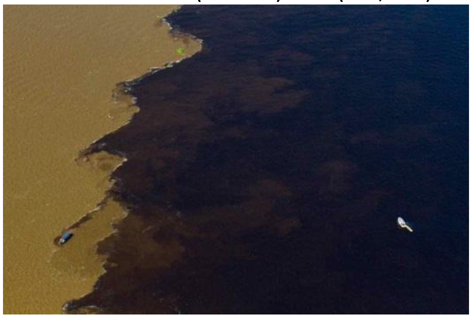

IMAGE AUTHORS

Eliseth RIbeiro Leão: 39, 42, 43, 44, 45, 48, 51, 52, 53, 54, 88, 89, 108, 109, 110, 111, 114, 115, 116, 117, 118, 119, 120, 121, 122, 134, 135, 137, 138, 139, 140, 155, 156, 157, 158, 160, 163, 165, 166, 167, 168, 169, 173,174, 175, 176, 177, 178, 179, 182, 183, 200, 223, 224, 228, 237, 240, 241, 261, 262, 263, 264, 265, 266, 279, 280, 281, 283, 284, 285, 286, 287, 288, 289, 290, 292, 300, 317, 318, 319, 320, 321, 322, 326, 327, 328, 329, 330, 337, 338, 340, 347,348, 349, 359, 360, 361, 366, 367, 368, 369, 377, 378, 379, 384, 385, 386, 388, 389, 390, 391, 392, 394, 395, 396, 399, 479 and 480.

Luciano Lima: 49, 77, 78, 79, 80, 81, 82, 83, 86, 87, 90, 141, 142, 143, 144, 145, 146, 147, 148, 149, 150, 184, 185, 186, 187, 188, 189, 190, 191, 192, 193, 194, 195, 196, 197, 198, 199, 242, 243, 244, 245, 246, 247, 248, 249, 250, 294, 295, 296, 297, 298, 299, 380, 397, 398, 400, 443 and 447.

João Rosa: 1, 2, 3, 4, 5, 6, 7, 8, 9, 10, 11, 12, 13, 14, 15, 16, 17, 18, 19, 20, 21, 22, 23, 24, 25, 26, 27, 28, 29, 30, 31, 32, 33, 34, 35, 36, 40, 46, 56, 57, 58, 59, 60, 62, 63, 64, 65, 66, 67, 68, 69, 70, 71, 72, 73, 74, 75, 85, 93, 94, 95, 96, 97, 98, 99, 101, 102, 103, 104, 105, 106, 107, 113, 123, 124, 125, 127, 128, 129, 130, 131, 132, 136, 151, 152, 153, 154, 159, 161, 162, 164, 170, 171, 180, 181, 201, 202, 203, 204, 205, 206, 207, 208, 209, 210, 211, 212, 213, 214, 215, 216, 217, 218, 219, 220, 221, 222, 225, 227, 231, 232, 233, 234, 235, 236, 251, 252, 253, 254, 255, 256, 257, 258, 259, 260, 267, 268, 269, 270, 271, 272, 273, 274, 275, 276, 277, 278, 282, 291, 301, 302, 303, 304, 305, 306, 307, 308, 309, 310, 311, 312, 313, 314, 316, 323, 324, 325, 336, 350, 351, 352, 353, 354, 355, 356, 357, 358, 362, 363, 364, 365, 370, 371, 372, 373, 374, 375, 376 and 406.

Erika Hingst-Zaher: 37, 38, 41, 47, 55, 76, 84, 91, 92, 126, 133, 172, 226, 229, 230, 238, 239, 334, 335, 339, 381, 382, 383, 387 and 393.

Daniela Dal Fabbro: 50, 100 and 448.
