# Supplementary material for: e-Nature Positive Emotions Photography Database (e-NatPOEM): affectively rated nature images promoting positive emotions
Source: Sci Rep. 2021 Jun 3;11:11696. doi: 10.1038/s41598-021-91013-9 (PMC8175760; doi:10.1038/s41598-021-91013-9)
Supplement: Supplementary file 4 — Supplementary Information 4. [file 41598_2021_91013_MOESM4_ESM.pdf]

Supplementary Material 4. Pictures with discrepancies in valence and arousal ratings and image attributes assigned by participants.

e-Nature Positive Emotions Photography Database (e-NatPOEM) - affectively rated nature images promoting positive emotions

Daniela Dal Fabbro, Giulia Catissi, Gustavo Borba, Luciano Lima, Erika Hingst-Zaher, João Rosa, Elivane Victor, Leticia Oliveira, Tinely Souza, Eliseth Leão.

Photo ID	Category	Valence Mean (95% CI)	Arousal Mean (95% CI)	Attribute	Image
356	Sea	5.7 (5.2; 6.3)	6.7 (6.1; 7.4)	Fear	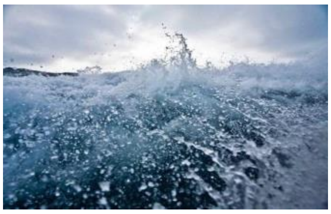
357	Sea	6.5 (6.0; 6.9)	5.3 (4.7; 6.0)	Fear	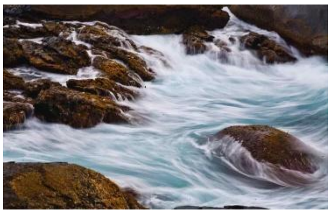
359	Sea	6.7 (6.3; 7.1)	4.8 (4.2; 5.5)	Fear	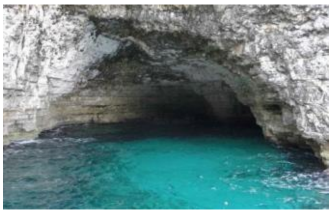
366	Sea	6.8 (6.4; 7.1)	4.8 (4.2; 5.6)	Fear	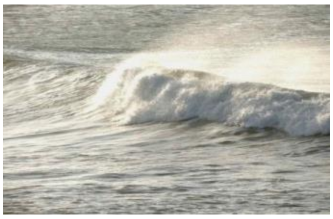
6	Water	6.4 (6.0; 6.9)	3.9 (3.4; 4.4)	Loneliness	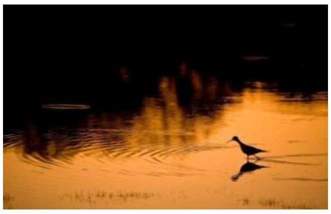

11	Water	5.5<br>(5.0; 6.0)	5.9<br>(5.4; 6.5)	Fear	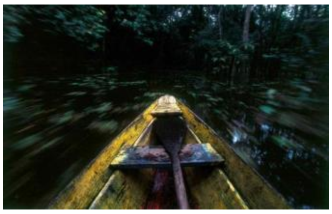
25	Water	4.3<br>(3.7; 5.0)	6.5<br>(5.9; 7.3)	Sadness	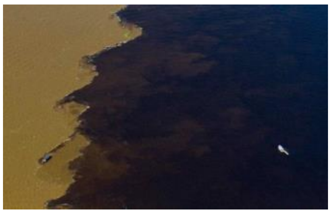
340	Insect	6.3<br>(5.8; 6.8)	3.9<br>(3.4; 4.5)	Loneliness	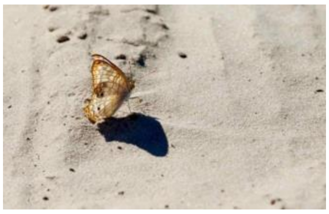
336	Insect	5.9<br>(5.5; 6.4)	5.6<br>(5.0; 6.4)	Fear	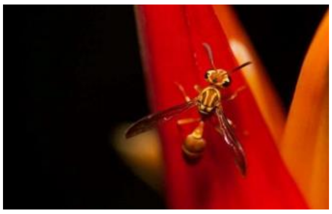
136	Pale bird	6.8<br>(6.4; 7.2)	3.9<br>(3.4; 4.4)	Loneliness	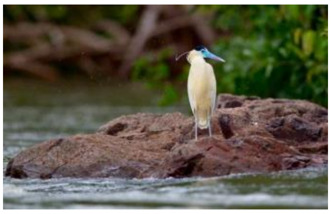
298	Flower	6.8<br>(6.4; 7.1)	3.7<br>(3.3; 4.2)	Loneliness	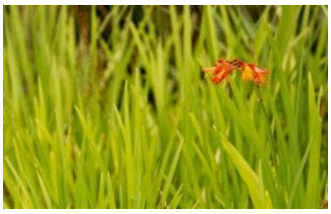
